# Organocatalytic Enantio- and Diastereoselective Synthesis of Dispirooxindole Derivatives with Adjacent Spirocyclic Centers via [4 + 2] Cycloadditions

**DOI:** 10.1021/acs.joc.6c00103

**Published:** 2026-04-06

**Authors:** Raquel Hidalgo-León, José Trujillo-Sierra, José Miguel Sansano, María de Gracia Retamosa

**Affiliations:** Departamento de Química Orgánica, Centro de Innovación en Química Avanzada (ORFEO–CINQA) and Institute of Organic Synthesis, 16718 Universidad de Alicante, Ctra. Alicante-San Vicente s/n, Alicante 03080, Spain

## Abstract

The highly diastereo- and enantioselective organocatalytic synthesis of six-membered ring dispirocyclohexane oxindole derivatives with adjacent spirocyclic centers via [4 + 2] cycloadditions is described. The combination of a cinchonidine-derived primary amine catalyst with *p*-methylbenzoic acid as a cocatalyst allows access to a broad range of dispirocyclohexane oxindoles. The influence of substituents on the reaction outcome was thoroughly evaluated, including those at the *N*-position of the oxindole and those at the terminal carbon of the C=C double bond.

## Graphical abstract

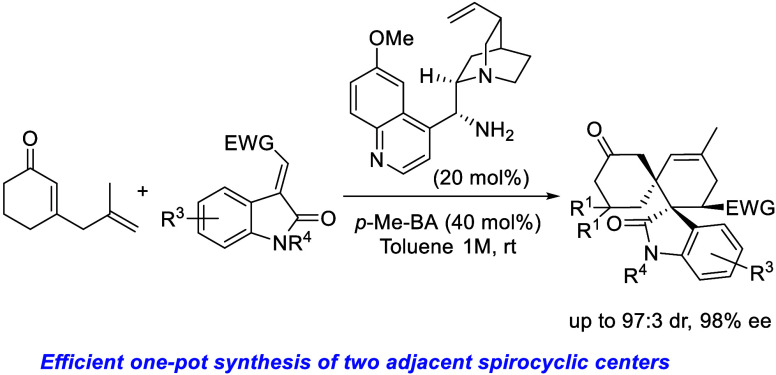

## Introduction

Spirooxindoles^1^ have emerged as a privileged scaffold motif due to their remarkable structural complexity and frequent presence in natural products and bioactive compounds.^2^ Figure 1 illustrates a selection of structures, including (A), which is a potent inhibitor for HIV replication; (B),^3^ used in the treatment of Schizophrenia, Parkinson’s, or Huntington’s diseases being a phosphodiesterase inhibitor;^4^ (C), serving as an acetylcholinesterase inhibitor;^5^ (D) (the drug NITD609), known for its antimalarial activity;^6^ (E), which acts as an MDM2 inhibitor with anticancer effects;^7^ and (F), (Satavaptan) acting as a potent and selective antagonist of the vasopressin V2 receptor for the treatment of hyponatremia.^8^ Given the versatility of their pharmaceutical applications, structural modifications of spirooxindoles offer the possibility of improving their pharmacological properties and, therefore, to advance drug discovery. Focused on the synthesis of enantiomerically enriched six-membered ring spirocyclohexane oxindoles, the enantioselective [4 + 2] cycloaddition has emerged as the most significant approach, successfully employed by various research groups.^9^ However, there are no examples in the literature regarding the synthesis of dispirooxindole derivatives with adjacent spirocyclic centers via [4 + 2] cycloaddition, probably due to the unfavorable formation of sterically congested structures containing multiple vicinal spirostereo-centers. Moreover, only one example of a six-membered ring dispirocyclohexane oxindole derivative with adjacent spirocyclic centers has been reported by Chen and co-workers, who prepared it from bisvinylogous 1,4-adducts through a sequential vinylogous iminium–iminium catalytic process.^10^

**Figure 1:**
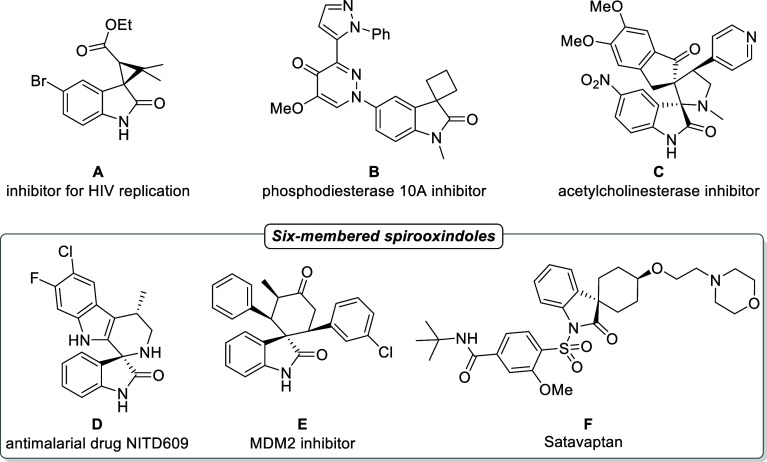
Representative examples of biologically active spirooxindoles derivatives.

On the other hand, distinct reactivity modes arise in trienamine catalysis when cyclic 2,4-dienones or 2,5-dienones are employed, depending on the generation of either cross- or linear-trienamine intermediates. In the case of cyclic 2,4-dienones, the formation of cross-trienamine species has been exploited to achieve α′,β- and γ′,δ-regioselective [4 + 2] cycloaddition reactions.^11^ By contrast, cyclic 2,5-dienones typically produce linear trienamine intermediates, which enable remote functionalization at the ε-position, for instance, through bisvinylogous conjugate addition processes^10,12^ or inverse-electron-demand aza–Diels–Alder reactions.^13^ More recently, our group reported an enantioselective strategy for the preparation of spirocyclic compounds from δ-substituted 2,5-dienones through trienamine catalysis, involving Diels–Alder and aldol/cyclization transformations (Scheme 1a).^14^ In this context, based on our previous work in the design of complex chiral organic molecules and considering that spirooxindoles are present in a wide variety of biologically active compounds, we hypothesized that the use of electron-deficient olefinic oxindole and δ-substituted 2,5-dienones could facilitate the formation of sterically hindered spirooxindole derivatives with adjacent spirocyclic centers via a Diels–Alder reaction (Scheme 1b).

**Scheme 1:**
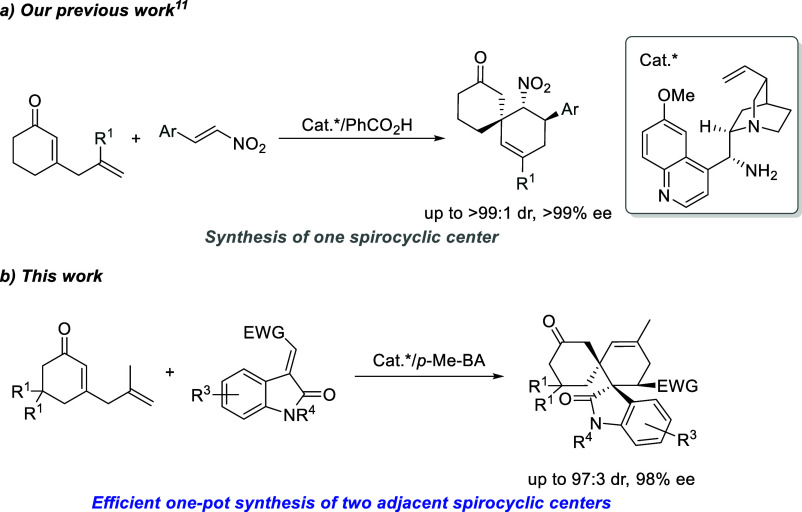
Synthesis of Spirocycle Adducts from 2,5-Dienones

## Results and Discussion

Considering the above factors, the reactivity of 2,5-dienone **1a** with methyleneindolinone **2a** was investigated. The reactions were conducted using different organocatalysts with BA as a coadditive in toluene at room temperature (Table 1, entries 1–6). The proline derivative **I** afforded a complex mixture of different adducts, yielding the desired spirooxindole derivative with good conversion, a high diastereomeric ratio, and a low enantiomeric excess (Table 1, entry 1). Use of the bifunctional amine–sulfonamide **II** and amine–thioureas **III** and **IV** led to the formation of the desired adducts with moderate to high conversions, high diastereoselectivities, and moderate to good enantiomeric excesses (Table 1, entries 2–4). On the other hand, the quinine **V** and quinidine **VI** derivatives led to the formation of spirooxindole derivative **3aa** with high enantiomeric excess, with the quinine derivative **V** providing the best results in terms of conversion and diastereoselectivity (Table 1, entries 5–6). The reaction was further optimized using organocatalyst **V** by adjusting reagent and catalyst loadings and exploring a range of solvents (see the Supporting Information for details). Although varying these parameters had a limited impact, the additive was identified as a key factor affecting both the reaction conversion and the enantioselectivity (Table 1, entries 7–10). While benzoic acid derivatives led to spirocyclic adducts with quantitative conversion, the use of phenol and triethylamine resulted in no reactivity (Table 1, entries 5, 7–8 vs 9–10). All acid derivatives gave similar results in terms of conversion and diastereoselectivity, although salicylic acid produced a decrease in enantioselectivity (Table 1, entry 7). Finally, *p*-methylbenzoic acid was chosen as the best additive because it gave rise to a lower isomerization of the ketone. The optimal reaction temperature was set at 17 °C to ensure reproducibility, allowing the reaction to be scaled up to 0.2 mmol and yielding the desired **3aa** cycloadduct with an excellent diastereomeric ratio and enantiomeric excess (Table 1, entry 11).

**Table 1:**
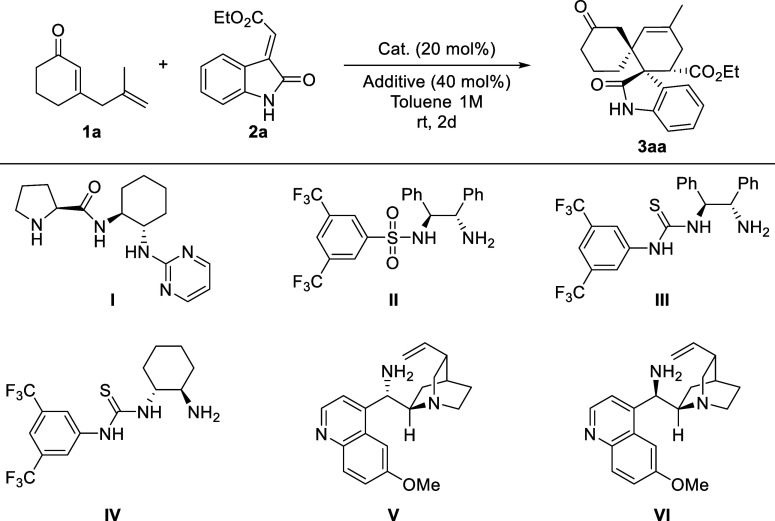
Catalyst and Additive Screening for the β,ε-Regioselective [4 + 2] Cycloaddition^a^

entry	cat	additive	conv [%]^b^	dr^c^	ee [%]^d^
1	**I**	BA	>99^e^	79:21	–10
2	**II**	BA	77	88:12	65
3	**III**	BA	59	86:14	80
4	**IV**	BA^f^	>99	91:9	64
5	**V**	BA	>99	95:5	84
6	**VI**	BA	88	87:3	–84
7	**V**	SA	>99	95:5	76
8	**V**	*p*-Me-BA	>99	95:5	85
9	**V**	PhOH	<5	-	-
10	**V**	Et_3_N	<5	-	-
11[Table-fn t1fn7]	**V**	*p*-Me-BA	>99	95:5^h^	91

a The reactions were performed with ketone **1a** (0.2 mmol), methyleneindolinone **2a** (0.1 mmol), catalyst (20 mol %), and benzoic acid (40 mol %) in toluene (100 μL, 1 M) at room temperature.

b Conversions were measured by ^1^H NMR of crude reaction mixtures considering **2a** limiting reagent after 2 days.

c Diastereomeric ratios were measured by ^1^H NMR of the crude reaction.

d Enantiomeric excesses measured by HPLC correspond to the major enantiomer (1*S*,1′*R*,6′*S*)-**3aa**. Negative values indicate that the opposite enantiomer is formed.

e Complex mixture of products was observed.

f 10% of BA was employed.

h The reactions were performed with ketone **1a** (0.5 mmol), methyleneindolinone **2a** (0.2 mmol), catalyst (20 mol %), and *p*-methylbenzoic acid (40 mol %) in toluene (200 μL, 1 M) at 17 °C.

i Benzoic acid: BA, Salicylic acid: SA, *p*-methylbenzoic acid: *p*-Me-BA.

With the optimal reaction conditions established, we examined the scope of the [4 + 2] cycloaddition. The results of a series of experiments are listed in Scheme 2. Initially, variations in the substituents attached to the phenyl rings of the *N*-unsubstituted olefinic oxindole frameworks were evaluated. The reaction proceeded smoothly with methyleneindolinones containing electron-donating groups, obtaining the **3ab-ad** cycloadducts in good yields, with excellent diastereoselectivities and enantiomeric excesses regardless of the (*Z*) or (*E*) isomer of the olefinic oxindole used. *N*-unsubstituted olefinic oxindoles bearing electron-withdrawing groups on the aromatic ring were also well-tolerated, affording **3ae-ag** cycloadducts in moderate to good yields with excellent diastereoselectivities, although the enantiomeric excesses were higher for the 6-substituted methyleneindolinone than for the 5-substituted one. The methyl- and *tert*-butyl-substituted ester methyleneindolines **2i** afforded the corresponding products **3ah-ai** in good yields and excellent diastereo- and enantioselectivities. In addition, the effect of substituents at the *N*-position of methyleneindolinone was also evaluated, with *N*-methyl and *N*-benzyl substrates yielding products **3aj-am** in moderate to high yields and diastereoselectivities, albeit with lower enantioselectivities compared to the *N*-unsubstituted olefinic oxindoles, which can be attributed to the increased steric hindrance. On the other hand, 2,5-dienone **1b**, bearing 5′,5′-dimethyl groups, predominantly afforded the other diastereoisomer **3ba’’** as the major spirocyclic adduct with moderate yield and diastereomeric ratio and high enantiomeric excess. The synthetic potential of this approach was demonstrated by performing the model reaction on a 1 mmol scale for the preparation of **3aa** (75%, 95:5 dr, 91% ee). The adduct **3aa** was crystallized, and their absolute (1*S*,1′*R*,6′*S*)-configuration was unambiguously established by XRD analysis, assuming the same absolute configuration for the other products **3ab-am**. The absolute (1*R*,1′*R*,6′*S*)-configuration of the other diastereoisomer **3ba’’** was determined by EDC (see SI).

**Scheme 2:**
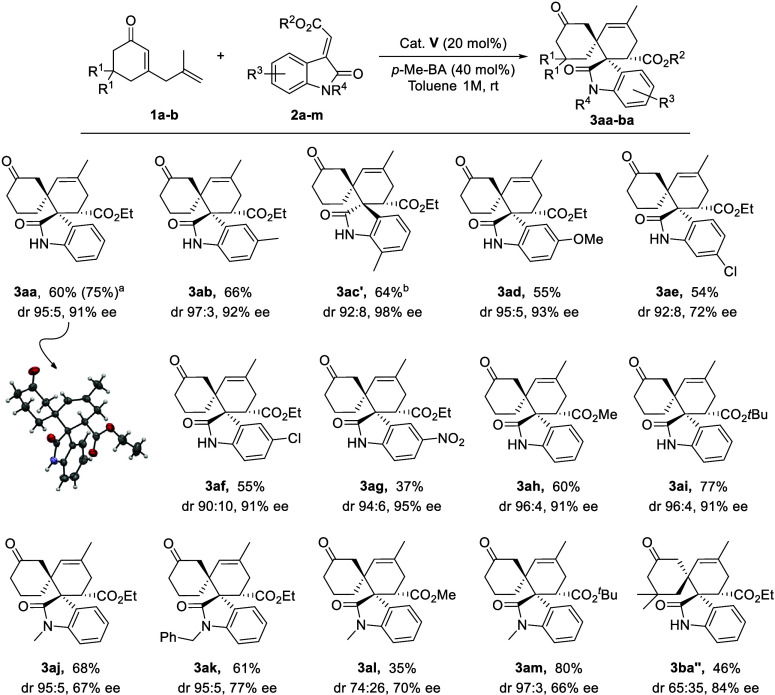
Scope of Enantioselective Cycloadditions between 2,5-Dienone **1a** and Methyleneindolinones **2** ^a^ ^,^ ^b^ ^,^ ^c^

a Performed at a 1 mmol scale.
b The reaction was performed with methyleneindolinone (*Z*)-**2c**. ^*c*^.
c Isolated yields.

In addition to the ester-substituted methyleneindoline **2**, we examined enantioselective β,ε-regioselective [4 + 2] cycloadditions using other olefinic oxindoles bearing various substituents instead of an ester group (Scheme 3). Substrates in which the isatylidene contains a ketone at the terminal position of the C=C double bond proved suitable, yielding the desired products **5aa-ac** in moderate to good yields, high diastereomeric ratios, and good to high ee under conditions. It is worth mentioning that, as observed in the previous cases, a lower ee was obtained by using the *N*-methyl-substituted substrate. Unfortunately, the phenyl-substituted methyleneindolinone did not give the desired product **5ad**, probably due to its relatively low reactivity.

**Scheme 3:**
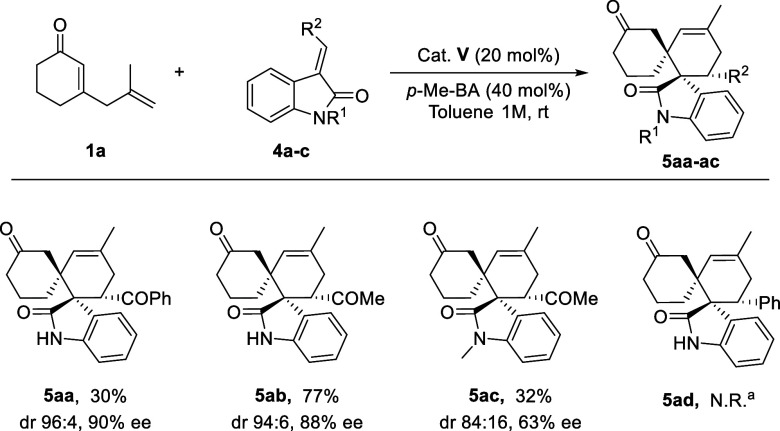
Scope of Enantioselective Cycloadditions between 2,5-Dienone **1a** and Olefinic Oxindoles **4** ^a^

a No reaction occurred.

Based on computational studies performed in our previous work and the proposed mechanistic models, a plausible transition-state model can be postulated to rationalize the high stereoselectivity observed in this [4 + 2] cycloaddition (Scheme 4). This arrangement leads to the selective formation of the observed diastereo- and enantiomerically enriched dispirocyclohexane oxindole products.

**Scheme 4:**
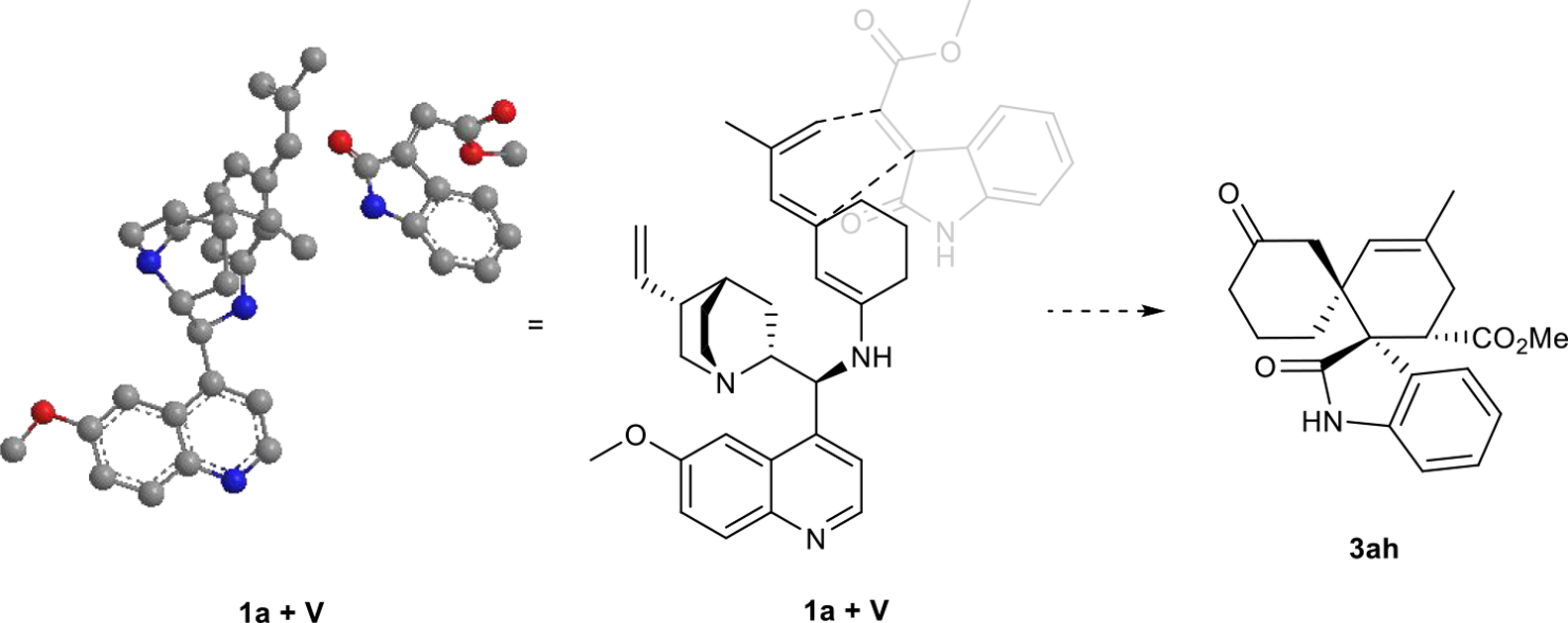
Proposed Transition-State Model

Finally, building on our previous studies, spirocyclic structures containing carbonyl and double-bond motifs have been shown to undergo efficient transformations into valuable derivatives. For example, the carbonyl group can undergo a Horner–Wadsworth–Emmons reaction, and the double bond can be hydrogenated using Raney nickel to afford the corresponding spirocyclic alcohol, as reported in our earlier work.^14^ To further explore the synthetic potential of these structures bearing an ester group, both the ester and carbonyl were reduced with lithium aluminum hydride to give the corresponding diol with a diastereomeric ratio of 70:30 in moderate yield (Scheme 5). Moreover, to demonstrate that *N*-substituted spirocycles could be obtained with higher enantiomeric excess, the protection of the *N*-unsubstituted adduct **3aa** was performed using benzyl bromide and sodium borohydride, affording the corresponding adduct **3ak** in good yield with minimal loss of enantioselectivity (Scheme 5).

**Scheme 5:**
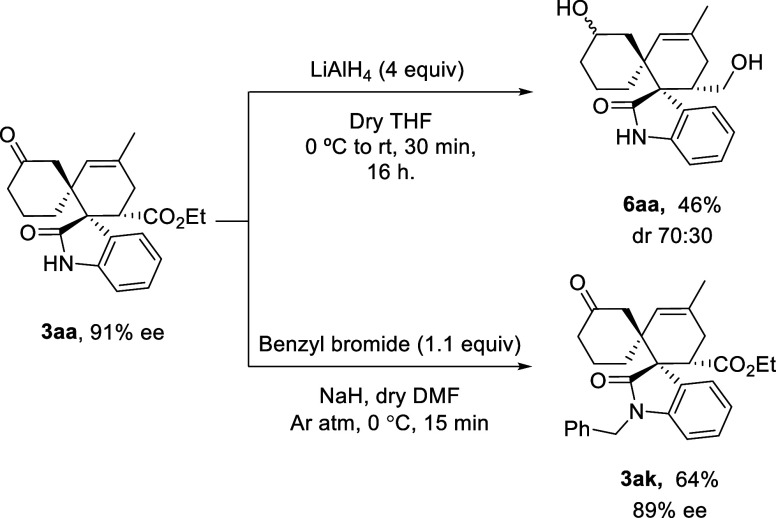
Synthetic Transformations of Adduct **3aa**

## Conclusions

In conclusion, a highly diastereo- and enantioselective organocatalytic synthesis of six-membered ring dispirocyclohexane oxindole derivatives with adjacent spirocyclic centers via [4 + 2] cycloadditions has been successfully developed. The application of a cinchonidine-based primary amine as a catalyst and *p*-methylbenzoic acid as a cocatalyst has facilitated access to diverse enantioenriched dispirocyclohexane oxindoles. The substituent effects were found to be significant for the reaction. Specifically, substituents at the *N*-position were critical for enantioselectivity, with *N*-unsubstituted olefinic oxindoles being the most effective. The *N*-substituted spirocycles could be obtained with high enantiomeric excess by protecting the *N*-unsubstituted adducts. Additionally, the presence of an electron-withdrawing group as a terminal substituent on the C=C bond is essential for reactivity.

Beyond its synthetic utility, this methodology provides access to structurally complex dispirooxindole scaffolds, which are highly valuable in medicinal chemistry. The introduction of contiguous spirocyclic centers into oxindole frameworks increases the three-dimensionality and molecular rigidity, features that are known to enhance biological activity and drug-like properties. Therefore, the products obtained via this enantioselective [4 + 2] cycloaddition approach may serve as versatile intermediates for the preparation of biologically active molecules, natural product analogs, and pharmacologically relevant compounds, highlighting the potential of this methodology to contribute to drug discovery and chemical biology.

## Supplementary Material



## Data Availability

The data underlying this study are available in the published article and its Supporting Information.

## Notes

Experimental procedures, characterization data, NMR spectra, and HPLC spectra (PDF)

## References

[1] a Patel, G. ; Shah, V. R. ; Nguyen, T. A. ; Deshmukh, K. , Eds. Spirooxindole: Chemistry, Synthesis, Characterization, and Biological Significance; Elsevier: 2024. 10.1016/C2023-0-00729-4. ISBN: 978–0-443–22324–2.

[2] Rottmann M. (2010). Spotted Spiroindolones, a potent compound class for
the treatment of malaria. Science.

[3] Jiang T., Kuhen K. L., Wolff K., Yin H., Bieza K., Caldwell J., Bursulaya B., Wu T. Y. H., He Y. (2006). Design, Synthesis, and Biological
Evaluations of Novel Oxindoles as HIV-1 Non-Nucleoside Reverse Transcriptase
Inhibitors. Part 1. Bioorg. Med. Chem. Lett..

[4] Yoshikawa M., Kamisaki H., Kunitomo J., Oki H., Kokubo H., Suzuki A., Ikemoto T., Nakashima K., Kamiguchi N., Harada A., Kimura H., Taniguchi T. (2015). Design and
Synthesis of a Novel 2-Oxindole Scaffold as a Highly Potent and Brain-Penetrant
Phosphodiesterase 10A Inhibitor. Bioorg. Med.
Chem..

[5] Ali M. A., Ismail R., Choon T. S., Yoon Y. K., Wei A. C., Pandian S., Kumar R. S., Osman H., Manogaran E. (2010). Substituted
Spiro­[2.3′]­Oxindolespiro­[3.2’’]-5,6-Dimethoxy-Indane-1’’-One-Pyrrolidine
Analogue as Inhibitors of Acetylcholinesterase. Bioorg. Med. Chem. Lett..

[6] Barnett D. S., Guy R. K. (2014). Antimalarials in Development in 2014. Chem. Rev..

[7] Ding, Q. ; Liu, J.-J. ; Zhang, Z. WO Patent WO 2007/104714, 2007.

[8] Serradeil-Le Gal C., Lacour C., Valette G., Garcia G., Foulon L., Galindo G., Bankir L., Pouzet B., Guillon G., Barberis C., Chicot D., Jard S., Vilain P., Garcia C., Marty E., Raufaste D., Brossard G., Nisato D., Maffrand J. P., Le Fur G. (1996). Characterization of SR 121463A, a Highly Potent and
Selective, Orally Active Vasopressin V2 Receptor Antagonist. J. Clin. Invest..

[9] Bencivenni G., Wu L.-Y., Mazzanti A., Giannichi B., Pesciaioli F., Song M.-P., Bartoli G., Melchiorre P. (2009). Targeting
Structural and Stereochemical Complexity by Organocascade Catalysis:
Construction of Spirocyclic Oxindoles Having Multiple Stereocenters. Angew. Chem., Int. Ed..

[10] Zhou Z., Feng X., Yin X., Chen Y.-C. (2014). Direct
Remote Asymmetric
Bisvinylogous 1,4-Additions of Cyclic 2,5-Dienones to Nitroalkenes. Org. Lett..

[11] Xiao W., Yang Q.-Q., Chen Z., Ouyang Q., Du W., Chen Y.-C. (2018). Regio- and Diastereodivergent
[4 + 2] Cycloadditions
with Cyclic 2,4-Dienones. Org. Lett..

[12] Feng X., Zhou Z., Yin X., Li R., Chen Y.-C. (2014). Enantioselective
Direct Bisvinylogous 1,6-Additions ofβ-Allyl-2-cyclohexenone
to α,α-Dicyanodienes through Trienamine Catalysis. Eur. J. Org. Chem..

[13] Feng X., Zhou Z., Ma C., Yin X., Li R., Dong L., Chen Y.-C. (2013). Trienamines Derived from Interrupted
Cyclic 2,5-Dienones: Remote δ,ε-C = C Bond Activation
for Asymmetric Inverse-Electron-Demand Aza-Diels–Alder Reaction. Angew. Chem., Int. Ed..

[14] Hidalgo-León R., Alberro N., Cossío F. P., Miguel Sansano J., de Gracia Retamosa M. (2023). Remote Asymmetric Bisvinylogous [4+
2] Cycloaddition Reaction to Synthesize Spirocyclic Frameworks. Adv. Synth. Catal..

