# Supplementary material for: Organocatalytic Enantio- and Diastereoselective Synthesis of Dispirooxindole Derivatives with Adjacent Spirocyclic Centers via [4 + 2] Cycloadditions
Source: J Org Chem. 2026 Apr 6;91(15):5351–6. doi: 10.1021/acs.joc.6c00103 (PMC13097253; doi:10.1021/acs.joc.6c00103)
Supplement: Supplementary file 1 [file jo6c00103_si_001.pdf]

Organocatalytic enantio- and diastereoselective synthesis of dispirooxindole derivatives with adjacent spirocyclic centers via [4+2] cycloadditions

Raquel Hidalgo-León,[a] José Trujillo-Sierra,[a] José Miguel Sansano,*,[a] and María de Gracia Retamosa*,[a]

[a] *Departamento de Química Orgánica, Centro de Innovación en Química Avanzada (ORFEO-CINQA) and Institute of Organic Synthesis. Universidad de Alicante. Ctra. Alicante-San Vicente s/n, 03080-Alicante, Spain.*

Index

1. General remarks. S1

2. Optimization of the reaction conditions. S2

3. General procedure. S5

4. Transformations. S5

Bencylation of 3aa. S5

5. Spirocyclic characterization. S7

6. NMR Spectra for all compounds. S18

7. **X-Ray Diffraction Analysis of 3aa** (CCDC 2428417) S35

8. Electronic Circular Dichroism of 3ba''. S37

1. General remarks.

Unless otherwise noted, reagents and substrates were purchased from commercial suppliers. Substituted ketones **1a**, **1b**, **1c**, were prepared according to literature.[1,2] Catalysts **I**[3], **II**[4], **III**[5], **IV**[6], **V**, **VI**[7], **VII**[8], **VIII**[9], and **IX**[10] were prepared following literature procedures. Oxoindoles **2a-n** and **4a-c** were also prepared according to literature procedures.[11]

Analytical TLC was performed Schleicher & Schuell F1400/LS 254 silica gel plates, and the spots were visualized under UV light (λ = 254 nm). Flash column cromatography was carreid out on column silica gel 60 Å (particle size 40-60 µm). Melting points were determined with a Reichert Thermovar hot plate apparatus and are uncorrected. Optical rotations were measured on a JASCO P-1030 or JASCO DIP-1000 polarimeter with a thermally jacketed 5 cm cell at approximately 25 °C and concentrations (c) are given in g/100 mL. The structurally most important peaks of the IR spectra (recorded using a Nicolet 510 P-FT) are listed, and wavenumbers are given in cm^{-1}.

NMR spectra were obtained using a Bruker AC-300 or AC-400 and were recorded at 300 or 400 MHz for ^{1}H NMR and 75 or 101 MHz for ^{13}C NMR, using $CDC1_3$ as solvent and TMS as internal standard (0.00 ppm) unless otherwise stated. The following abbreviations are used to describe peak patterns where appropriate: s = singlet, d = doublet, t = triplet, q = quartet, dd = doublet of doublets, m = multiplet or unresolved and br s = broad signal. All coupling constants (*J*) are given in Hertz (Hz) and chemical shifts in ppm. ^{13}C {1H} NMR spectra were referenced to $CDC1_3$ at 77.16 ppm.

Low-resolution electron impact (EI) mass spectra were obtained using a Agilent 5977B/MSD by injection or DIP; fragment ions in m/z are given with relative intensities (%) in parentheses. High-resolution mass spectra (HRMS) were measured on an instrument using a quadrupole time-of-flight mass spectrometer (QTOF) and also through the electron impact mode (EI) at 70 eV using a Finnigan VG Platform or a Finnigan MAT 95S. Enantioselectivities were measured by HPLC JASCO using chiral stationary phases. In these experiments the racemic mixtures were analysed in order to establish the enantiomeric parameters of each enantiomer. X-ray crystal structure was determined using a Bruker CCD-Apex.

---

[1] H. Hénon; M. Mauduit; A. Alexakis. *Angew. Chem. Int. Ed*. **2008**, *47*, 9122-9124.

[2] a) G. F. Woods; I. W. Tucker. *J. Am. Chem. Soc*. **1948**, *70*, 2174. b) G. F. Woods; P. H. Griswold; B. H. Armbrecht; D. I. Blumenthal; R. Plapinger. *J. Am. Chem. Soc*. **1949**, *71*, 2028.

[3] Vizcaíno-Milla, P.; Sansano, J. M.; Nájera, C.; Fiser, B.; Gómez-Bengoa, E. *Eur. J. Org. Chem*. **2015**, 2614-2621.

[4] Yu, F.; Sun, X.; Jin, Z.; Wenm S.; Liang, X.; Ye, J. *Chem. Commun*. **2010**, *46*, 4589-4591.

[5] Yu, F.; Jin, Z.; Huang, H.; Ye, T.; Liang, X.; Ye, J. *Organic & Biomolecular Chemistry*. **2010**, *8* (20), 4767-4774.

[6] Acaso-Alegre, C.; Herrera, R. P.; Mangas-Sánchez, J. *Angew. Chem. Int. Ed*. **2022**, *61*, e202209159.

[7] T. Peňaška; V. Palchykov; E. Rakovský; G. Addová; R. Sebesta. *Eur. J. Org. Chem*. **2021**, 1693-1703.

[8] Castelló, L. M.; Nájera, C.; Sansano, J, M.; Larrañaga, O.; Cózar, A.; Cossío, F. P. *Adv. Synth. Catal*. **2014**. *356*, 3186-3870.

[9] Puglisi, A.; Benglia, M.; Annunziata, R.; Rossi, D. *Tetrahedron Asymmetry***. 2008**, *19*, 2258-2264.

[10] Vizcaíno-Milla, P.; Sansano, J. M.; Nájera, C.; Fiser, B.; Gómez-Bengoa, E. *Eur. J. Org. Chem*. **2015**, *13*, 2614-2621.

[11] (a) Shelke, A. M.; Suryavanshi, G. *Org. Biomol. Chem*. **2015**, *13*, 8669-8675. (b) Liu, Y.-Y.; Duan, S.-W.; Zhang, R.; Liu, Y.-H.; Chen, J.-R.; Xiao, W.-J. *Org. Biomol. Chem*. **2016**, *14*, 5224. (c) Sohail, M.; Tanaka, F. *Angew. Chem*. **2021**, *133*, 21426-21430. (d) Chimaladenne, V.; Surapureddi, S. R. K.; Valluru, K. R.; Kampli, A.; Braham, P. K.; Laxmi, S. V. *Synthetic Communications*. **2022**, *52*, 1357-1367. (e) Zhang, D.; Lin, L.; Yang, J.; Liu, X.; Feng, X. *Angew. Chem. Int. Ed*. **2018**, *57*, 12323-12327.

2. Optimization of the reaction conditions.

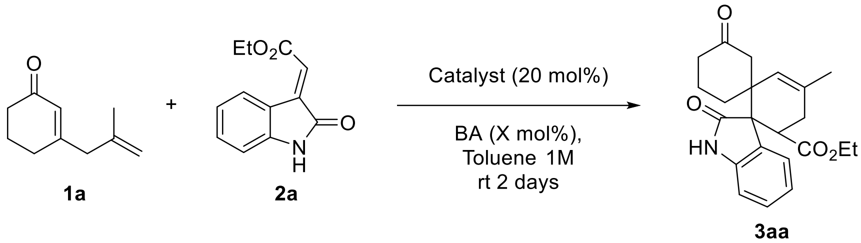

Scheme S1. General reaction to perform the optimization of **3aa**.

To perform the catalyst screening, a reaction mixture of 3-(2-methylallyl)cyclohex-2-en-1-one **1a** (0.2 mmol), ethyl (2*E*)-2-(1,2-dihydro-2-oxo-3H-indol-3-ylidene)acetate **2a** (0.1 mmol), different organocatalysts (0.02 mmol) with benzoic acid (% mmol specified in each case) was stirred at room temperature, and the conversion was determined by ^{1}H-NMR after 2 days. . TLC plates were stained with vanillin.

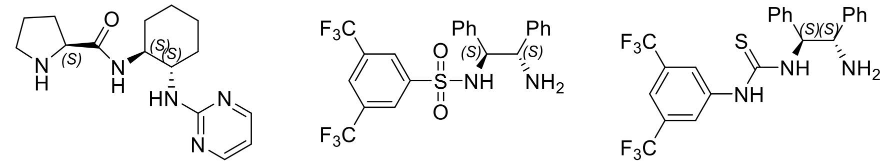

**I**	**II**	**III**
BA (40% mol)<br>full conversion,<br>mixture of products<br>dr 79:21. -10% *ee*	BA (40% mol)<br>77% conversion<br>dr 88:12. 65% *ee*	BA (40% mol)<br>59% conversion<br>dr 86:14. 80% *ee*

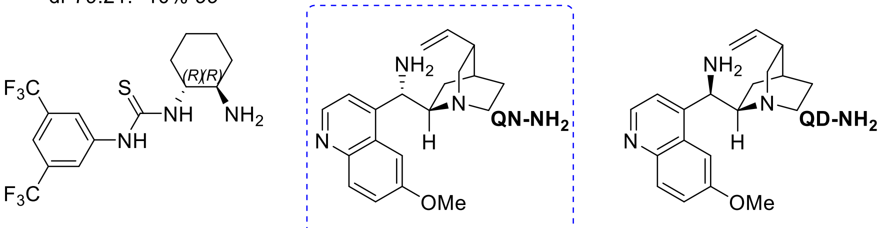

**IV**	**V**	**VI**
BA (10% mol)<br>full conversion<br>dr 91:9. 64% *ee*	BA (40% mol)<br>full conversion<br>dr 95:5. 84% *ee*	BA (40% mol)<br>88% conversion<br>dr 87:13. -84% *ee*

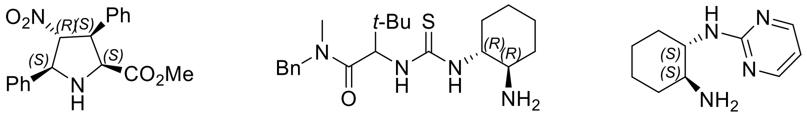

**VII**	**VIII**	**IX**
BA (10% mol)<br>no reaction observed	BA (10% mol)<br>71% conversion<br>dr 90: 10. -76% *ee*	BA (10% mol)<br>79% conversion<br>dr 83:17. 74% *ee*

Scheme S2. Catalyst screening.

Table S1. Reaction between (2*E*)-2-(1,2-dihydro-2-oxo-3H-indol-3-ylidene)acetate **2a** and 3-(2-methylallyl)cyclohex-2-en-1-one **1a** testing a variety of solvents and additives. [a][b]

Entry	Solvent	Acid Additive	Conv (%)[c]	*ee* sp (%)[d]
**1**	Chloroform	BA	>99	77 % *ee*
**2**	Toluene	BA	>99	84 % *ee*
**3**	DCE	BA	>99	78 % *ee*
**4**	TFT	BA	>99	77 % *ee*
**5**	THF	BA	>99	80 % *ee*
**6**	Dioxane	BA	>99	81 *ee*
**7**	H_2O	BA	>99	77 % *ee*
**8[e]**	Toluene	3-OMe-BA	>99	82 % *ee*
**9[e]**	Toluene	SA	>99	76 % *ee*
**10[e]**	Toluene	4-Cl-BA	>99	78 % *ee*
**11[e]**	Toluene	4-Me-BA	>99	85 % *ee*
**12[e]**	Toluene	Et_3N[f]	0	nd
**13[e]**	Toluene	Fenol	0	nd

[a] The reactions were conducted using ketone (0.2 mmol) **1a**, (2*E*)-2-(1,2-dihydro-2-oxo-3H-indol-3-ylidene)acetate (0.1 mmol) **2a** in the presence of catalyst (20 mol %), additives (40 mol %) and solvent (1M). [b] In all cases, the diastereoselectivity was 95:5 or higher. [c] Conversions were measured by ^{1}H-NMR of crude reaction after 2 days. [d] Enantiomeric excesses were measured by HPLC. [e] After 1 day of reaction. [f] In this case, additive was in 20% mol.

Table S2. Reaction between ketone **1a** and (2*E*)-2-(1,2-dihydro-2-oxo-3H-indol-3-ylidene)acetate **2b** catalyzed by QN-NH_2 **V** evaluating different concentrations and equivalents of ketone **1a** and organocatalyst **V**.[a],[b],[c]

Entry	Concentration [M]	Equiv. ketone	Catalyst (%)	Conv. (%)	*ee* sp (%)[d]
**1**	1	2	20	>99	84 % *ee*
**2**	2	2	20	73	86 % *ee*
**3**	0.5	2	20	>99	84 % *ee*
**4**	1	2.5	20	>99	82 % *ee*
**5**	1	1.5	20	>99	85 % *ee*
**6[e]**	1	1.5	20	>99	76 % *ee*
**7[f]**	1	1.5	20	60	89 % *ee*
**8[f]**	1	2	20	72	90 % *ee*
**9[f]**	1	2.5	20	100	91 % *ee*
**10[f]**	1	2	15	53	91 % *ee*

[a] The reactions were conducted using ketone **1a**, (2*E*)-2-(1,2-dihydro-2-oxo-3H-indol-3-ylidene)acetate **2a** (0.1 mmol) in the presence of organocatalyst **V** and *p*-Me-BA (40 mol %) using toluene as solvent. [b] Reactions were monitorized by ^{1}H-NMR after 1 day and allowed to stir at room temperature. [c] In all the cases, the diastereoselectivity was 95:5 or higher. [d] Enantiomeric excesses measured by HPLC. [e] Reaction carried out at 40 °C. [f] Performed at 17 °C.

3. General procedure.

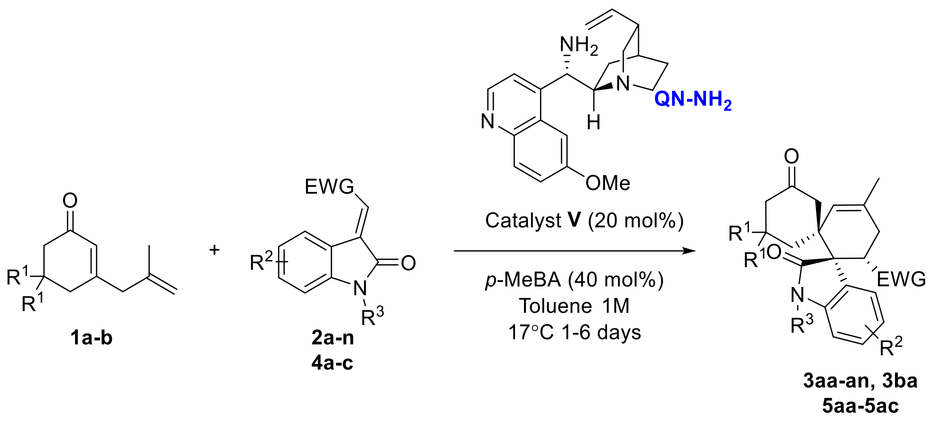

Scheme S3. General procedure to obtain spirocycles **3aa-an**, **3ba** or **5aa-5ac**.

A reaction mixture of dienophile **2a-n** or **5aa-5ac** (0.2 mmol), ketone **1a**-b (0.5 mmol), organocatalyst **V** (0.04 mmol) and *p*-methyl benzoic acid (0.08 mmol) in toluene (200 µL) was stirred at 17 °C in a cryostat and monitored by TLC until the reaction finishes. After this time, the solvent was eliminated under reduced pressure. The crude was washed with saturated $NaHCO_3$, and the organic phase was dryed over anhydrous $MgSO_4$ and concentrated under reduced pressure. The resulting residue was purified by flash chromatography in Hex/EtOAc 4:1 to 1:1 obtaining the desired spiroadducts as white or pale-yellow solids.

4. Transformations.

Bencylation of 3aa.

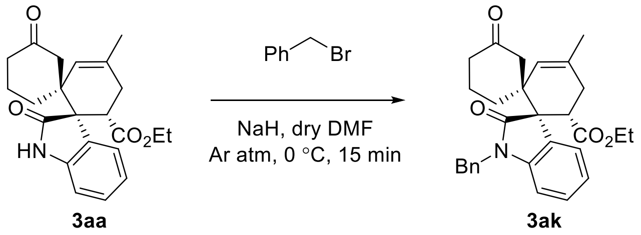

Scheme S4. Formation of **3ak** from **3aa** with benzyl bromide.

In a flame-dried Schlenk, compound **3aa** (0.1 mmol) was dissolved in dry DMF (179 µL) under Ar atmosphere and cooled down to 0 °C in an ice bath. Then, NaH (0.11 mmol) was added and the crude was stirred for 10 minutes. Then, benzyl bromide (0.11 mmol) is additioned and the reaction stirred for a further 15 minutes. The crude was washed with cold brine and extracted x3 times with $CHCl_3$. The combination of organic phases was dried over anhydrous $MgSO_4$ and concentrated under reduced pressure. The resulting residue was purified by flash cromatography in Hex/EtOAc 3:1 to 1:1 to give the desired spiroadduct **3ak** (29 mg, 64% yield) as a pale yellow solid, and 10 mg (28% yield) of the starting material **3aa** can be recovered. With this methodology, we could improve the enantiomeric excess of the final benzylated compound **3ak** from 77% *ee* to 89% *ee*. The chromatogram is given in the characterisation of compound **3ak**.

Reduction of **3aa** to **6aa**.

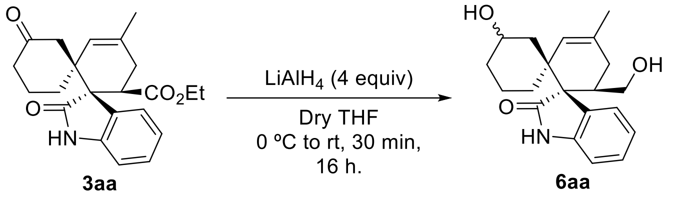

Scheme S5. Reduction of **3aa** with LiAlH4 to form **6aa**.

In an Schlenk flask under Ar atmosphere, $LiAlH_4$ (0.4 mmol) was dissolved in dry THF (200 µL), and the mixture was cooled down to 0 °C. Subsequently, compound **3aa** (0.1 mmol) was dissolved in dry THF (200 µL) and it was added to the reaction mixture dropwise. Then, the mixture was heated to 40 °C during 12h. The resulting reaction crude was quenched with NH_4Cl (3 x 5mL) and Et_2O (3 x 5mL), and solvent was eliminated under reduced pressure. Without further purification, compound **6aa** was obtained as a white solid in 46% yield.

5. Spirocyclic characterization.

Ethyl (1*S*,1'*R*,6'*S*)-4'-methyl-2'',3-dioxodispiro[cyclohexane-1,2'-cyclohexane-1',3''-indolin]-3'-ene-6'-carboxylate, **3aa**.

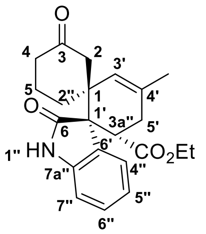

Following the general procedure, starting from ethyl (2*E*)-2-(1,2-dihydro-2-oxo-3*H*-indol-3-ylidene)acetate **2a** (43.4 mg, 0.2 mmol) and 3-(2-methylallyl)cyclohex-2-en-1-one **1a** (79 µL, 0.5 mmol), QN-NH_2 **V** (13 mg, 0.02 mmol), 4-methyl benzoic acid (11 mg, 0.04 mmol) and toluene (200 µL), compound **3aa** was obtained as a pale yellow solid (44 mg, 60%; reaction ran for 1 day). The diastereoselectivity of the reaction was 95:5. After purification, the diastereoselectivity was 95:5. Performed at 1 mmol scale, spirocycle **3aa** was isolated (277 mg, 75%, the reaction ran for 1 day), and the crude maintained the same diastereoselectivity. **Mp**: 170-175 °C. **IR** (neat) ν_{max}: 1736, 1711, 1471, 1180, 1057, 1034, 1009, 752, 694 cm^{-1}. **^{1}H NMR** (400 MHz, $CDCl_3$) δ 8.41 (s, 1H), 7.20 (ddd, J = 7.8, 5.8, 3.1 Hz, 1H), 6.94 – 6.86 (m, 3H), 5.46 (q, J = 1.7 Hz, 1H), 4.00 – 3.84 (m, 2H), 3.54 (dd, J = 11.0, 7.6 Hz, 1H), 3.47 – 3.42 (m, 1H), 2.61 (dd, J = 19.0, 7.6 Hz, 1H), 2.50 (dd, J = 19.3, 10.7 Hz, 1H), 2.41 (dd, J = 13.4, 2.2 Hz, 1H), 2.25 (dd, J = 9.8, 7.2 Hz, 2H), 1.83 (d, J = 1.4 Hz, 3H), 1.78 (dq, J = 9.1, 5.7, 4.4 Hz, 2H), 1.44 (dt, J = 13.7, 8.9 Hz, 1H), 1.15 – 1.07 (m, 1H), 1.04 (t, J = 7.1 Hz, 3H). **^{13}C {1H} NMR** (101 MHz, $CDCl_3$) δ 211.3, 180.5, 172.1, 141.8, 134.2, 130.2, 128.3, 125.5, 124.3, 122.4, 109.7, 61.0, 55.2, 50.7, 46.8, 41.8, 40.7, 30.6, 28.3, 23.3, 20.8, 14.0. **MS** (EI) *m/z*: 367 (M^+, 17%), 135 (32), 150 (48), 172 (68), 217 (40), 218 (100). **HRMS** (ESI): m/z calcd for $C_{22}H_{25}NO_4$ [M^+] 367.1784, found 367.1777. The enantiomeric excess was determined by HPLC using a Chiralpak IB column [*n*-hexanes/2-propanol (90:10)]; flow rate 1 mL/min, λ = 212 nm; τ_{major} = 22.9 min, τ_{minor} = 27.3 min (91% *ee*); $[\alpha]_D^{25}$ = -72.0 (c 0.9, $CHCl_3$).

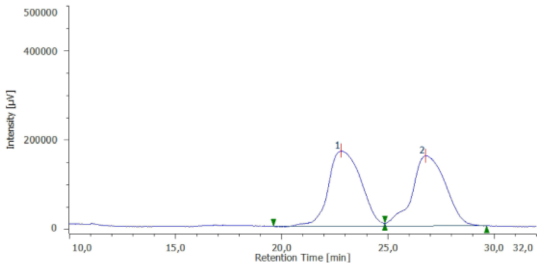

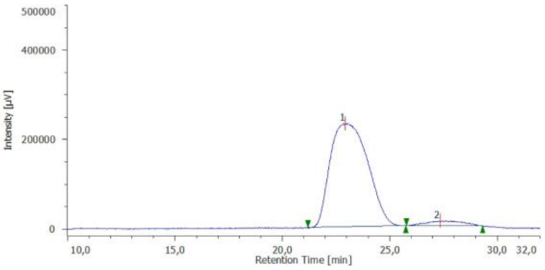

tR [min]	Area [µV·sec]	Height [µV]	Area%	Height%
22,787	17960348	168163	51,034	51,720
26,760	17232335	156979	48,966	48,280

tR [min]	Area [µV·sec]	Height [µV]	Area%	Height%
22,933	28160137	230758	95,479	95,406
27,347	1333275	11112	4,521	4,594

Ethyl (1*S*,1'*R*,6'*S*)-4',5''-dimethyl-2'',3-dioxodispiro[cyclohexane-1,2'-cyclohexane-1',3''-indolin]-3'-ene-6'-carboxylate, **3ab**.

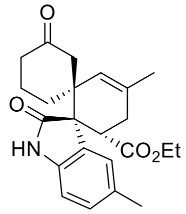

Following the general procedure, starting ethyl (2*E*)-2-(1,2-dihydro-5-methyl-2-oxo-3H-indol-3-ylidene)acetate **2b** (46 mg, 0.2 mmol) and 3-(2-methylallyl)cyclohex-2-en-1-one **1a** (79 µL, 0.5 mmol), QN-NH_2 **V** (13 mg, 0.02 mmol), 4-methyl benzoic acid (11 mg, 0.04 mmol) and toluene (200 µL), compound **3ab** was obtained as a pale yellow solid (51 mg, 66%; reaction ran for 1 day). The diastereoselectivity of the reaction was 97:3. After purification, the diastereoselectivity was 98:2. **Mp**: 59-64 °C. **IR** (neat) ν_{max}: 1701, 1622, 1211, 1192, 1034, 816, 752 cm^{-1}. **^{1}H NMR** (400 MHz, $CDCl_3$) δ 8.59 – 8.39 (m, 1H), 6.97 – 6.90 (m, 1H), 6.72 (d, J = 7.9 Hz, 1H), 6.66 – 6.61 (m, 1H), 5.39 (q, J = 1.7 Hz, 1H), 3.95 – 3.78 (m, 2H), 3.46 (dd, J = 10.9, 7.6 Hz, 1H), 3.39 (d, J = 13.3 Hz, 1H), 2.53 (dd, J = 19.0, 7.6 Hz, 1H), 2.44 (dd, J = 18.6, 10.8 Hz, 1H), 2.34 (d, J = 13.19, 1H), 2.21 (s, 1H), 2.20 (s, 3H), 2.17 (s, 1H), 1.77 (d, J = 1.4 Hz, 3H), 1.76 – 1.68 (m, 2H), 1.37 (ddd, J = 13.5, 10.9, 6.7 Hz, 1H), 1.06 (dd, J = 13.7, 3.0 Hz, 1H), 0.98 (td, J = 7.1, 1.2 Hz, 3H). **^{13}C {1H} NMR** (101 MHz, $CDCl_3$) δ 211.4, 180.6, 172.1, 139.3, 134.0, 131.6, 130.2, 128.6, 126.3, 124.4, 109.4, 60.9, 55.3, 50.8, 46.8, 41.7, 40.7, 30.6, 28.3, 23.2, 21.7, 20.9, 14.0. **MS** (EI) *m/z*: 381 (M^+, 20%), 150 (33), 186 (65), 231 (81), 232 (100). **HRMS** (ESI): m/z calcd for $C_{23}H_{27}NO_4$ [M^+] 381.1940, found 381.1935. The enantiomeric excess was determined by HPLC using a Chiralpak IB column [*n*-

hexanes/2-propanol (90:10)]; flow rate 1 mL/min, λ = 209 nm; τ_{major} = 23.5 min, τ_{minor} = 27.3 min (92% *ee*); **$[\alpha]_D^{25}$** = -81.9 (c 0.9, $CHCl_3$).

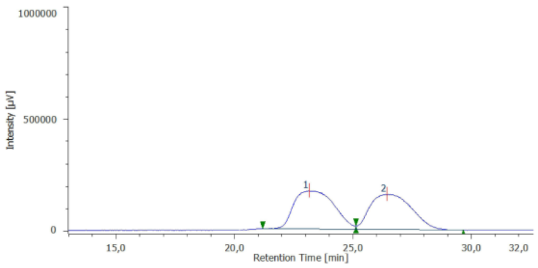

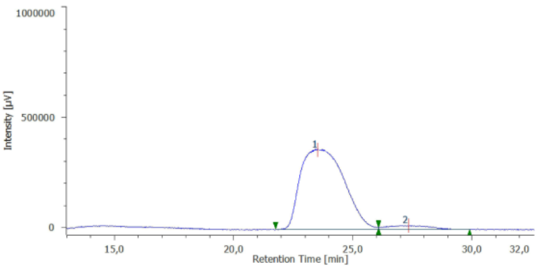

tR [min]	Area [µV·sec]	Height [µV]	Area%	Height%
23,147	20056093	170190	51,110	51,789
26,427	19184787	158429	48,890	48,211

tR [min]	Area [µV·sec]	Height [µV]	Area%	Height%
23,533	46079106	358870	95,905	95,550
27,333	1967510	16714	4,095	4,450

Ethyl (1*S*,1'*S*,6'*S*)-4',7''-dimethyl-2'',3-dioxodispiro[cyclohexane-1,2'-cyclohexane-1',3''-indolin]-3'-ene-6'-carboxylate, **3ac'**.

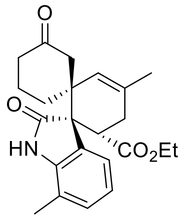

Following the general procedure, starting from ethyl (*Z*)-2-(7-methyl-2-oxoindolin-3-ylidene)acetate **2c** (46 mg, 0.2 mmol) and 3-(2-methylallyl)cyclohex-2-en-1-one **1a** (79 µL, 0.5 mmol), QN-NH_2 **V** (13 mg, 0.02 mmol), 4-methyl benzoic acid (11 mg, 0.04 mmol) and toluene (200 µL), compound **3ac'** was obtained as a pale yellow solid (49 mg, 64%, reaction ran for 4 days). The diastereoselectivity of the reaction was 92:8. After purification, the diastereoselectivity was 93:7. **Mp**: 197-203 °C. **IR** (neat) ν_{max}: 1734, 1709, 1626, 1486, 1211, 1192, 1038, 754 cm^{-1}. **^{1}H NMR** (400 MHz, $CDCl_3$) δ 9.17 (br s, 1H), 7.02 (d, *J* = 7.6 Hz, 1H), 6.84 (t, *J* = 7.6 Hz, 1H), 6.76 (d, *J* = 7.5 Hz, 1H), 5.45 (d, *J* = 1.3 Hz, 1H), 3.88 (dddd, *J* = 17.8, 10.8, 7.1, 3.6 Hz, 2H), 3.52 (dd, *J* = 10.1, 7.4 Hz, 1H), 3.48 (d, *J* = 13.9 Hz, 1H), 2.54 (t, *J* = 8.9 Hz, 2H), 2.41 (d, *J* = 13.3 Hz, 1H), 2.27 (s, 3H), 2.24 (dd, *J* = 12.4, 3.9 Hz, 2H), 1.82 (s, 3H), 1.81 – 1.74 (m, 2H), 1.49 – 1.38 (m, 1H), 1.14 – 1.07 (m, 1H), 0.99 (t, *J* = 9.2, 5.1 Hz, 3H). **^{13}C {1H} NMR** (101 MHz, $CDCl_3$) δ 211.5, 181.3, 172.0, 140.5, 134.1, 129.7 (2C), 124.2, 122.8, 122.3, 118.8, 60.8, 55.6, 50.7, 46.8, 41.7, 40.7, 30.5, 28.3, 23.3, 20.8, 16.7, 13.9. **MS** (EI) *m/z*: 381 (M^+, 19%), 43 (54), 135 (22), 150 (32), 232 (100). **HRMS** (ESI): m/z calcd for $C_{23}H_{27}NO_4$ [M^+] 381.1940, found 381.1942. The enantiomeric excess was determined by HPLC using a Chiralpak IB column [*n*-hexanes/2-propanol (90:10)]; flow rate 1 mL/min, λ = 212 nm; τ_{minor} = 30.0 min, τ_{major} = 16.1 min (98% *ee*); **$[\alpha]_D^{25}$** = -66.1 (c 0.7, $CHCl_3$).

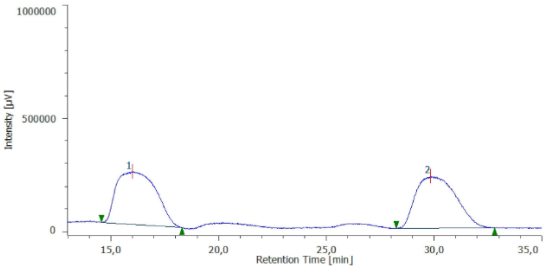

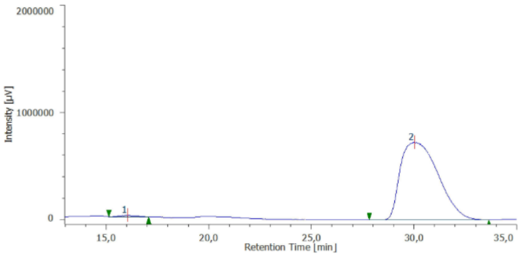

tR [min]	Area [µV·sec]	Height [µV]	Area%	Height%
15,987	29641829	232424	50,080	50,424
29,840	29547466	228514	49,920	49,576

tR [min]	Area [µV·sec]	Height [µV]	Area%	Height%
16,053	794158	12903	0,853	1,758
30,013	92297750	720827	99,147	98,242

Ethyl (1*S*,1'*R*,6'*S*)-5''-methoxy-4'-methyl-2'',3-dioxodispiro[cyclohexane-1,2'-cyclohexane-1',3''-indolin]-3'-ene-6'-carboxylate, **3ad**.

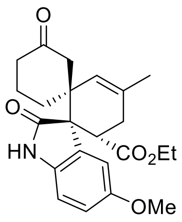

Following the general procedure, starting ethyl (2*E*)-2-(1,2-dihydro-5-methoxy-2-oxo-3H-indol-3-ylidene)acetate **2d** (49 mg, 0.2 mmol) and 3-(2-methylallyl)cyclohex-2-en-1-one **1a** (79 µL, 0.5 mmol), QN-NH_2 **V** (13 mg, 0.02 mmol), 4-methyl benzoic acid (11 mg, 0.04 mmol) and toluene (200 µL), compound **3ad** was obtained as a pale yellow solid (44 mg, 55%; reaction ran for 1 day). The diastereoselectivity of the reaction was 95:5. After purification, the diastereoselectivity was 97:3. **Mp**: 70-75 °C. **IR** (neat) ν_{max}:

1734, 1707, 1485, 1471, 1196, 1055, 1041, 1034, 1022, 1012 cm^{-1}. **^{1}H NMR** (400 MHz, $CDCl_3$) δ 8.56 (s, 1H), 6.80 (d, J = 8.4 Hz, 1H), 6.72 (dd, J = 8.5, 2.5 Hz, 1H), 6.56 (d, J = 2.5 Hz, 1H), 5.45 (q, J = 1.8 Hz, 1H), 4.00 – 3.85 (m, 2H), 3.73 (s, 3H), 3.53 (dd, J = 11.0, 7.6 Hz, 1H), 3.45 (d, J = 13.3 Hz, 1H), 2.60 (dd, J = 19.0, 7.5 Hz, 1H), 2.49 (dd, J = 19.0, 10.9 Hz, 1H), 2.40 (dd, J = 13.5, 2.3 Hz, 1H), 2.25 (dd, J = 9.6, 7.2 Hz, 2H), 1.83 (d, J = 1.4 Hz, 3H), 1.81 – 1.73 (m, 2H), 1.43 (dt, J = 13.6, 8.7 Hz, 1H), 1.13 (dq, J = 14.1, 3.5 Hz, 1H), 1.05 (t, J = 7.1 Hz, 3H). **^{13}C {1H} NMR** (101 MHz, $CDCl_3$) δ 211.3, 180.5, 172.1, 155.4, 135.4, 134.1, 131.6, 124.3, 113.6, 111.7, 109.6, 61.0, 55.6, 55.5, 50.8, 46.8, 41.7, 40.6, 30.5, 28.3, 23.2, 20.9, 14.0. **MS** (EI) *m/z*: 353 (M^+, 13%), 43 (100), 247 (58), 248 (39). **HRMS** (ESI): m/z calcd for $C_{23}H_{27}NO_5$ [M^+] 397.1889, found 397.1884. The enantiomeric excess was determined by HPLC using a Chiralpak IB column [*n*-hexanes/2-propanol (90:10)]; flow rate 1 mL/min, λ = 218 nm; τ_{major} = 30.3 min, τ_{minor} = 36.7 min (93% *ee*); **$[\alpha]_D^{25}$** = -79.4 (c 0.7, $CHCl_3$).

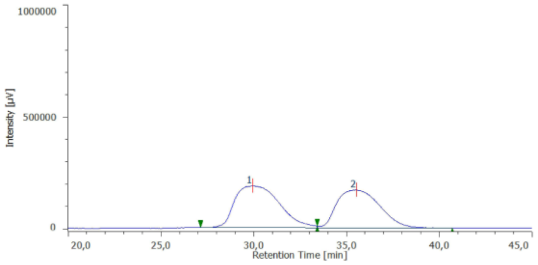

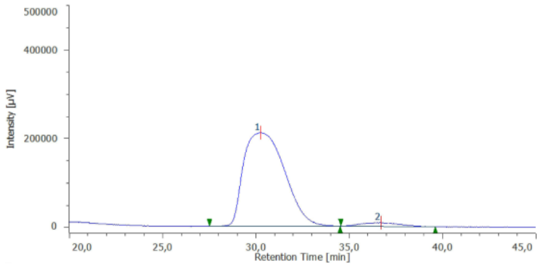

tR [min]	Area [µV·sec]	Height [µV]	Area%	Height%
29,933	31014689	185819	53,494	52,426
35,533	26962812	168619	46,506	47,574

tR [min]	Area [µV·sec]	Height [µV]	Area%	Height%
30,253	32043955	209371	96,466	96,220
36,693	1173947	8226	3,534	3,780

Ethyl (1*S*,1'*R*,6'*S*)-6''-chloro-4'-methyl-2'',3-dioxodispiro[cyclohexane-1,2'-cyclohexane-1',3''-indolin]-3'-ene-6'-carboxylate, **3ae**.

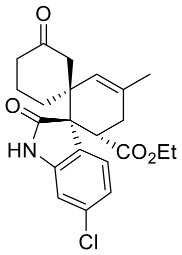

Following the general procedure, starting ethyl (2*E*)-2-(6-chloro-1,2-dihydro-2-oxo-3H-indol-3-ylidene)acetate **2e** (50 mg, 0.2 mmol) and 3-(2-methylallyl)cyclohex-2-en-1-one **1a** (79 µL, 0.5 mmol), QN-NH_2 **V** (13 mg, 0.02 mmol), 4-methyl benzoic acid (11 mg, 0.04 mmol) and toluene (200 µL), compound **3ae** was obtained as a pale yellow solid (45 mg, 54%; reaction ran for 3 days). The diastereoselectivity of the reaction was 92:8. After purification, the diastereoselectivity was 90:10. **Mp**: 97-102 °C. **IR** (neat) ν_{max}: 1724, 1703, 1612, 1238, 1192, 754, 706, 663^{-1}. **^{1}H NMR** (400 MHz, $CDCl_3$) δ 8.70 (s, 1H), 6.94 – 6.88 (m, 2H), 6.82 (d, J = 8.7 Hz, 1H), 5.45 (q, J = 1.7 Hz, 1H), 4.01 – 3.88 (m, 2H), 3.54 (dd, J = 11.2, 7.5 Hz, 1H), 3.39 (d, J = 13.2 Hz, 1H), 2.63 (dd, J = 18.9, 7.6 Hz, 1H), 2.50 – 2.36 (m, 2H), 2.31 – 2.20 (m, 2H), 1.83 (s, 3H), 1.82 – 1.75 (m, 2H), 1.42 (dt, J = 13.6, 8.9 Hz, 1H), 1.15 – 1.11 (m, 1H), 1.09 (t, J = 7.1 Hz, 3H). **^{13}C {1H} NMR** (101 MHz, $CDCl_3$) δ 211.1, 180.6, 172.1, 143.1, 134.2, 134.1, 128.6, 126.3, 124.3, 122.3, 110.4, 61.2, 54.9, 50.6, 46.8, 41.8, 40.6, 30.6, 28.3, 23.3, 20.8, 14.0. **MS** (EI) *m/z*: 401 (M^+, 16%), 135 (54), 150 (100), 206 (60), 252 (72). **HRMS** (ESI): m/z calcd for $C_{22}H_{24}ClNO_4$ [M^+] 401.1394, found 401.1392. The enantiomeric excess was determined by HPLC using a Chiralpak IB column [*n*-hexanes/2-propanol (95:5)]; flow rate 1 mL/min, λ = 219 nm; τ_{major} = 48.2 min, τ_{minor} = 65.1 min (72% *ee*); **$[\alpha]_D^{25}$** = -47.1 (c 0.4, $CHCl_3$).

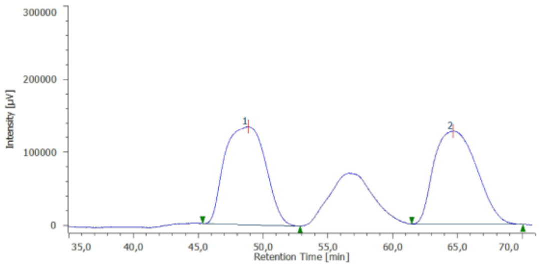

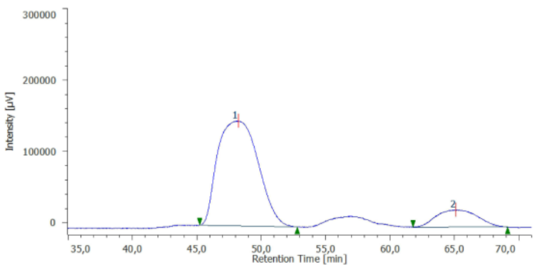

tR [min]	Area [µV·sec]	Height [µV]	Area%	Height%
48,867	29444340	134340	50,179	51,363
64,680	29234310	127211	49,821	48,637

tR [min]	Area [µV·sec]	Height [µV]	Area%	Height%
48,227	32312759	147244	85,780	85,800
65,133	5356718	24370	14,220	14,200

Ethyl (1*S*,1'*R*,6'*S*)-5''-chloro-4'-methyl-2'',3-dioxodispiro[cyclohexane-1,2'-cyclohexane-1',3''-indolin]-3'-ene-6'-carboxylate, **3af**.

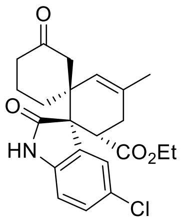

Following the general procedure, starting ethyl (2*E*)-2-(5-chloro-1,2-dihydro-2-oxo-3H-indol-3-ylidene)acetate **2f** (49 mg, 0.2 mmol) and 3-(2-methylallyl)cyclohex-2-en-1-one **1a** (79 µL, 0.5 mmol), QN-NH_2 **V** (13 mg, 0.02 mmol), 4-methyl benzoic acid (11 mg, 0.04 mmol) and toluene (200 µL), compound **3af** was obtained as a pale yellow solid (45 mg, 55%; reaction ran for 1 day). The diastereoselectivity of the reaction was 90:10. After purification, the diastereoselectivity was 97:3. **Mp**: 65-70 °C. **IR** (neat) ν_{max}: 1703, 1475, 1441, 1238, 1260, 1217, 1207, 1192, 818, 752 cm^{-1}. **^{1}H NMR** (400 MHz, $CDCl_3$) δ 8.90 – 8.63 (m, 1H), 7.18 (ddd, J = 8.3, 2.2, 1.1 Hz, 1H), 6.89 – 6.80 (m, 2H), 5.52 – 5.43 (m, 1H), 4.00 – 3.89 (m, 2H), 3.54 (dd, J = 11.2, 7.5 Hz, 1H), 3.39 (d, J = 13.2 Hz, 1H), 2.64 (dd, J = 19.1, 7.4 Hz, 1H), 2.49 – 2.36 (m, 2H), 2.31 – 2.20 (m, 2H), 1.84 (s, 3H), 1.83 – 1.76 (m, 2H), 1.48 – 1.38 (m, 1H), 1.17 – 1.11 (m, 1H), 1.07 (tdd, J = 7.1, 2.8, 1.3 Hz, 3H). **^{13}C {1H} NMR** (101 MHz, $CDCl_3$) δ 210.9, 180.4, 172.0, 140.6, 134.2, 132.0, 128.3, 127.7, 125.8, 124.3, 110.6, 61.1, 55.5, 50.6, 46.8, 41.8, 40.6, 30.5, 28.3, 23.2, 20.8, 14.0. **MS** (EI) *m/z*: 401 (M^+, 19%),135 (51), 150 (100), 206 (61), 251 (61), 252 (95), 253 (33), 254 (33). **HRMS** (ESI): m/z calcd for $C_{22}H_{24}ClNO_4$ [M^+] 401.1394, found 401.1396. The enantiomeric excess was determined by HPLC using a Chiralpak IA column [*n*-hexanes/2-propanol (90:10)]; flow rate 1 mL/min, λ = 215 nm; τ_{major} = 20.5 min, τ_{minor} = 33.9 min (91% *ee*); **$[\alpha]_D^{25}$** = -70.9 (c 0.9, $CHCl_3$).

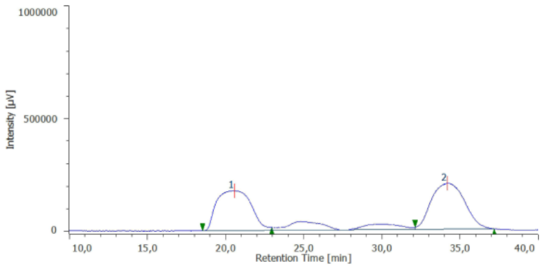

tR [min]	Area [µV·sec]	Height [µV]	Area%	Height%
20,560	27333291	177302	47,470	46,627
34,173	30247013	202958	52,530	53,373

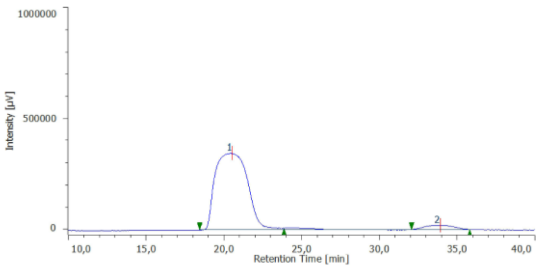

tR [min]	Area [µV·sec]	Height [µV]	Area%	Height%
20,547	50088147	345842	95,403	94,717
33,920	2413536	19291	4,597	5,283

Ethyl (1*S*,1'*R*,6'*S*)-4'-methyl-5''-nitro-2'',3-dioxodispiro[cyclohexane-1,2'-cyclohexane-1',3''-indolin]-3'-ene-6'-carboxylate, **3ag**.

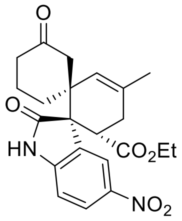

Following the general procedure, starting ethyl (2*E*)-2-(1,2-dihydro-5-nitro-2-oxo-3H-indol-3-ylidene)acetate **2g** (49 mg, 0.2 mmol) and 3-(2-methylallyl)cyclohex-2-en-1-one **1a** (79 µL, 0.5 mmol), QN-NH_2 **V** (13 mg, 0.02 mmol), 4-methyl benzoic acid (11 mg, 0.04 mmol) and toluene (200 µL), compound **3ag** was obtained as a white solid (30 mg, 37%; reaction ran for 3 days). The diastereoselectivity of the reaction was 94:6. After purification, the diastereoselectivity was 97:3. **Mp**: 218-223 °C. **IR** (neat) ν_{max}: 1712, 1703, 1699, 1614, 1471, 1242, 1199, 1059, 1032, 748, 694 cm^{-1}. **^{1}H NMR** (400 MHz, $CDCl_3$) δ 7.95 (s, 1H), 7.21 (dd, J = 8.1, 1.1 Hz, 1H), 6.90 (td, J = 7.8, 1.0 Hz, 1H), 6.82 (d, J = 7.6 Hz, 1H), 5.45 (q, J = 1.7 Hz, 1H), 4.03 – 3.86 (m, 2H), 3.54 (dd, J = 11.1, 7.6 Hz, 1H), 3.39 (d, J = 13.2 Hz, 1H), 2.62 (dd, J = 19.0, 7.7 Hz, 1H), 2.52 – 2.43 (m, 1H), 2.43 – 2.36 (m, 1H), 2.29 – 2.21 (m, 2H), 1.83 (s, 3H), 1.82 – 1.77 (m, 2H), 1.44 (ddd, J = 13.4, 10.4, 7.2 Hz, 1H), 1.12 (d, J = 3.0 Hz, 1H), 1.06 (td, J = 7.2, 1.1 Hz, 3H). **^{13}C {1H} NMR** (101 MHz, $CDCl_3$) δ 210.9, 179.1, 171.9, 139.4, 134.3, 131.6, 128.3, 124.1, 123.7, 123.3, 114.9, 61.2, 56.4, 50.5, 46.9, 41.9, 40.6, 30.5, 28.3, 23.3, 20.8, 13.9. **MS** (EI) *m/z*: 412 (M^+, <1%), 43 (100), 135 (47), 150 (82), 206 (63), 252 (77). **HRMS** (ESI): m/z calcd for $C_{19}H_{20}N_2O_4$ [MH^+ - CO_2Et] 340.1423, found 340.1414. The enantiomeric excess was determined by HPLC using a Chiralpak IA column [*n*-hexanes/2-propanol (90:10)]; flow rate 1 mL/min, λ = 220 nm; τ_{major} = 14.3 min, τ_{minor} = 42.8 min (95% *ee*); **$[\alpha]_D^{25}$** = -61.7 (c 0.6, $CHCl_3$).

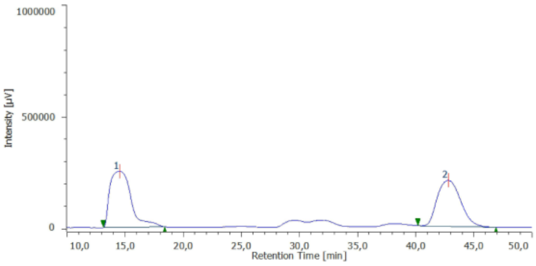

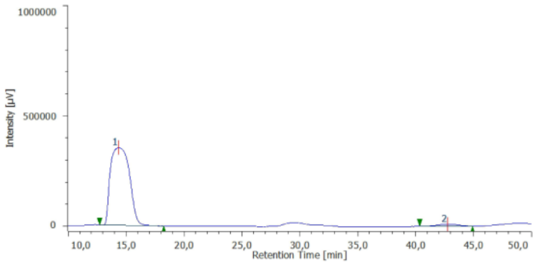

tR [min]	Area [µV·sec]	Height [µV]	Area%	Height%
14,520	31832880	250267	52,632	54,973
42,787	28649555	204990	47,368	45,027

tR [min]	Area [µV·sec]	Height [µV]	Area%	Height%
14,347	40216105	350970	97,255	97,520
42,760	1134952	8926	2,745	2,480

Methyl (1*S*,1'*R*,6'*S*)-4'-methyl-2'',3-dioxodispiro[cyclohexane-1,2'-cyclohexane-1',3''-indolin]-3'-ene-6'-carboxylate, **3ah**.

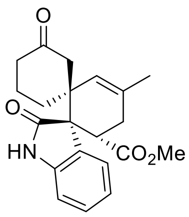

Following the general procedure, starting methyl (2*E*)-2-(1,2-dihydro-2-oxo-3H-indol-3-ylidene)acetate **2h** (41 mg, 0.2 mmol) and 3-(2-methylallyl)cyclohex-2-en-1-one **1a** (79 µL, 0.5 mmol), QN-NH_2 **V** (13 mg, 0.02 mmol), 4-methyl benzoic acid (11 mg, 0.04 mmol) and toluene (200 µL), compound **3ah** was obtained as a white solid (42 mg, 60%; reaction ran for 1 day). The diastereoselectivity of the reaction was 96:4. After purification, the diastereoselectivity was 97:3. **Mp**: 223-228 °C. **IR** (neat) ν_{max}: 1739, 1730, 1712, 1695, 1691, 1618, 1471, 1201, 754, 690 cm^{-1}. **^{1}H NMR** (400 MHz, $CDCl_3$) δ 8.85 (s, 1H), 7.19 (ddd, J = 7.7, 5.6, 3.3 Hz, 1H), 6.97 – 6.86 (m, 3H), 5.46 (q, J = 1.7 Hz, 1H), 3.57 (dd, J = 11.1, 7.5 Hz, 1H), 3.47 (d, J = 3.2 Hz, 1H), 3.45 (s, 3H), 2.65 – 2.56 (m, 1H), 2.54 – 2.45 (m, 1H), 2.42 (dd, J = 13.2, 2.4 Hz, 1H), 2.25 (dd, J = 9.3, 7.2 Hz, 2H), 1.83 (d, J = 1.4 Hz, 3H), 1.81 – 1.74 (m, 2H), 1.50 – 1.38 (m, 1H), 1.10 (dq, J = 13.8, 3.3 Hz, 1H). **^{13}C {1H} NMR** (101 MHz, $CDCl_3$) δ 211.4, 180.7, 172.6, 141.9, 134.0, 130.0, 128.4, 125.4, 124.4, 122.4, 109.8, 55.2, 52.0, 50.7, 46.8, 41.8, 40.6, 30.5, 28.3, 23.2, 20.8. **MS** (EI) *m/z*: 353 (M^+, 20%), 43 (35), 135 (39), 150 (58), 172 (66), 203 (53), 204 (100). **HRMS** (ESI): m/z calcd for $C_{21}H_{23}NO_4$ [M^+] 353.1627, found 353.1624. The enantiomeric excess was determined by HPLC using a Chiralpak IA column [*n*-hexanes/2-propanol (85:15)]; flow rate 1 mL/min, λ = 213 nm; τ_{major} = 14.2 min, τ_{minor} = 27.3 min (91% *ee*); **$[\alpha]_D^{25}$** = -77.2 (c 1.1, $CHCl_3$).

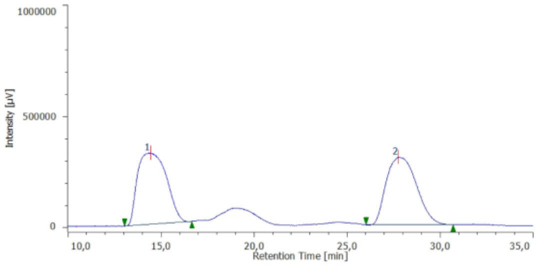

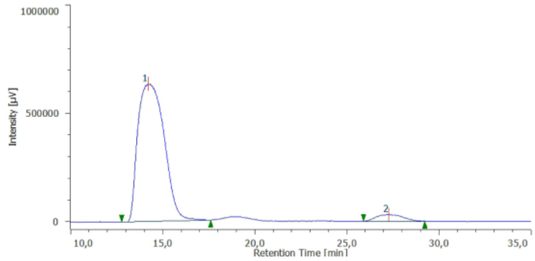

tR [min]	Area [µV·sec]	Height [µV]	Area%	Height%
14,427	34488183	320735	50,089	51,348
27,747	34365672	303899	49,911	48,652

tR [min]	Area [µV·sec]	Height [µV]	Area%	Height%
14,213	62941594	635205	95,261	95,345
27,280	3131477	31013	4,739	4,655

Tert-butyl (1*S*,1'*R*,6'*S*)-4'-methyl-2'',3-dioxodispiro[cyclohexane-1,2'-cyclohexane-1',3''-indolin]-3'-ene-6'-carboxylate, **3ai**.

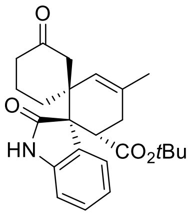

Following the general procedure, starting 1,1-Dimethylethyl (2*E*)-2-(1,2-dihydro-2-oxo-3H-indol-3-ylidene)acetate **2i** (49 mg, 0.2 mmol) and 3-(2-methylallyl)cyclohex-2-en-1-one **1a** (79 µL, 0.5 mmol), QN-NH_2 **V** (13 mg, 0.02 mmol), 4-methyl benzoic acid (11 mg, 0.04 mmol) and toluene (200 µL), compound **3ai** was obtained as a pale yellow solid (61 mg, 77%; reaction ran for 2 days). The diastereoselectivity of the reaction was 96:4. After purification, the diastereoselectivity was 95:5. **Mp**: 75-80 °C. **IR** (neat) ν_{max}: 1702, 1618, 1471, 1236, 1153, 1051, 984, 748 cm^{-1}. **^{1}H NMR** (400 MHz, $CDCl_3$) δ 8.53 (s, 1H), 7.20 (dddd, J = 7.4, 6.2, 2.7, 1.0 Hz, 1H), 6.95 – 6.87 (m, 3H), 5.43 (q, J = 1.7 Hz, 1H), 3.50 – 3.41 (m, 2H), 2.57 – 2.50 (m, 2H), 2.39 (dt, J = 13.3, 2.0 Hz, 1H), 2.28 – 2.21 (m, 2H), 1.84 – 1.81

(m, 3H), 1.77 (dd, J = 8.7, 4.4 Hz, 2H), 1.52 – 1.39 (m, 1H), 1.17 (d, J = 1.4 Hz, 9H), 1.13 – 1.05 (m, 2H). **^{13}C {1H} NMR** (101 MHz, $CDCl_3$) δ 211.4, 180.6, 171.1, 141.6, 134.6, 130.3, 128.3, 125.6, 124.0, 122.4, 109.6, 81.5, 55.5, 50.5, 46.9, 42.3, 40.7, 30.4, 28.4, 27.7, 23.3, 20.9. **MS** (EI) *m/z*: 395 (M^+, 4%), 135 (35), 150 (100), 190 (53), 339 (35). **HRMS** (ESI): m/z calcd for $C_{20}H_{20}NO_4$ [M^+-*t*Bu] 339.1741, found 339.1456. The enantiomeric excess was determined by HPLC using a Chiralpak IA column [*n*-hexanes/2-propanol (90:10)]; flow rate 1 mL/min, λ = 210 nm; τ_{major} = 13.8 min, τ_{minor} = 30.9 min (91% *ee*); **$[\alpha]_D^{25}$** = -72.0 (c 0.2, $CHCl_3$).

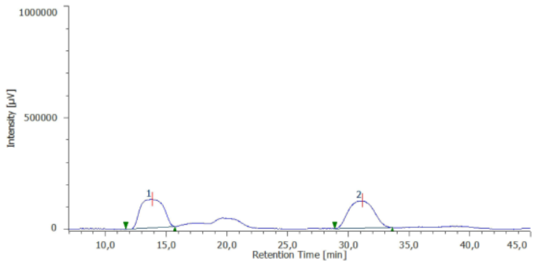

tR [min]	Area [µV·sec]	Height [µV]	Area%	Height%
13,840	16970163	127367	48,798	50,724
31,133	17805867	123728	51,202	49,276

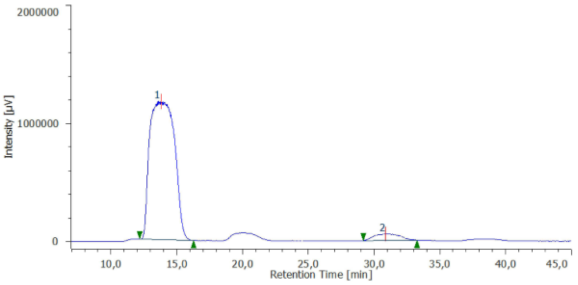

tR [min]	Area [µV·sec]	Height [µV]	Area%	Height%
13,787	153290343	1172087	95,293	95,635
30,867	7571731	53502	4,707	4,365

Ethyl (1*S*,1'*R*,6'*S*)-1'',4'-dimethyl-2'',3-dioxodispiro[cyclohexane-1,2'-cyclohexane-1',3''-indolin]-3'-ene-6'-carboxylate, **3aj**.

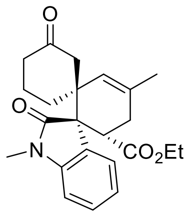

Following the general procedure, starting ethyl (2*E*)-2-(1,2-dihydro-1-methyl-2-oxo-3H-indol-3-ylidene)acetate **2j** (46 mg, 0.2 mmol) and 3-(2-methylallyl)cyclohex-2-en-1-one **1a** (79 µL, 0.5 mmol), QN-NH_2 **V** (13 mg, 0.02 mmol), 4-methyl benzoic acid (11 mg, 0.04 mmol) and toluene (200 µL), compound **3aj** was obtained as a pale yellow solid (51 mg, 68%; reaction ran for 2 days). The diastereoselectivity of the reaction was 95:5. After purification, the diastereoselectivity was 90:10. **Mp**: 53-58 °C. **IR** (neat) ν_{max}: 1697, 1610, 1469, 1373, 1350, 1236, 1196, 1028, 752 cm^{-1}. **^{1}H NMR** (400 MHz, $CDCl_3$) δ 7.31 – 7.27 (m, 1H), 6.97 – 6.94 (m, 2H), 6.85 (dt, J = 7.8, 0.9 Hz, 1H), 5.46 (q, J = 1.7 Hz, 1H), 3.87 (p, J = 7.1 Hz, 2H), 3.54 (dd, J = 10.9, 7.7 Hz, 1H), 3.49 (d, J = 13.3 Hz, 1H), 3.27 (s, 3H), 2.60 (dd, J = 19.0, 7.6 Hz, 1H), 2.54 – 2.45 (m, 1H), 2.43 – 2.38 (m, 1H), 2.27 – 2.21 (m, 2H), 1.83 (s, 3H), 1.78 – 1.71 (m, 1H), 1.29 – 1.22 (m, 1H), 1.09 – 1.05 (m, 1H), 1.01 (t, J = 7.1 Hz, 3H). **^{13}C {1H} NMR** (101 MHz, $CDCl_3$) δ 211.3, 178.3, 172.0, 144.7, 134.2, 129.7, 128.4, 125.1, 124.5, 122.4, 107.9, 60.8, 54.7, 50.8, 46.9, 41.9, 40.6, 30.7, 28.3, 26.6, 23.3, 20.9, 14.0. **MS** (EI) *m/z*: 381 (M^+, 15%), 147 (21), 186 (62), 231 (47), 232 (100). **HRMS** (ESI): m/z calcd for $C_{23}H_{27}NO_4$ [M^+] 381.1940, found 381.1940. The enantiomeric excess was determined by HPLC using a Chiralpak IA column [*n*-hexanes/2-propanol (90:10)]; flow rate 1 mL/min, λ = 254 nm; τ_{major} = 17.6 min, τ_{minor} = 38.0 min (67% *ee*); **$[\alpha]_D^{25}$** = -44.4 (c 0.8, $CHCl_3$).

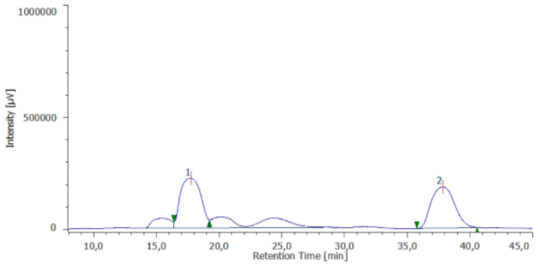

tR [min]	Area [µV·sec]	Height [µV]	Area%	Height%
17,787	25414366	223130	51,617	54,657
37,840	23822109	185107	48,383	45,343

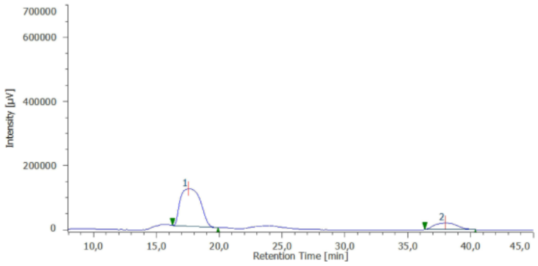

tR [min]	Area [µV·sec]	Height [µV]	Area%	Height%
17,560	13054481	116885	83,637	85,145
37,973	2554079	20393	16,363	14,855

Ethyl (1*S*,1'*R*,6'*S*)-1''-benzyl-4'-methyl-2'',3-dioxodispiro[cyclohexane-1,2'-cyclohexane-1',3''-indolin]-3'-ene-6'-carboxylate, **3ak**.

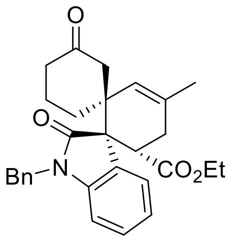

Following the general procedure, starting from ethyl (*E*)-2-(1-benzyl-2-oxoindolin-3-ylidene)acetate **2k** (62 mg, 0.2 mmol) and 3-(2-methylallyl)cyclohex-2-en-1-one **1a** (79 μL, 0.5 mmol), QN-NH_2 **V** (13 mg, 0.02 mmol), 4-methyl benzoic acid (11 mg, 0.04 mmol) and toluene (200 μL), compound **3ak** was obtained as a pale yellow solid (56 mg, 61%, reaction ran for 2 days). The diastereoselectivity of the reaction was 95:5. After purification, the diastereoselectivity was 97:3. **Mp**: 49-54 °C. **IR** (neat) ν_{max}: 1701, 1600, 1466, 1350, 1166, 750, 700 cm^{-1}. **^{1}H NMR** (400 MHz, $CDCl_3$) δ 7.43 – 7.39 (m, 2H), 7.34 (ddd, J = 8.0, 7.0, 1.0 Hz, 2H), 7.30 – 7.27 (m, 1H), 7.18 (ddd, J = 7.8, 7.0, 1.9 Hz, 1H), 6.98 – 6.90 (m, 2H), 6.80 (dt, J = 7.8, 0.8 Hz, 1H), 5.46 (q, J = 1.7 Hz, 1H), 5.15 (d, J = 15.5 Hz, 1H), 4.79 (d, J = 15.6 Hz, 1H), 3.93 – 3.79 (m, 2H), 3.58 (dd, J = 11.0, 7.5 Hz, 1H), 3.51 (d, J = 13.3 Hz, 1H), 2.61 (dd, J = 18.9, 7.5 Hz, 1H), 2.55 – 2.49 (m, 1H), 2.45 (dt, J = 13.2, 2.2 Hz, 1H), 2.27 – 2.15 (m, 2H), 1.84 (s, 3H), 1.79 – 1.65 (m, 2H), 1.30 – 1.20 (m, 2H), 1.12 – 1.04 (m, 1H), 0.99 (t, J = 7.1 Hz, 3H). **^{13}C {1H} NMR** (101 MHz, $CDCl_3$) δ 211.2, 178.3, 172.0, 144.1, 136.4, 134.2, 129.7, 128.8 (2C), 128.3, 127.8 (2C), 127.7, 125.2, 124.5, 122.4, 108.9, 60.8, 54.6, 50.8, 47.1, 44.6, 42.0, 40.7, 30.8, 28.5, 23.3, 20.8, 14.0. **MS** (EI) *m/z*: 457 (M^+, 12%), 91 (91), 150 (18), 223 (19), 262 (48), 308 (100). **HRMS** (ESI): m/z calcd for $C_{29}H_{31}NO_4$ [M^+] 457.2253, found 457.2250. The enantiomeric excess was determined by HPLC using a Chiralpak IA column [*n*-hexanes/2-propanol (70:30)]; flow rate 1 mL/min, λ = 212 nm; τ_{major} = 9.6 min, τ_{minor} = 26.9 min (77% *ee*); **[α]$_D^{25}$** = -52.1 (c 0.7, $CHCl_3$).

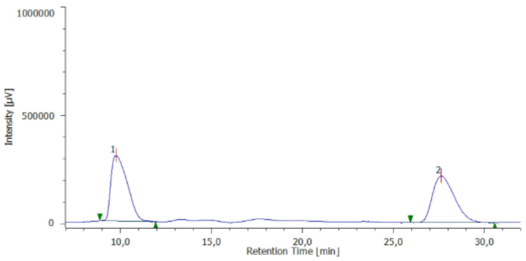

tR [min]	Area [μV·sec]	Height [μV]	Area%	Height%
9,773	18648025	300173	50,958	58,296
27,640	17946693	214743	49,042	41,704

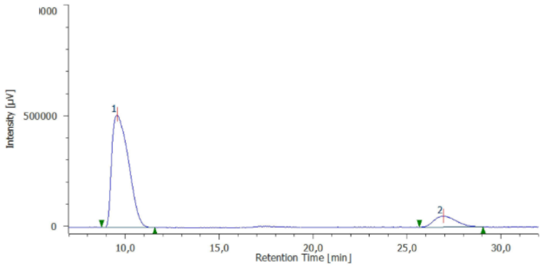

tR [min]	Area [μV·sec]	Height [μV]	Area%	Height%
9,573	31126054	509120	88,522	91,127
26,933	4035818	49572	11,478	8,873

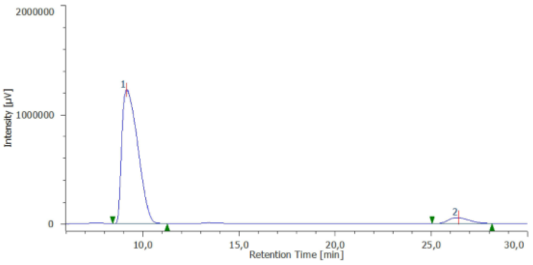

tR [min]	Area [μV·sec]	Height [μV]	Area%	Height%
9,147	70476211	1221200	94,445	95,797
26,400	4144968	53580	5,555	4,203

Methyl (1*S*,1'*R*,6'*S*)-1'',4'-dimethyl-2'',3-dioxodispiro[cyclohexane-1,2'-cyclohexane-1',3''-indolin]-3'-ene-6'-carboxylate, **3al**.

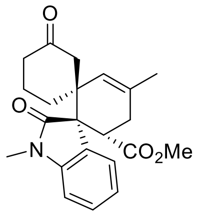

Following the general procedure, starting methyl (2*E*)-2-(1,2-dihydro-1-methyl-2-oxo-3H-indol-3-ylidene)acetate **2l** (41 mg, 0.2 mmol) and 3-(2-methylallyl)cyclohex-2-en-1-one **1a** (79 μL, 0.5 mmol), QN-NH_2 **V** (13 mg, 0.02 mmol), 4-methyl benzoic acid (11 mg, 0.04 mmol) and toluene (200 μL), compound **3al** was obtained as a white solid (25 mg, 35%; reaction ran for 1 day). The diastereoselectivity of the reaction was 74:26. After purification, the diastereoselectivity was 86:14. **Mp**: 122-127 °C. **IR** (neat) ν_{max}: 1790, 1722, 1703, 1608, 1469, 1350, 1236, 1207, 1053, 1030, 746 cm^{-1}. **^{1}H NMR**

(400 MHz, $CDCl_3$) δ 7.31 – 7.27 (m, 1H), 6.98 – 6.92 (m, 2H), 6.87 (dt, J = 7.8, 0.8 Hz, 1H), 5.47 (q, J = 1.7 Hz, 1H), 3.56 (dd, J = 11.1, 7.5 Hz, 1H), 3.49 (d, J = 13.2 Hz, 1H), 3.45 (s, 3H), 3.28 (s, 3H), 2.60 (dd, J = 19.1, 7.3 Hz, 1H), 2.52 – 2.46 (m, 1H), 2.42 (dt, J = 13.2, 2.1 Hz, 1H), 2.27 – 2.21 (m, 2H), 1.83 (s, 3H), 1.80 – 1.72 (m, 2H), 1.30 – 1.19 (m, 1H), 1.10 – 1.02 (m, 1H). **^{13}C {1H} NMR** (101 MHz, $CDCl_3$) δ 211.3, 178.4, 172.4, 144.8, 134.1, 129.6, 128.5, 125.1, 124.6, 122.5, 108.0, 54.6, 52.0, 50.8, 46.8, 41.9, 40.6, 30.7, 28.4, 26.7, 23.3, 20.9. **MS** (EI) *m/z*: 367 (M^+, 16%), 147 (24), 150 (20), 186 (65), 217 (64), 218 (100). **HRMS** (ESI): m/z calcd for $C_{22}H_{25}NO_4$ [M^+] 367.1784, found 367.1779. The enantiomeric excess was determined by HPLC using a Chiralpak IB column [*n*-hexanes/2-propanol (85:15)]; flow rate 1 mL/min, λ = 260 nm; τ_{major} = 12.5 min, τ_{minor} = 35.2 min (70% *ee*); **$[\alpha]_D^{25}$** = -56.1 (c 0.7, $CHCl_3$).

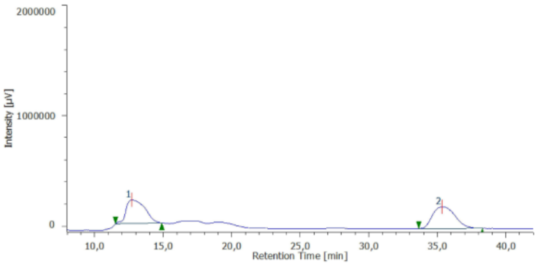

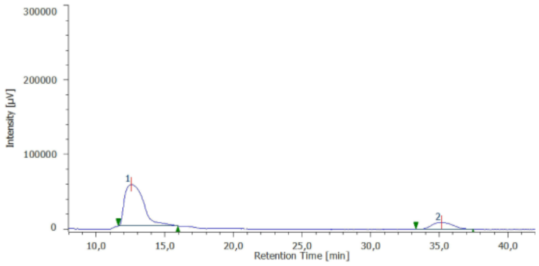

tR [min]	Area [µV·sec]	Height [µV]	Area%	Height%
12,720	20316171	213114	47,624	52,084
35,333	22343353	196056	52,376	47,916

tR [min]	Area [µV·sec]	Height [µV]	Area%	Height%
12,547	5357554	55495	84,803	85,838
35,160	960075	9156	15,197	14,162

Tert-butyl (1*S*,1'*R*,6'*S*)-1'',4'-dimethyl-2'',3-dioxodispiro[cyclohexane-1,2'-cyclohexane-1',3''-indolin]-3'-ene-6'-carboxylate, **3am**.

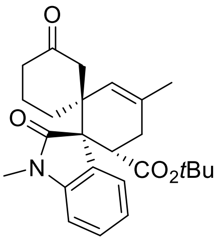

Following the general procedure, starting from *tert*-butyl (E)-2-(1-methyl-2-oxoindolin-3-ylidene)acetate **2m** (52 mg, 0.2 mmol) and 3-(2-methylallyl)cyclohex-2-en-1-one **1a** (79 µL, 0.5 mmol), QN-NH_2 **V** (13 mg, 0.02 mmol), 4-methyl benzoic acid (11 mg, 0.04 mmol) and toluene (200 µL), compound **3am** was obtained as a pale yellow solid (66 mg, 80%, reaction ran for 1 day). The diastereoselectivity of the reaction was 97:3. After purification, the diastereoselectivity was 90:10. **Mp**: 80-85 °C. **IR** (neat) ν_{max}: 1705, 1610, 1471, 1348, 1240, 1153, 1053, 1014, 752 cm^{-1}. **^{1}H NMR** (400 MHz, $CDCl_3$) δ 7.30 – 7.27 (m, 1H), 7.00 – 6.90 (m, 2H), 6.82 (d, *J* = 7.8 Hz, 1H), 5.42 (d, *J* = 1.5 Hz, 1H), 3.51 (d, *J* = 13.3 Hz, 1H), 3.43 (dd, *J* = 10.1, 8.0 Hz, 1H), 3.25 (s, 3H), 2.52 (dd, *J* = 21.4, 8.9 Hz, 2H), 2.39 (d, *J* = 13.4 Hz, 1H), 2.23 (dd, *J* = 9.3, 7.0 Hz, 2H), 2.16 (s, 1H), 1.82 (s, 3H), 1.78 – 1.71 (m, 2H), 1.27 – 1.22 (m, 1H), 1.11 (s, 9H). **^{13}C {1H} NMR** (101 MHz, $CDCl_3$) δ 211.4, 178.0, 171.1, 144.5, 134.6, 129.7, 128.3, 125.3, 124.1, 122.4, 107.7, 81.0, 55.1, 50.5, 47.0, 42.4, 40.6, 30.4, 28.4, 27.7 (3C), 26.5, 23.3, 20.9. **MS** (EI) *m/z*: 409 (M^+, 9%), 135 (19), 150 (48), 186 (33), 204 (100), 336 (9), 353 (18). **HRMS** (ESI): m/z calcd for $C_{22}H_{25}NO_4$ [M^+-C_3H_6] 367.1784, found 367.1782. The enantiomeric excess was determined by HPLC using a Chiralpak IA column [*n*-hexanes/2-propanol (90:10)]; flow rate 1 mL/min, λ = 212 nm; τ_{major} = 12.9 min, τ_{minor} = 21.0min (66% *ee*); **$[\alpha]_D^{25}$** = -19.9 (c 1.1, $CHCl_3$).

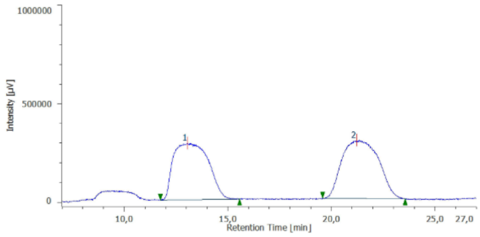

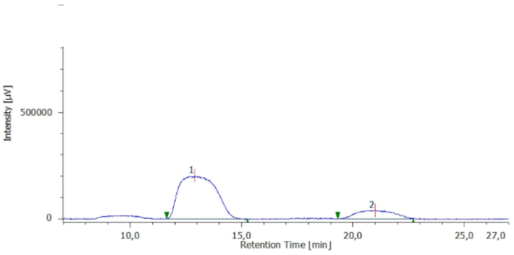

tR [min]	Area [µV·sec]	Height [µV]	Area%	Height%
13,053	33800566	287363	48,126	49,400
21,213	36432778	294348	51,874	50,600

tR [min]	Area [µV·sec]	Height [µV]	Area%	Height%
12,893	23365055	200923	83,046	83,118
20,987	4770001	40811	16,954	16,882

Ethyl 3,3,4'-trimethyl-2'',5-dioxodispiro[cyclohexane-1,2'-cyclohexane-1',3''-indolin]-3'-ene-6'-carboxylate, **3ba''**.

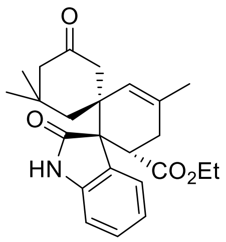

Following the general procedure, starting from ethyl (*E*)-2-(2-oxoindolin-3-ylidene)acetate **2n** (43 mg, 0.2 mmol) and 5,5-dimethyl-3-(2-methylallyl)cyclohex-2-en-1-one **1b** (89 mg, 0.5 mmol), QN-NH_2 **V** (13 mg, 0.02 mmol), 4-methyl benzoic acid (11 mg, 0.04 mmol) and toluene (200 µL), compound **3ba''** was obtained as a yellow oil (36 mg, 46%, reaction ran for 6 days). The diastereoselectivity of the reaction was 35:65. After purification, the diastereoselectivity was 13:87. **IR** (neat) ν_{max}: 1705, 1620, 1470, 1219, 1186, 748, 652, 633, 613 cm^{-1}. **^{1}H NMR** (400 MHz, $CDCl_3$)) δ 8.49 – 8.25 (m, 1H), 7.14 (tdd, J = 7.7, 2.1, 0.8 Hz, 1H), 6.91 – 6.79 (m, 3H), 5.42 (q, J = 1.7 Hz, 1H), 4.00 – 3.83 (m, 2H), 3.77 (dd, J = 10.6, 8.0 Hz, 1H), 2.87 (d, J = 13.9 Hz, 1H), 2.70 – 2.61 (m, 1H), 2.43 (ddd, J = 19.7, 10.6, 1.6 Hz, 1H), 2.20 (d, J = 13.2 Hz, 1H), 2.06 (d, J = 13.1 Hz, 1H), 1.99 (dd, J = 13.4, 1.0 Hz, 1H), 1.81 (d, J = 1.4 Hz, 3H), 1.70 (dd, J = 13.9, 2.1 Hz, 1H), 1.56 (d, J = 13.3 Hz, 1H), 1.10 (s, 3H), 1.03 (t, J = 7.1 Hz, 4H), 0.97 (s, 3H). **^{13}C {1H} NMR** (101 MHz, $CDCl_3$) δ 211.8, 180.4, 172.4, 141.6, 132.0, 129.6, 128.4, 128.3, 125.5, 122.5, 109.6, 60.9, 55.8, 54.1, 46.6, 45.7, 45.3, 42.3, 35.5, 34.3, 30.1, 29.3, 22.8, 14.0. **MS** (EI) *m/z*: 395 (M^+, 12%), 79 (10), 122 (8), 145 (14), 163 (39), 172(65), 178 (51), 189 (10), 218 (100). **HRMS** (ESI): m/z calcd for $C_{24}H_{29}NO_4$ [M^+] 395.2097, found 395.2096. The enantiomeric excess was determined by HPLC using a Chiralpak IA column [*n*-hexanes/2-propanol (90:10)]; flow rate 1 mL/min, λ = 212 nm; τ_{minor} = 20.1 min, τ_{major} = 14.4 min (84% *ee*); **$[\alpha]_D^{25}$** = -64.2 (c 1.0, $CHCl_3$).

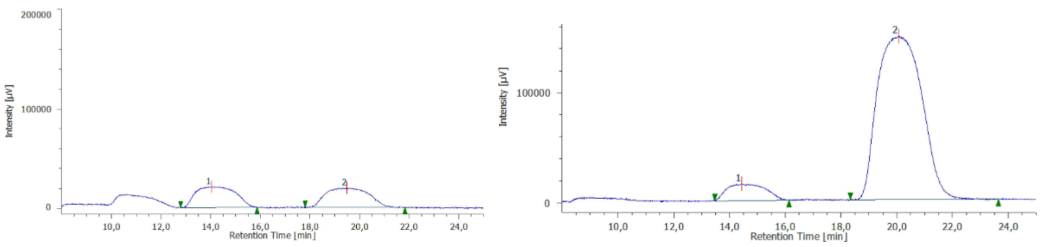

tR [min]	Area [µV·sec]	Height [µV]	Area%	Height%
14,027	2326902	20990	49,795	51,603
19,493	2346105	19686	50,205	48,397

tR [min]	Area [µV·sec]	Height [µV]	Area%	Height%
14,440	1464000	15427	8,183	9,440
20,053	16427756	147988	91,817	90,560

(1*S*,1'*R*,6'*S*)-6'-benzoyl-4'-methyldispiro[cyclohexane-1,2'-cyclohexane-1',3''-indolin]-3'-ene-2'',3-dione, **5aa**.

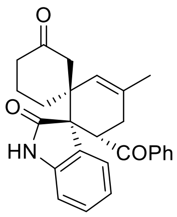

Following the general procedure, starting (3*E*)-1,3-Dihydro-3-(2-oxo-2-phenylethylidene)-2H-indol-2-one **4a** (50 mg, 0.2 mmol) and 3-(2-methylallyl)cyclohex-2-en-1-one **1a** (79 µL, 0.5 mmol), QN-NH_2 **V** (13 mg, 0.02 mmol), 4-methyl benzoic acid (11 mg, 0.04 mmol) and toluene (200 µL), compound **5aa** was obtained as a pale yellow solid (24 mg, 30%; reaction ran for 4 days). The diastereoselectivity of the reaction was 96:4. After purification, the diastereoselectivity was 96:4. **Mp**: 179-184 °C. **IR** (neat) ν_{max}: 1707, 1618, 1469, 1215, 1205, 1198, 752, 667, 640 cm^{-1}. **^{1}H NMR** (400 MHz, $CDCl_3$)) δ 8.27 (s, 1H), 7.84 – 7.78 (m, 2H), 7.56 – 7.49 (m, 1H), 7.41 (dd, J = 8.3, 7.2 Hz, 2H), 7.21 (dddd, J = 8.7, 7.6, 2.1, 0.9 Hz, 1H), 6.95 (qd, J = 7.7, 1.6 Hz, 2H), 6.88 – 6.83 (m, 1H), 5.52 (q, J = 1.7 Hz, 1H), 4.53 (dd, J = 12.2, 6.4 Hz, 1H), 3.45 (d, J = 13.2 Hz, 1H), 2.67 (dd, J = 13.5, 2.4 Hz, 1H), 2.53 (dd, J = 18.5, 6.5 Hz, 1H), 2.42 – 2.34 (m, 1H), 2.29 (t, J = 8.1 Hz, 2H), 1.80 (s, 3H), 1.79 – 1.75 (m, 2H), 1.46 – 1.35 (m, 1H), 1.17 – 1.10 (m, 1H). **^{13}C {1H} NMR** (101 MHz, $CDCl_3$) δ 211.6, 200.3, 180.0, 141.7, 136.5, 134.3, 133.3, 131.1, 128.8 (2C), 128.7 (2C), 128.0, 125.2, 125.0, 122.3, 109.8, 55.4, 51.2, 47.1, 44.3, 40.7, 32.6, 28.3, 23.3, 21.0. **MS** (EI) *m/z*: 399 (M^+, <1%), 43 (78), 150 (31), 161 (74), 172 (41), 187 (30), 188 (100), 294 (64). **HRMS** (ESI): m/z calcd for $C_{19}H_{18}NO_2$ [M^+-COPh] 292.1338, found 292.1334. The enantiomeric excess was determined by HPLC using a Chiralpak IA column [*n*-hexanes/2-propanol (60:40)]; flow rate 1 mL/min, λ = 218 nm; τ_{major} = 7.1 min, τ_{minor} = 14.0 min (90% *ee*); **$[\alpha]_D^{25}$** = -53.0 (c 0.3, $CHCl_3$).

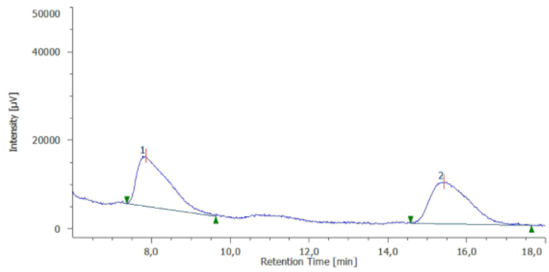

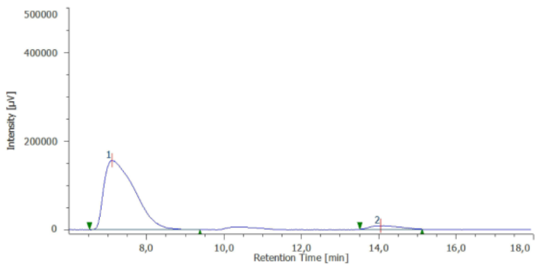

tR [min]	Area [µV·sec]	Height [µV]	Area%	Height%
7,853	631260	11361	49,455	54,510
15,427	645173	9481	50,545	45,490

tR [min]	Area [µV·sec]	Height [µV]	Area%	Height%
7,107	8518547	155678	95,120	94,887
14,040	437002	8388	4,880	5,113

(1*S*,1'*R*,6'*S*)-6'-acetyl-4'-methyldispiro[cyclohexane-1,2'-cyclohexane-1',3''-indolin]-3'-ene-2'',3-dione, **5ab**.

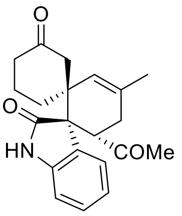

Following the general procedure, starting (3*E*)-1,3-Dihydro-3-(2-oxo-2-phenylethylidene)-2H-indol-2-one **4b** (37 mg, 0.2 mmol) and 3-(2-methylallyl)cyclohex-2-en-1-one **1a** (79 µL, 0.5 mmol), QN-NH_2 **V** (13 mg, 0.02 mmol), 4-methyl benzoic acid (11 mg, 0.04 mmol) and toluene (200 µL), compound **5ab** was obtained as a pale yellow solid (52 mg, 77%; reaction ran for 1 day). The diastereoselectivity of the reaction was 94:6. After purification, the diastereoselectivity was 91:9. **Mp**: 74-79 °C. **IR** (neat) ν_{max}: 1699, 1620, 1470, 1223, 748, 698, 634, 613 cm^{-1}. **^{1}H NMR** (400 MHz, $CDCl_3$) δ 8.11 (s, 1H), 7.13 (td, J = 7.6, 1.3 Hz, 1H), 6.89 – 6.76 (m, 3H), 5.42 (q, J = 1.7 Hz, 1H), 3.54 (dd, J = 12.1, 6.9 Hz, 1H), 3.29 (d, J = 13.2 Hz, 1H), 2.54 (dd, J = 18.0, 7.0 Hz, 1H), 2.40 – 2.34 (m, 1H), 2.34 – 2.24 (m, 1H), 2.22 – 2.14 (m, 2H), 2.00 (s, 3H), 1.79 (d, J = 1.4 Hz, 3H), 1.71 (qd, J = 6.2, 2.6 Hz, 2H), 1.34 (dt, J = 13.7, 8.8 Hz, 1H), 1.05 – 0.97 (m, 1H). **^{13}C {1H} NMR** (101 MHz, $CDCl_3$) δ 211.4, 207.1, 180.5, 141.8, 133.6, 130.4, 128.1, 125.2, 125.1, 122.3, 109.8, 54.9, 50.9, 49.7, 46.8, 40.7, 30.6, 28.7, 28.0, 23.4, 20.9. **MS** (EI) *m/z*: 337 (M^+, <1%), 77 (45), 105 (63), 161 (74), 221 (35), 250 (100), 294 (86). **HRMS** (ESI): m/z calcd for $C_{19}H_{20}NO_2$ [M^+-COMe] 294.1494, found 294.1490. The enantiomeric excess was determined by HPLC using a Chiralpak IA column [*n*-hexanes/2-propanol (60:40)]; flow rate 1 mL/min, λ = 215 nm; τ_{minor} = 8.2 min, τ_{major} = 13.6 min (88% *ee*); **$[\alpha]_D^{25}$** = -84.3 (c 0.9, $CHCl_3$).

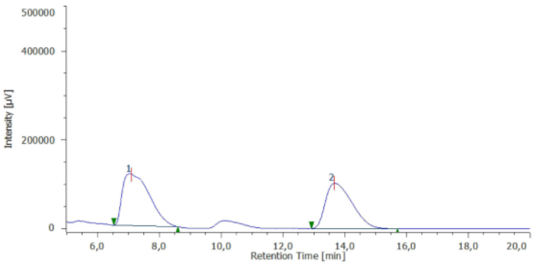

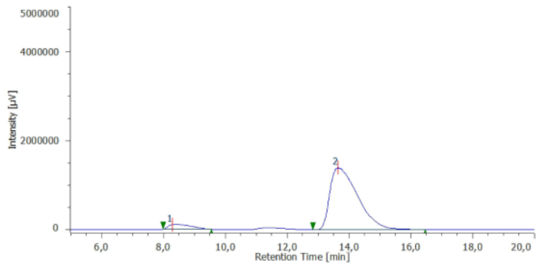

tR [min]	Area [µV·sec]	Height [µV]	Area%	Height%
7,107	7084117	114468	53,782	52,814
13,667	6087795	102271	46,218	47,186

tR [min]	Area [µV·sec]	Height [µV]	Area%	Height%
8,293	5545204	105506	6,000	7,009
13,653	86873494	1399888	94,000	92,991

(1*S*,1'*R*,6'*S*)-6'-acetyl-1'',4'-dimethyldispiro[cyclohexane-1,2'-cyclohexane-1',3''-indolin]-3'-ene-2'',3-dione, **5ac**.

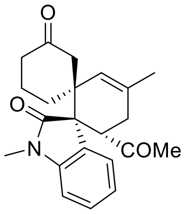

Following the general procedure, starting (*E*)-1-methyl-3-(2-oxopropylidene)indolin-2-one **4c** (37 mg, 0.2 mmol) and 3-(2-methylallyl)cyclohex-2-en-1-one **1a** (79 µL, 0.5 mmol), QN-NH_2 **V** (13 mg, 0.02 mmol), 4-methyl benzoic acid (11 mg, 0.04 mmol) and toluene (200 µL), compound **5ac** was obtained as a light brown solid (22 mg, 32%; reaction ran for 1 day). The diastereoselectivity of the reaction was 84:16. After purification, the diastereoselectivity was 83:17. **Mp**: 123-128 °C. **IR** (neat) ν_{max}: 1697, 1608, 1469, 1352, 1055, 1032, 1015, 748 cm^{-1}. **^{1}H NMR** (400 MHz, $CDCl_3$) δ 7.28 (dd, *J*

= 7.6, 1.4 Hz, 1H), 6.98 – 6.91 (m, 1H), 6.90 – 6.85 (m, 2H), 5.49 (q, *J* = 1.7 Hz, 1H), 3.60 (dd, *J* = 12.0, 6.9 Hz, 1H), 3.40 (d, *J* = 13.3 Hz, 1H), 3.27 (s, 3H), 2.58 (dd, *J* = 17.9, 7.0 Hz, 1H), 2.44 (dt, *J* = 13.2, 2.0 Hz, 1H), 2.35 (dd, *J* = 18.2, 12.2 Hz, 1H), 2.27 – 2.20 (m, 2H), 2.02 (s, 3H), 1.85 (d, *J* = 1.3 Hz, 3H), 1.77 – 1.71 (m, 2H), 1.24 – 1.17 (m, 1H), 1.07 – 1.00 (m, 1H). **^{13}C {1H} NMR** (101 MHz, $CDCl_3$) δ 211.3, 206.9, 178.5, 144.9, 133.7, 129.9, 128.2, 125.4, 124.7, 122.4, 108.1, 54.5, 51.0, 49.7, 46.8, 40.7, 30.7, 28.7, 28.1, 26.7, 23.4, 20.9. **MS** (EI) *m/z*: 351 (M^+, 24%), 43 (65), 161 (41), 186 (40), 201 (40), 202 (100), 308 (61). **HRMS** (ESI): m/z calcd for $C_{22}H_{25}NO_3$ [M^+] 351.1834, found 351.1826. The enantiomeric excess was determined by HPLC using a Chiralpak IA column [*n*-hexanes/2-propanol (80:20)]; flow rate 1 mL/min, λ = 212 nm; τ_{major} = 10.3 min, τ_{minor} = 39.8 min (63% *ee*); **$[\alpha]_D^{25}$** = -15.3 (c 1.0, $CHCl_3$).

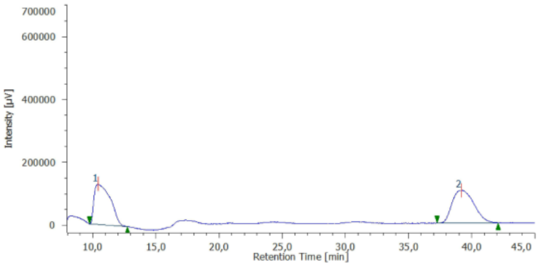

tR [min]	Area [µV·sec]	Height [µV]	Area%	Height%
10,453	11501114	128657	48,014	55,183
39,200	12452431	104487	51,986	44,817

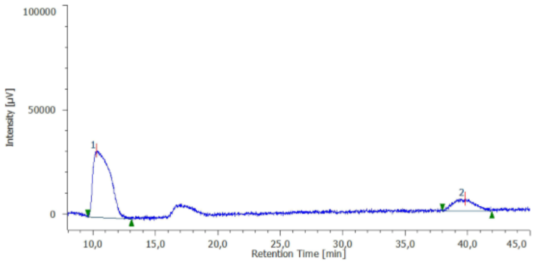

tR [min]	Area [µV·sec]	Height [µV]	Area%	Height%
10,293	3049577	32063	81,271	84,161
39,773	702758	6034	18,729	15,839

(1*S*,1'*S*,6'*R*)-3-hydroxy-6'-(hydroxymethyl)-4'-methyldispiro[cyclohexane-1,2'-cyclohexane-1',3''-indolin]-3'-en-2''-one, **6aa**.

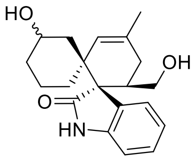

Following procedure detailed in section 4, starting from **3aa** compound **6aa** was obtained as a mixture of diastereoisomers (70:30) as white solid (15 mg, 46%; reaction ran for 16 h). **IR** (neat) ν_{max}: 3344, 2924, 2851, 2360, 1697, 1685, 1617, 1471, 1233, 1046, 746, 669 cm^{-1}. **MP**: 113-118 °C. **^{1}H NMR** (400 MHz, CD_3OD, major diastereosiomer) δ 7.17 (td, *J* = 7.5, 1.7 Hz, 1H), 7.01 – 6.88 (m, 2H), 6.86 (dd, *J* = 7.7, 1.1 Hz, 1H), 5.72 (q, *J* = 1.7 Hz, 1H), 3.73 – 3.65 (m,, *J* = 11.4, 4.5 Hz, 1H), 2.87 (tdd, *J* = 9.8, 7.0, 4.5 Hz, 1H), 2.75 – 2.67 (m, 1H), 2.60 – 2.51 (m, 1H), 2.18 (t, *J* = 11.8 Hz, 1H), 2.01 (ddt, *J* = 12.1, 4.2, 2.1 Hz, 1H), 1.93 – 1.88 (m, 1H), 1.86 (d, *J* = 1.3 Hz, 3H), 1.60 – 1.49 (m, 1H), 1.48 – 1.41 (m, 1H), 1.39 – 1.29 (m, 1H), 1.22 – 1.15 (m, 1H), 1.11 – 0.98 (m, 1H), 0.93 – 0.85 (m, 1H), 0.78 (td, *J* = 13.0, 4.6 Hz, 1H). **^{1}H NMR** (400 MHz, CD_3OD, representative signals for the minor diastereosiomer) δ 5.89 (q, *J* = 1.7 Hz, 1H), 4.16 (p, *J* = 3.6 Hz, 1H), 2.66 – 2.62 (m, 1H), 2.47 (d, *J* = 6.9 Hz, 1H), 1.95 (dt, *J* = 10.2, 1.5 Hz, 1H), 1.82 (d, *J* = 1.2 Hz, 3H). **^{13}C {1H} NMR** (101 MHz, CD_3OD, major diastereosiomer) δ 182.4, 143.2, 134.4, 131.5, 128.9, 127.3, 126.9, 122.9, 110.5, 68.3, 65.4, 58.6, 44.9, 44.6, 38.9, 36.1, 33.1, 30.1, 23.5, 20.6. **^{13}C {1H} NMR** (101 MHz, CD_3OD, representative signals for the minor diastereosiomer) δ 183.0, 143.2, 131.1, 131.1, 128.8, 127.2, 110.4, 68.0, 65.4, 58.9, 42.7, 40.9, 39.1, 33.0, 32.9, 31.6, 23.5, 17.4. **MS** (EI) *m/z*: 327 (M^+, 18%), 216 (18), 215 (100), 207 (15), 177 (13), 176 (76), 175 (35), 159 (15), 158 (64), 152 (12). **HRMS** m/z calcd for $C_{20}H_{25}NO_3$ [M^+] 327.1834, found 327.1820. **$[\alpha]_D^{25}$** = **- 80.8** (c 0.5, MeOH).

6. NMR Spectra for all compounds.

^{1}H NMR (400 MHz, $CDCl_3$) **3aa**.

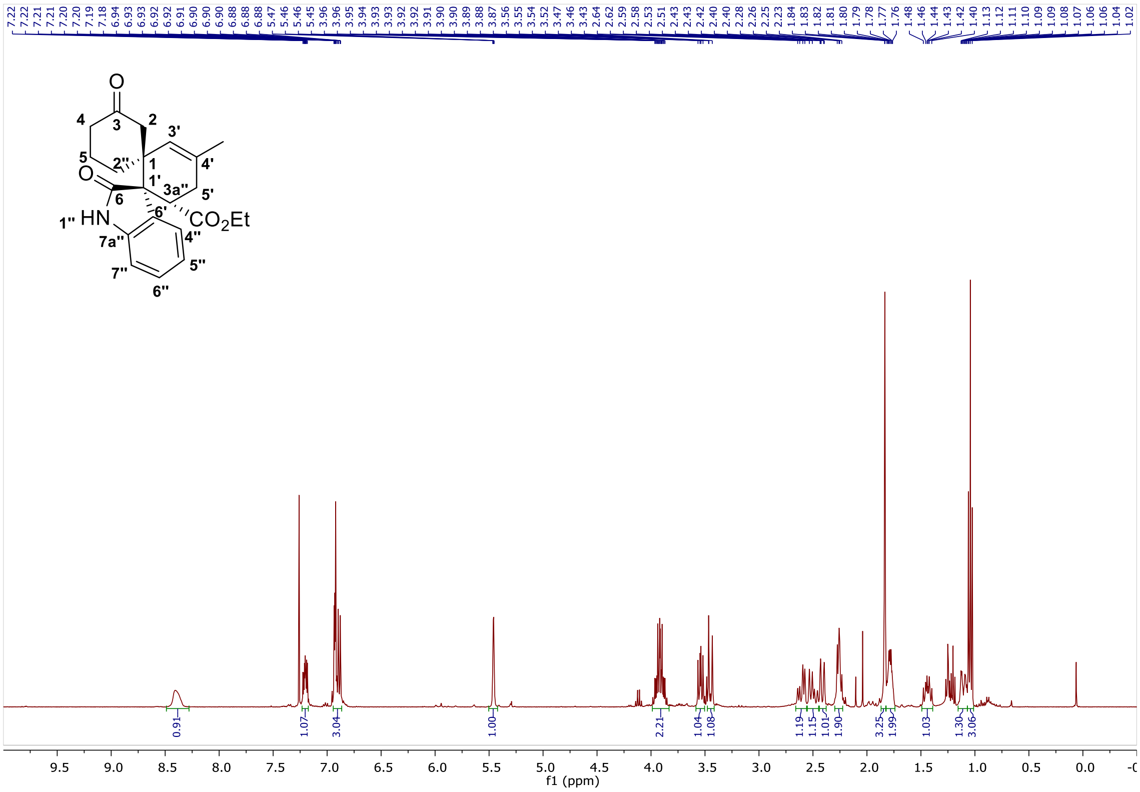

^{13}C {1H} NMR (101 MHz, $CDCl_3$) of **3aa**.

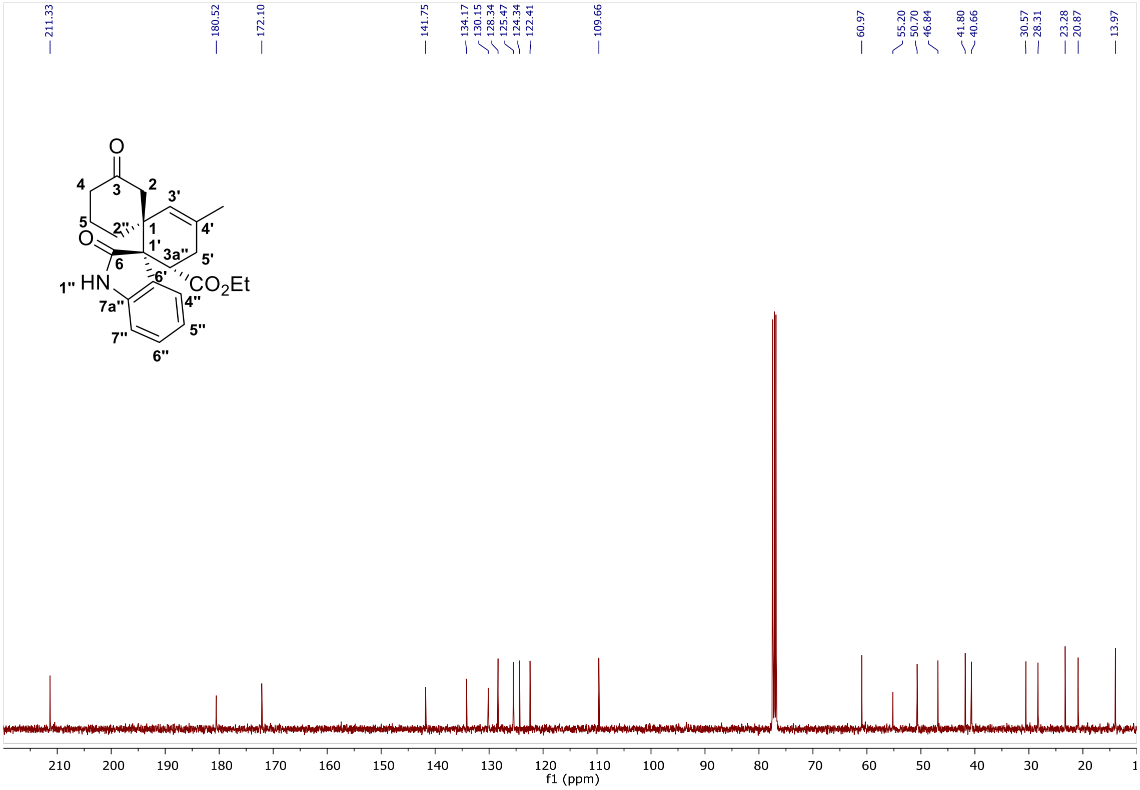

^{1}H NMR (400 MHz, CDCl₃) **3ab**.

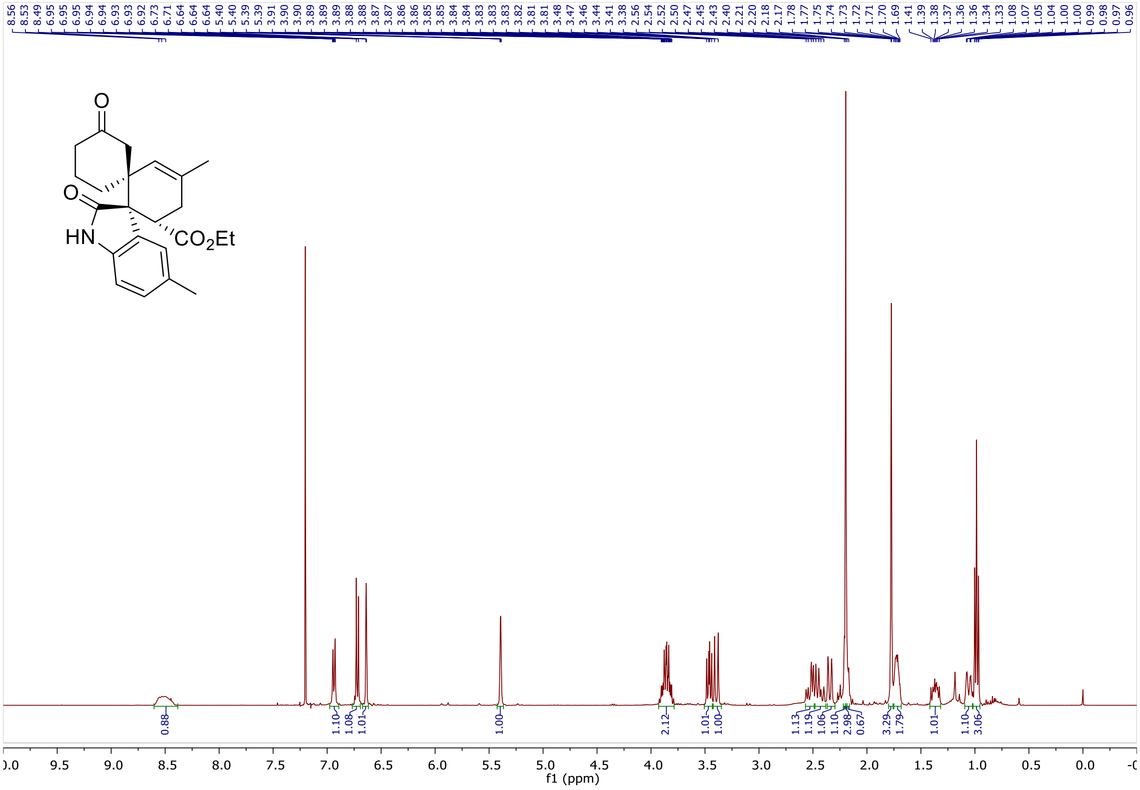

^{13}C {1H} NMR (101 MHz, CDCl₃) of **3ab**.

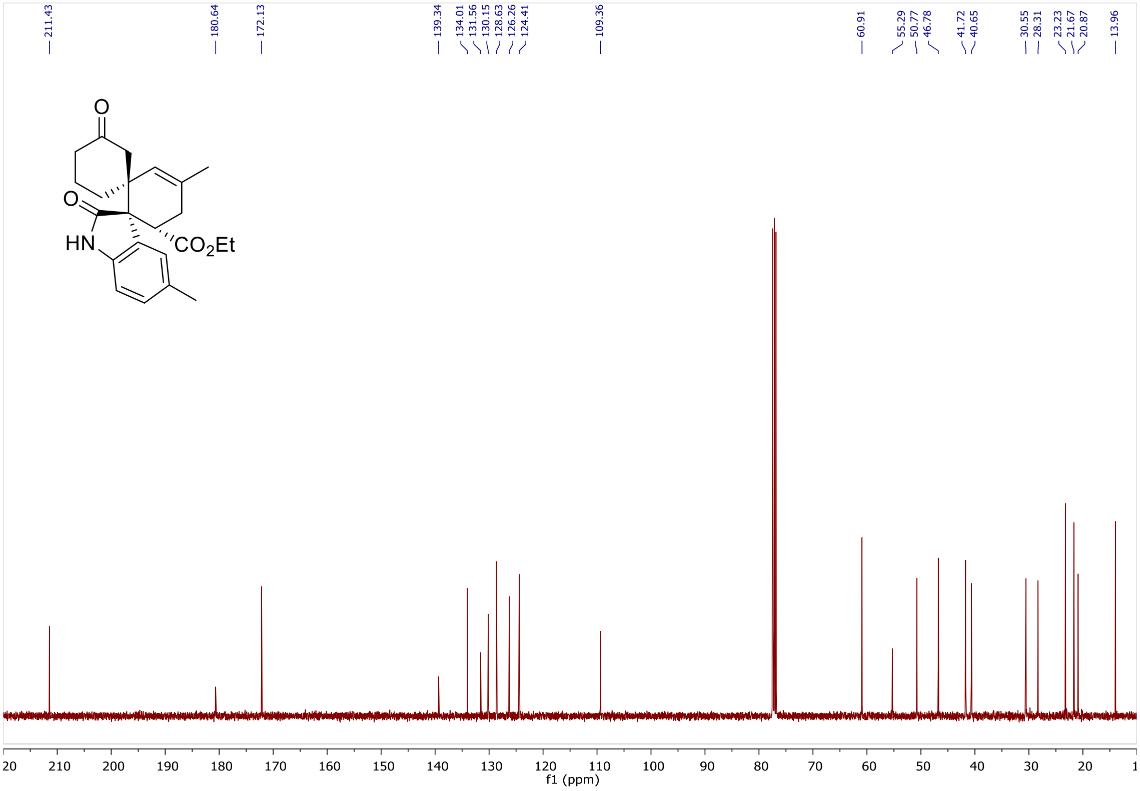

^{1}H NMR (400 MHz, $CDCl_3$) **3ac'**.

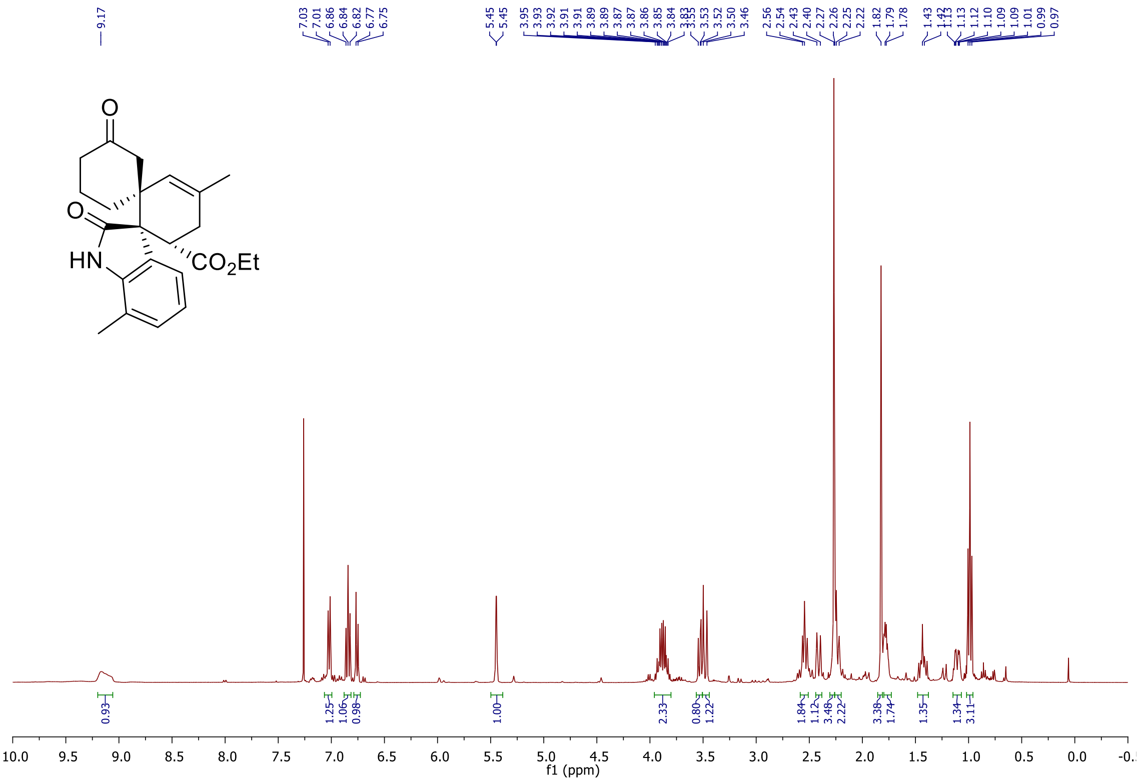

^{13}C {1H} NMR (101 MHz, $CDCl_3$) of **3ac'**.

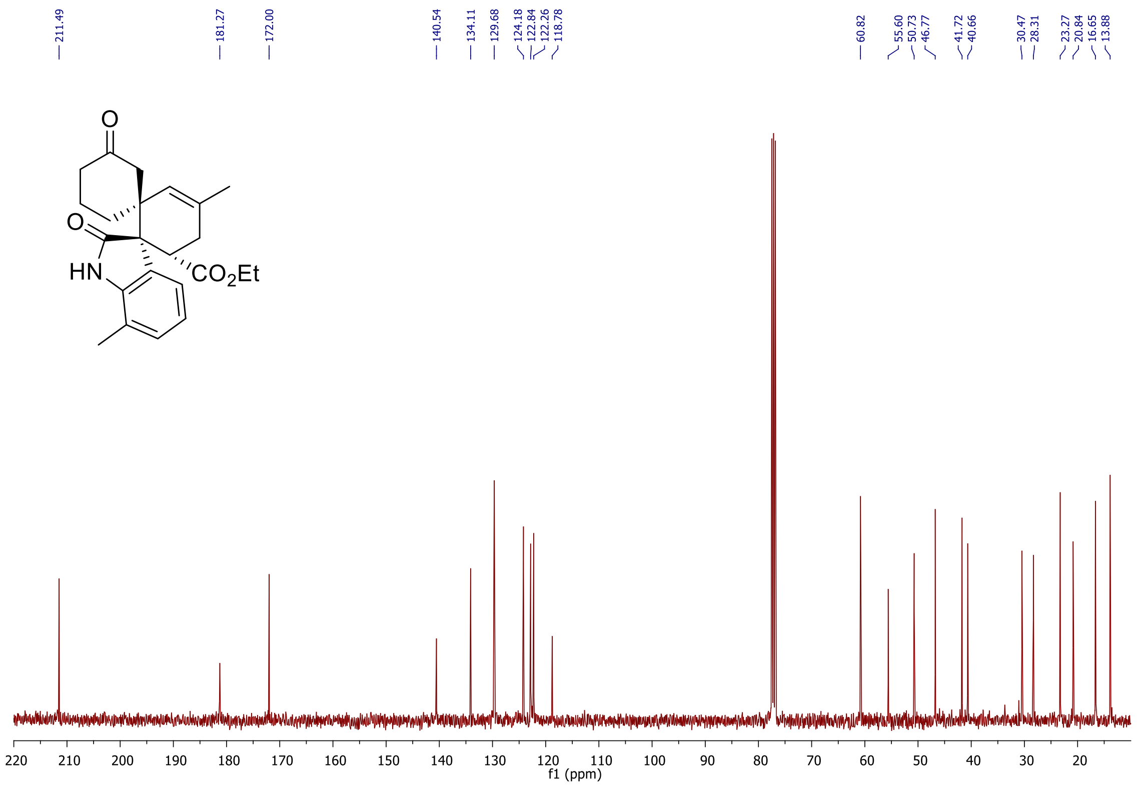

^{1}H NMR (400 MHz, $CDCl_3$) **3ad**.

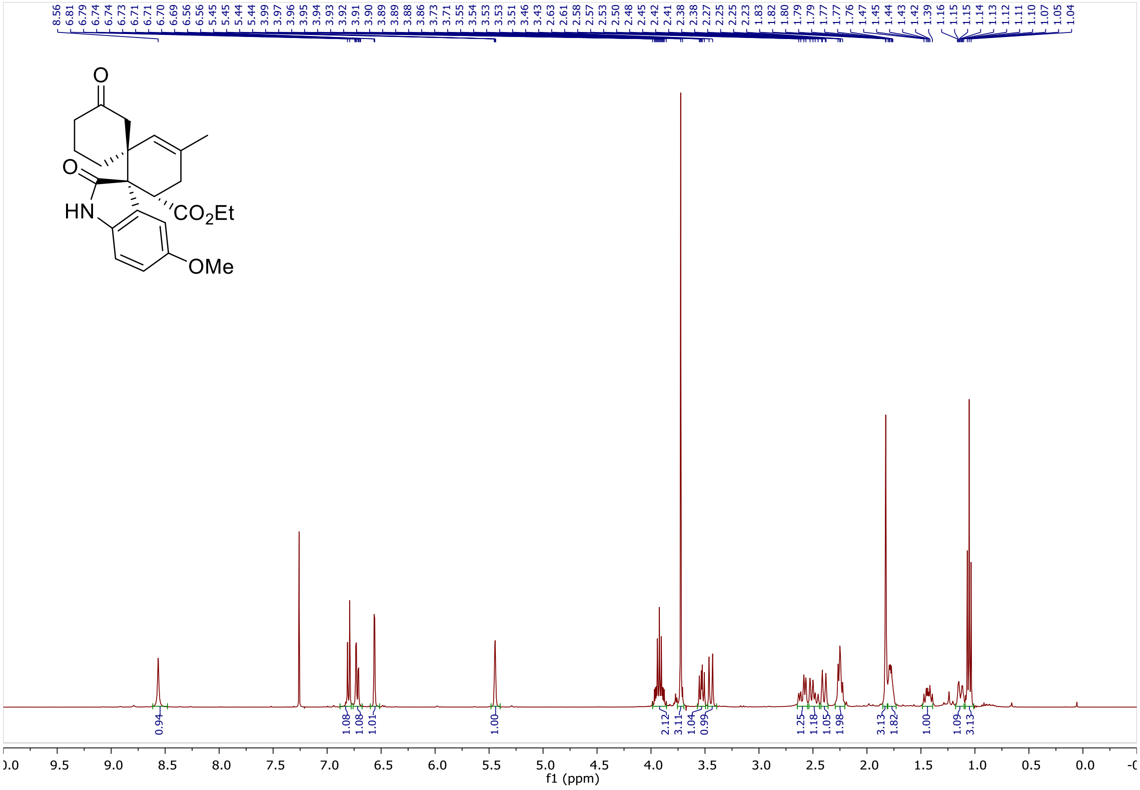

^{13}C {1H} NMR (101 MHz, $CDCl_3$) of **3ad**.

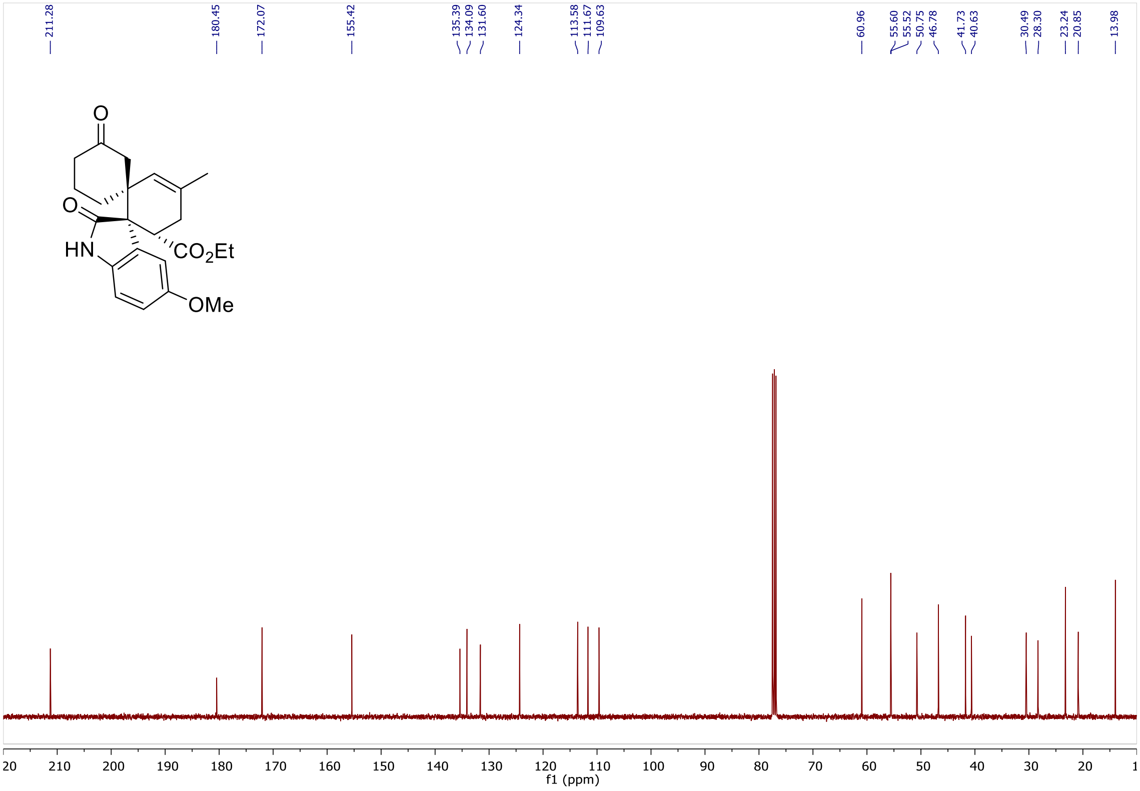

^{1}H NMR (400 MHz, $CDCl_3$) **3ae**.

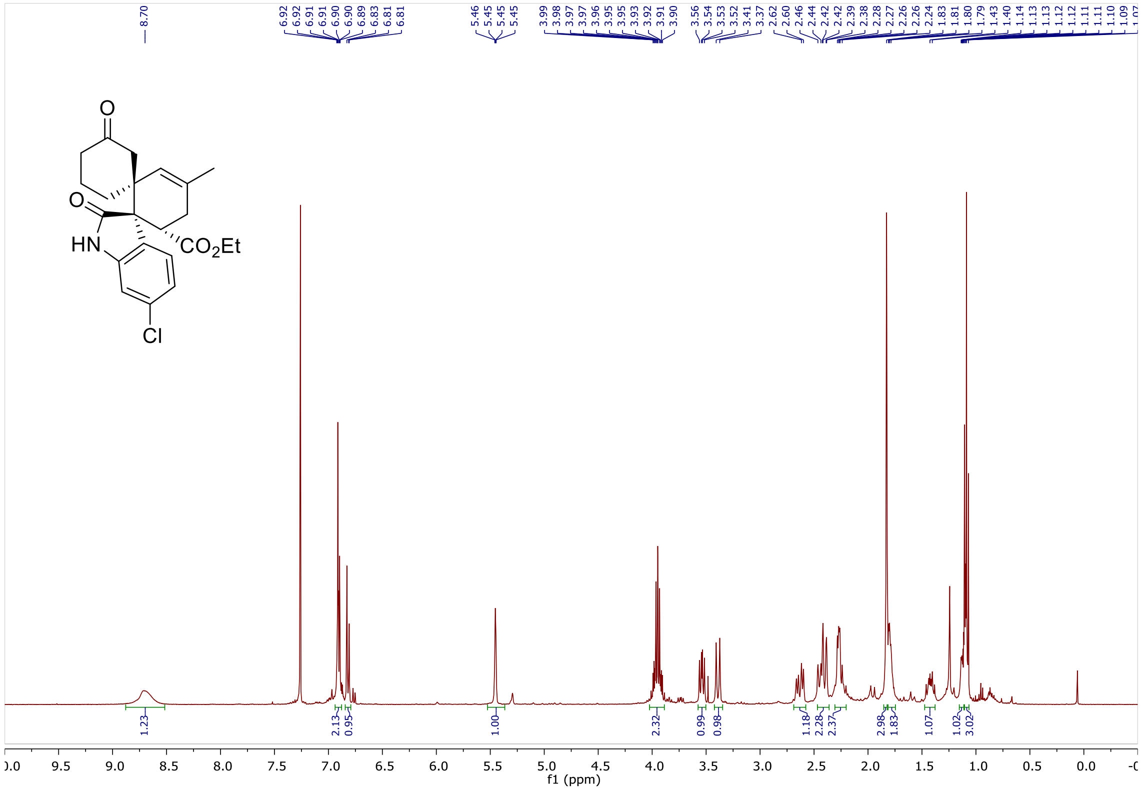

^{13}C {1H} NMR (101 MHz, $CDCl_3$) of **3ae**.

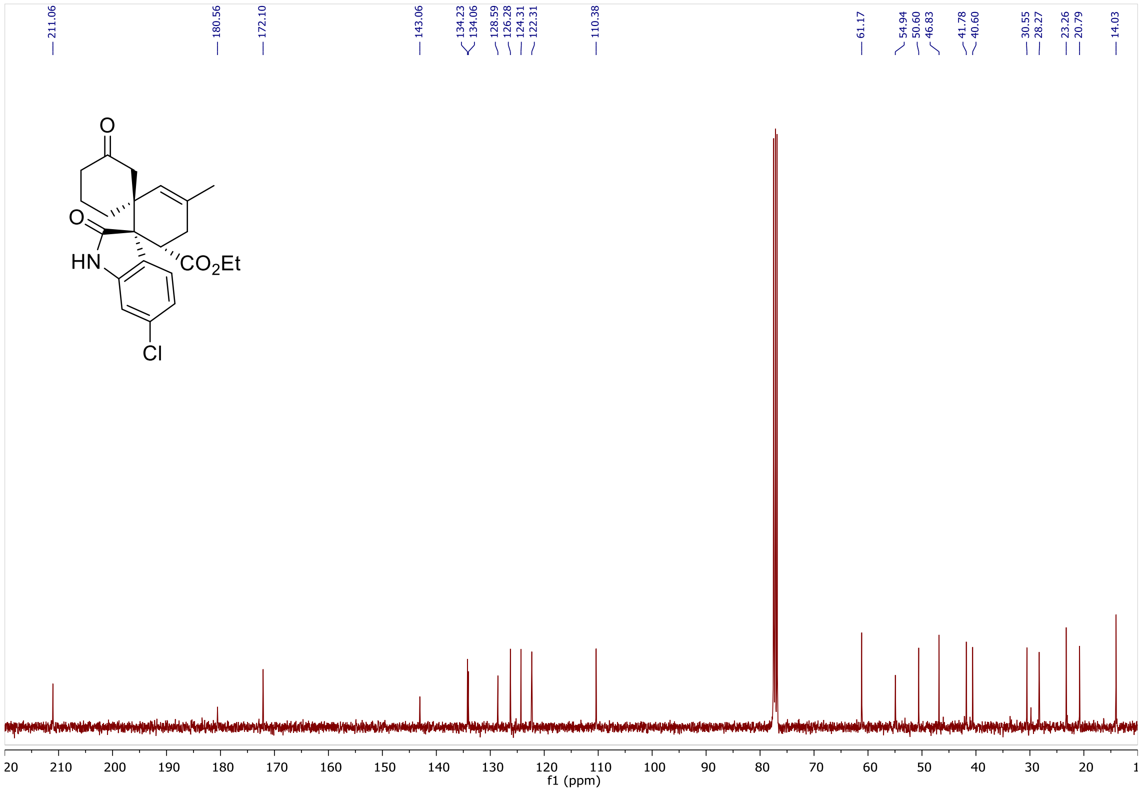

[1]**H NMR** (400 MHz, CDCl₃) **3af**.

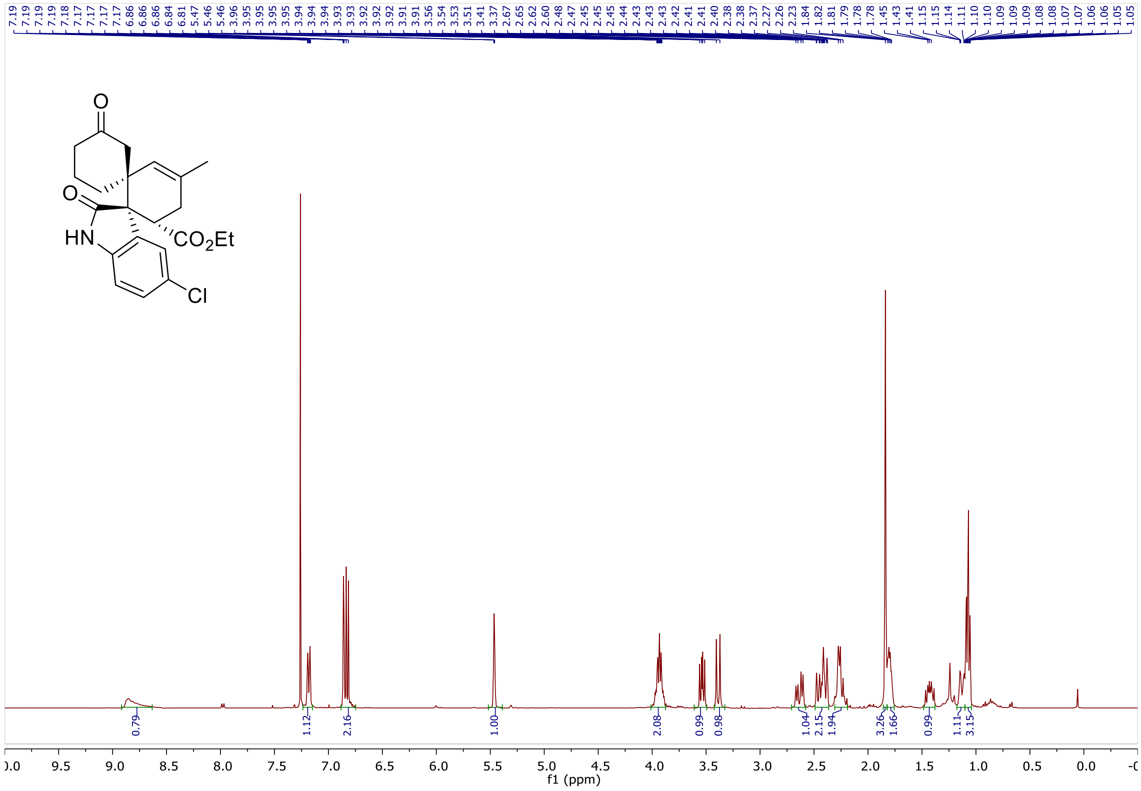

[13]**C {1H} NMR** (101 MHz, CDCl₃) of **3af**.

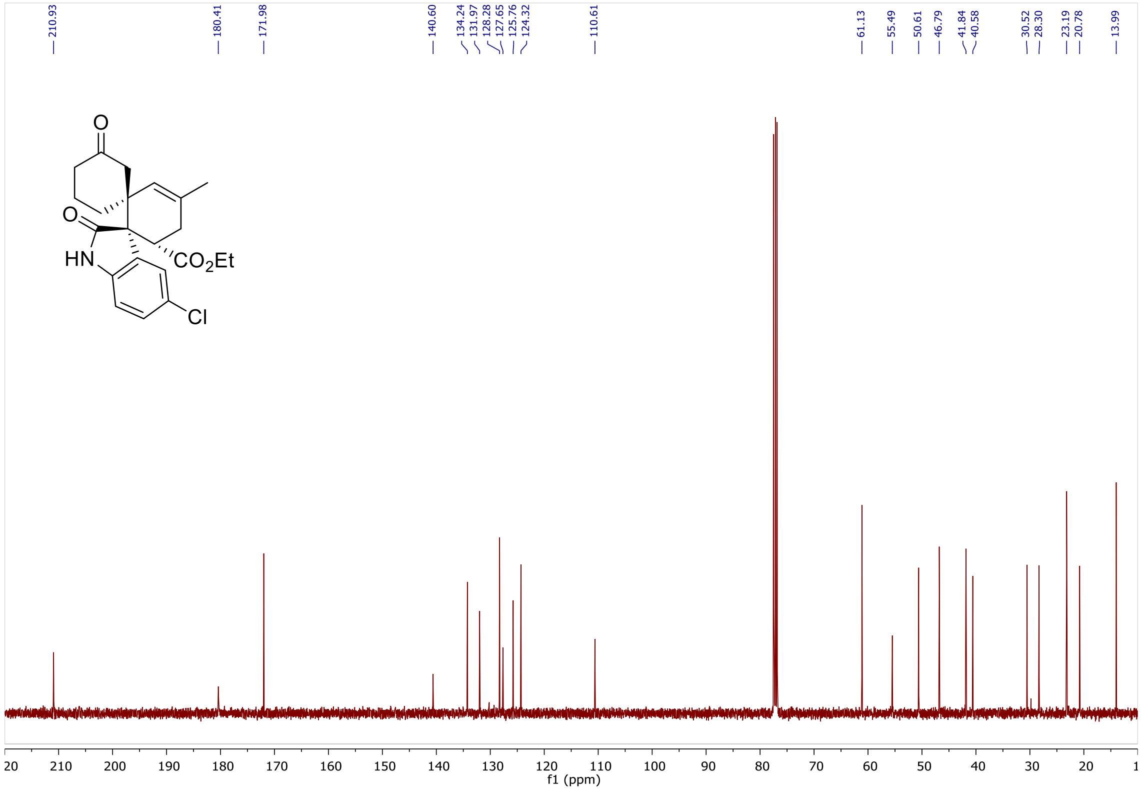

^{1}H NMR (400 MHz, $CDCl_3$) **3ag**.

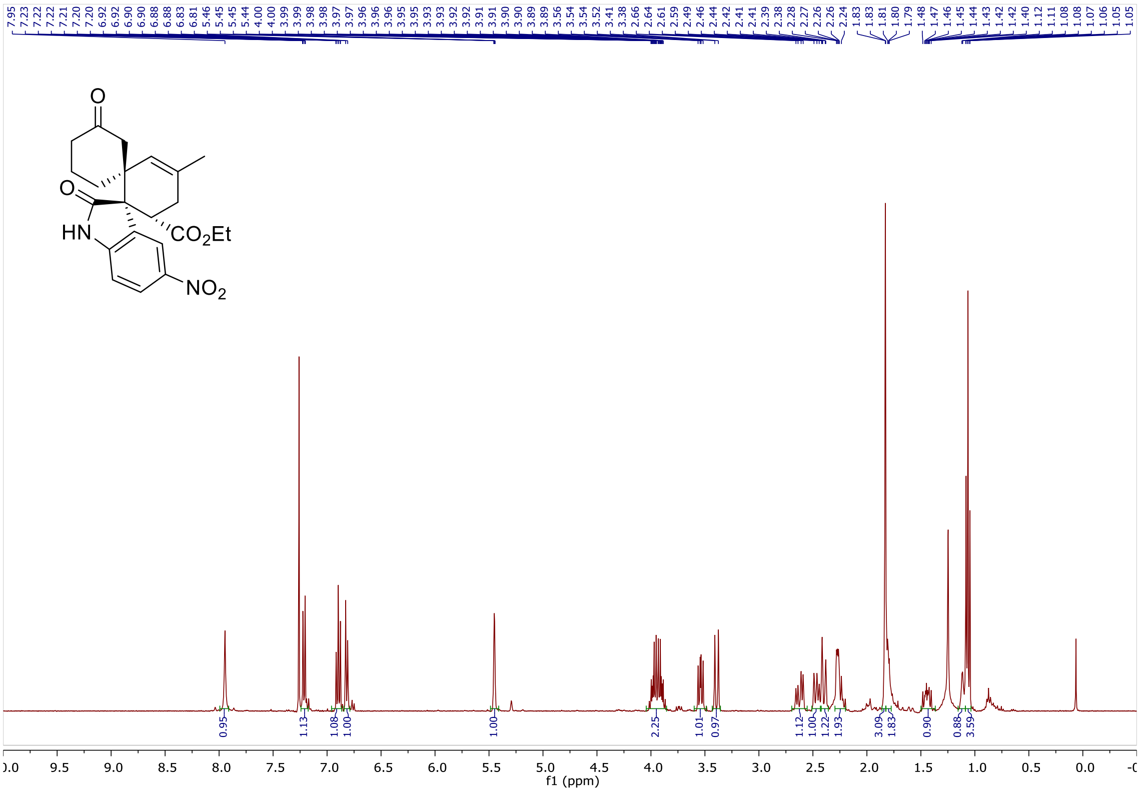

^{13}C {1H} NMR (101 MHz, $CDCl_3$) of **3ag**.

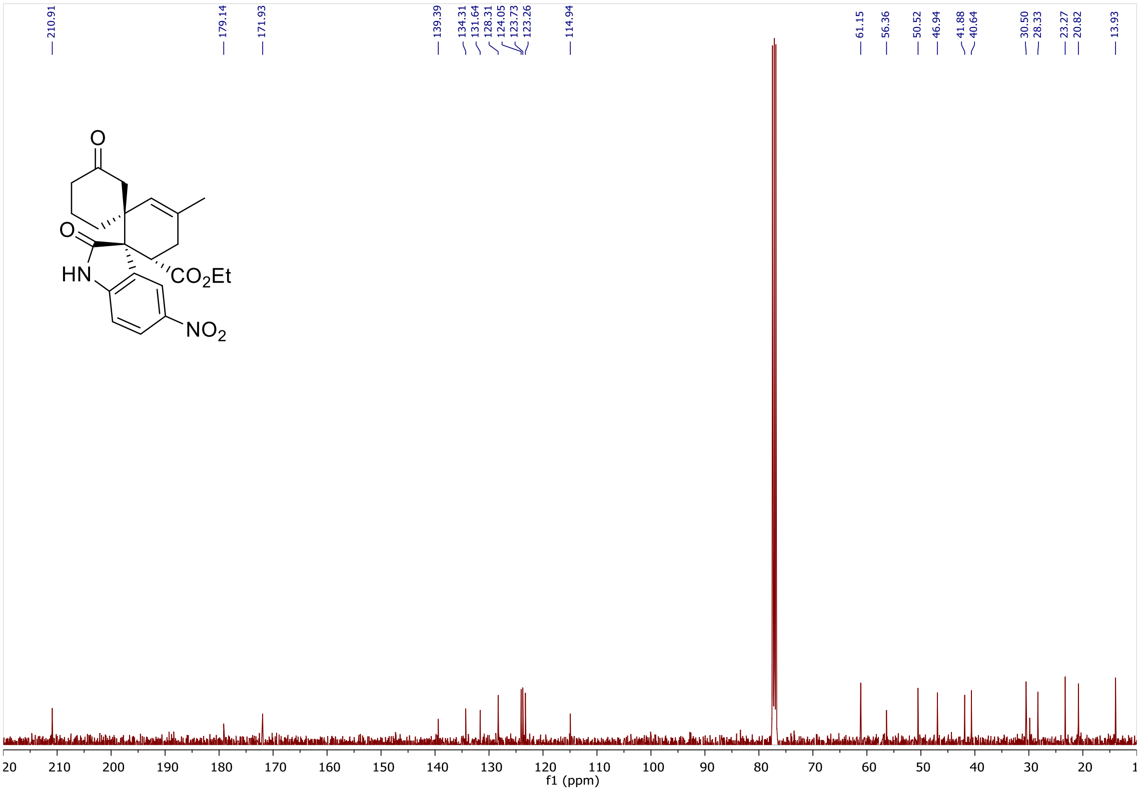

^{1}H NMR (400 MHz, $CDCl_3$) **3ah**.

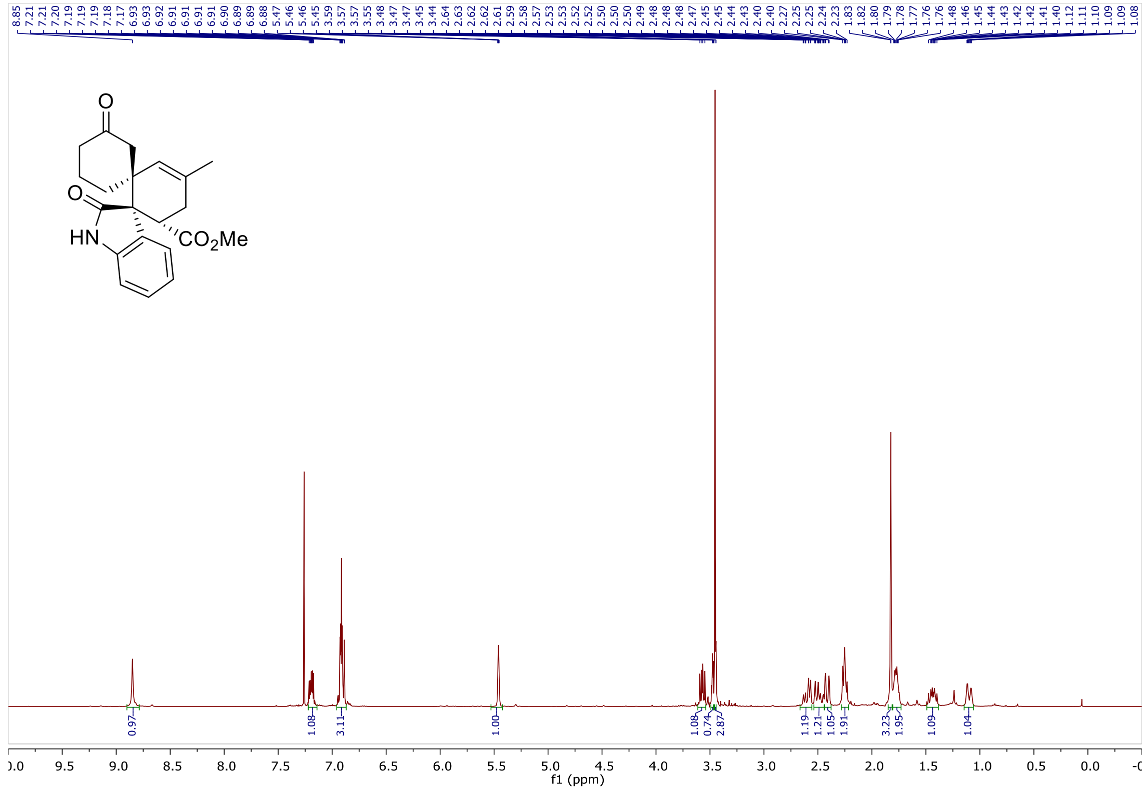

^{13}C {1H} NMR (101 MHz, $CDCl_3$) of **3ah**.

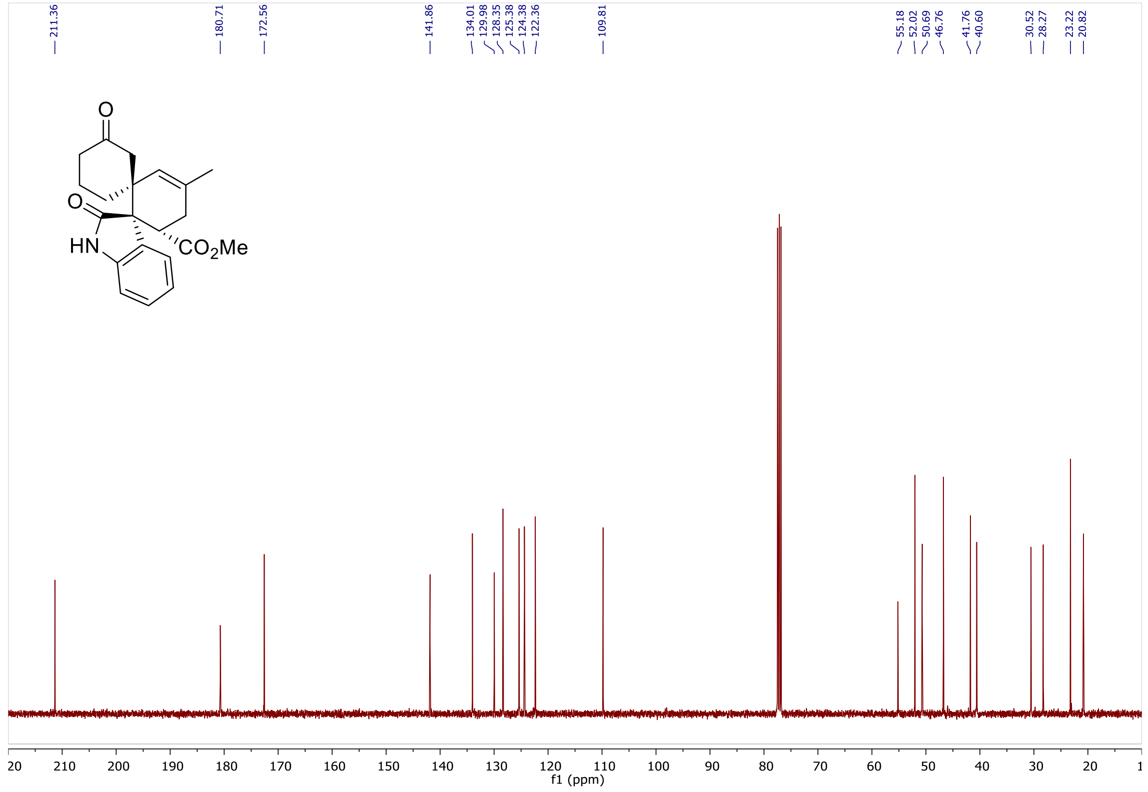

^{1}H NMR (400 MHz, CDCl₃) **3ai**.

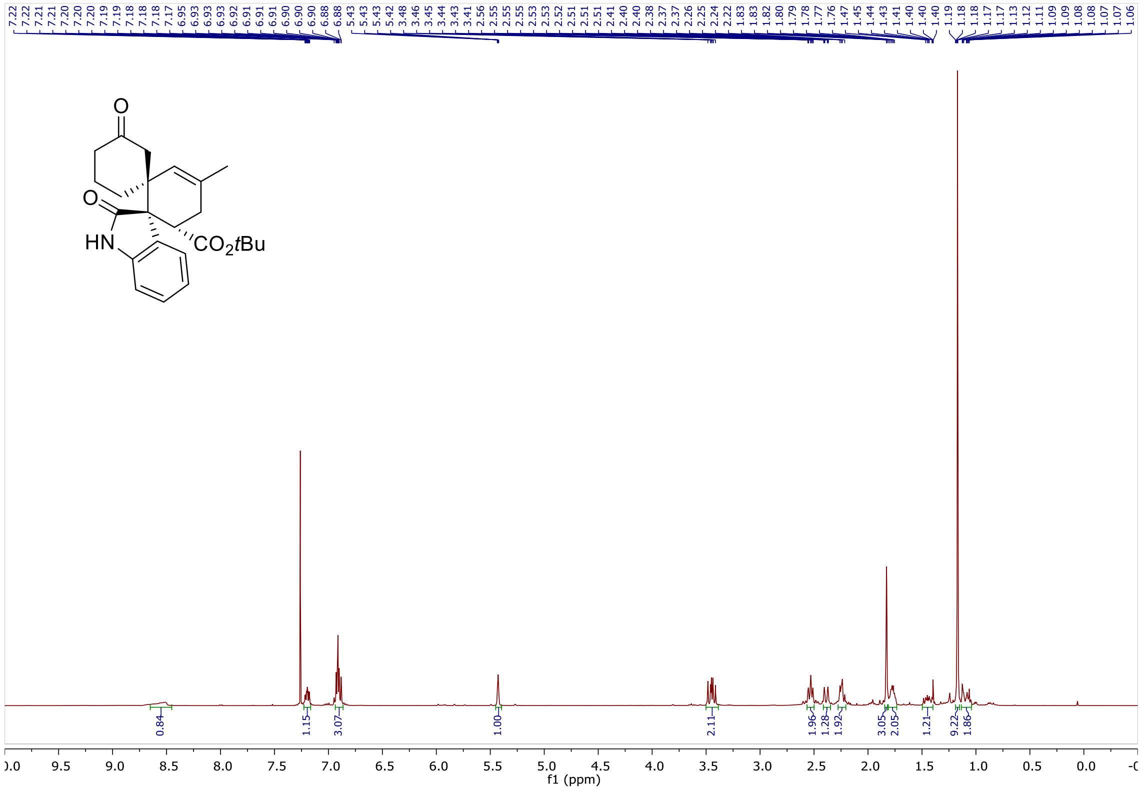

^{13}C {1H} NMR (101 MHz, CDCl₃) of **3ai**.

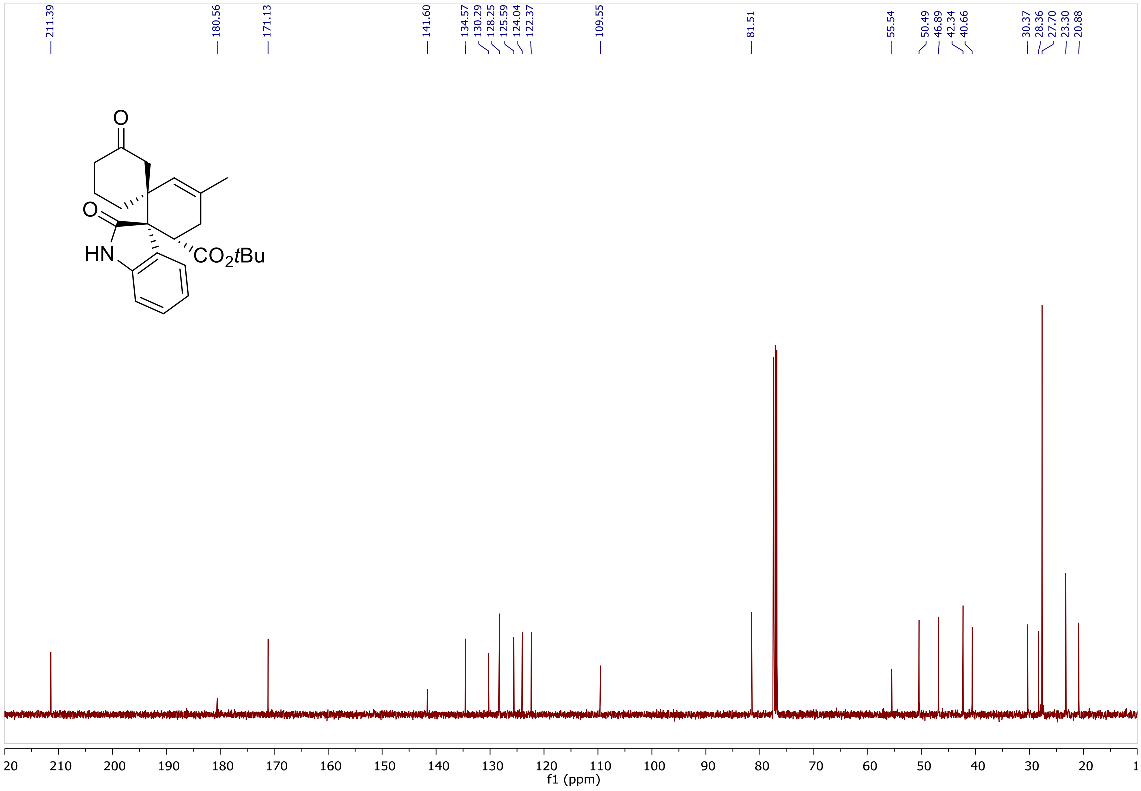

[1]**H NMR** (400 MHz, CDCl₃) **3aj**.

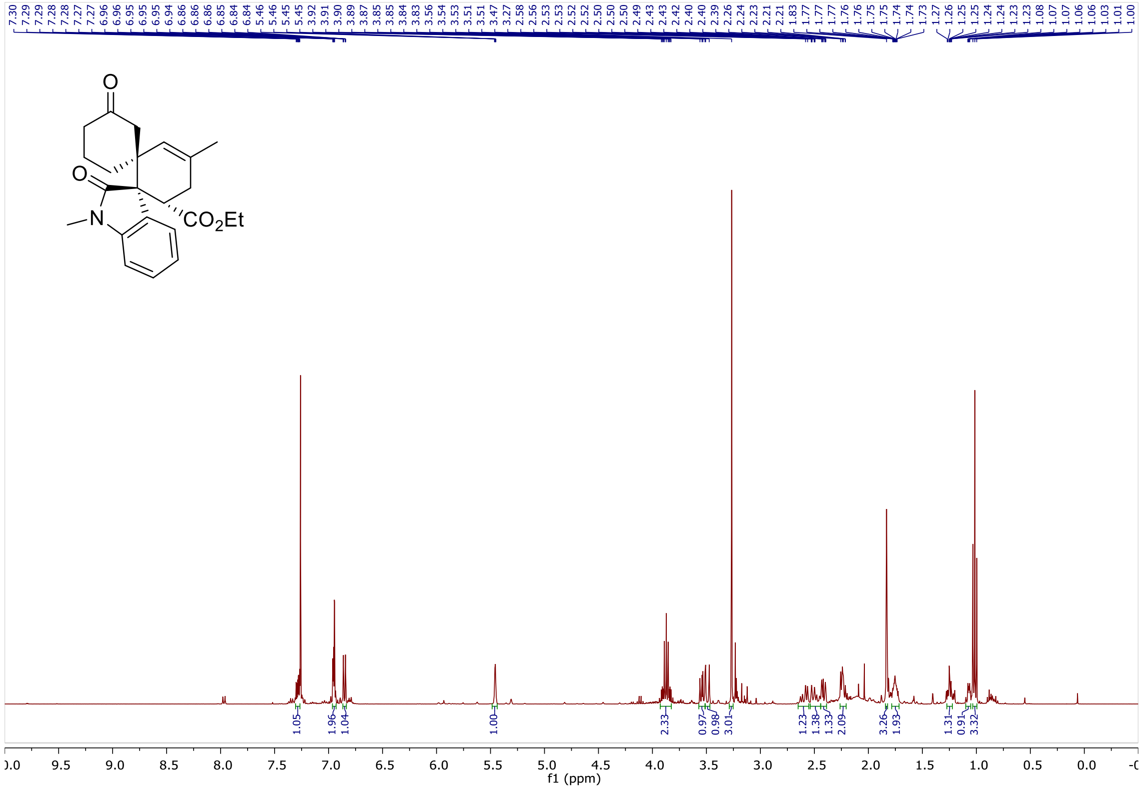

[13]**C {1H} NMR** (101 MHz, CDCl₃) of **3aj**.

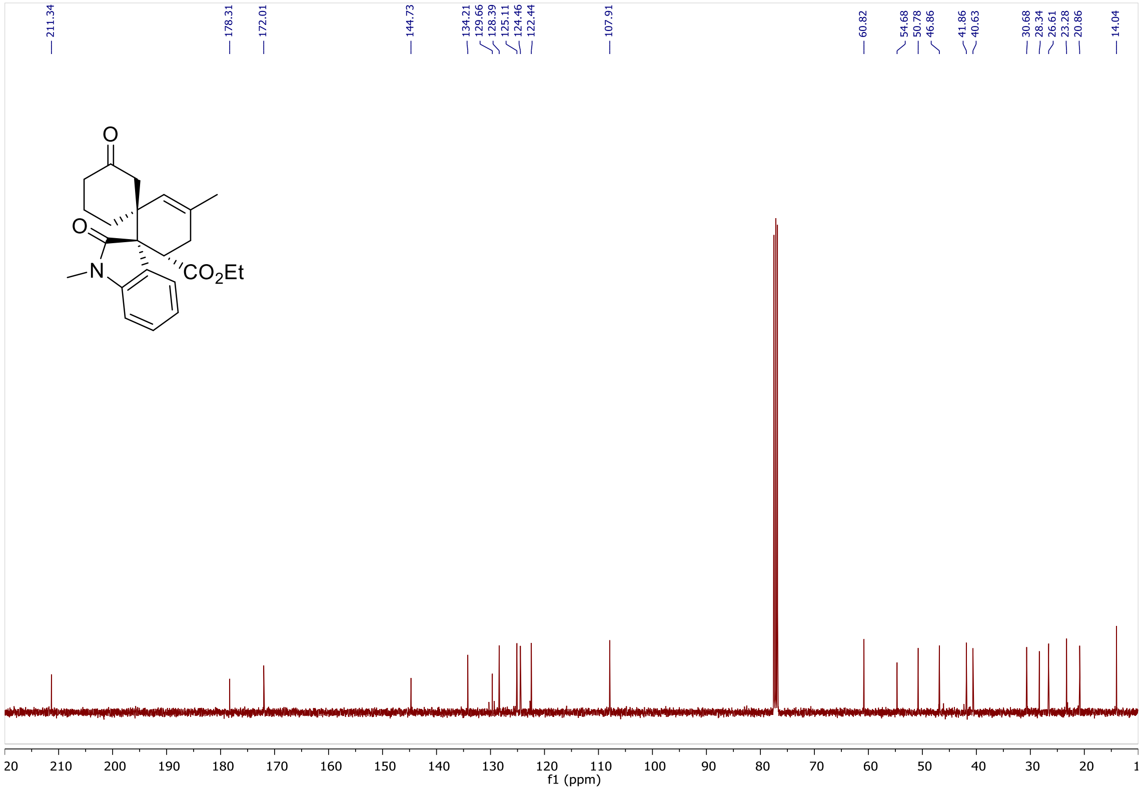

^{1}H NMR (400 MHz, CDCl₃) **3ak**.

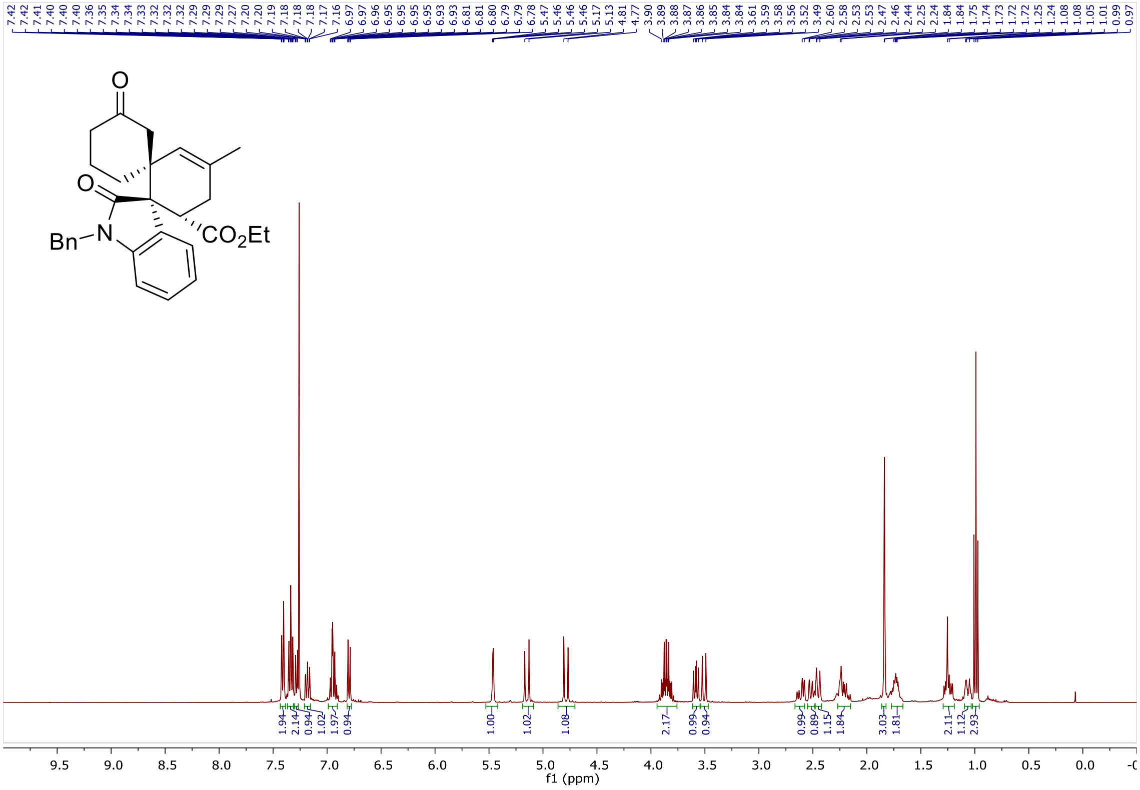

^{13}C {1H} NMR (101 MHz, CDCl₃) of **3ak**.

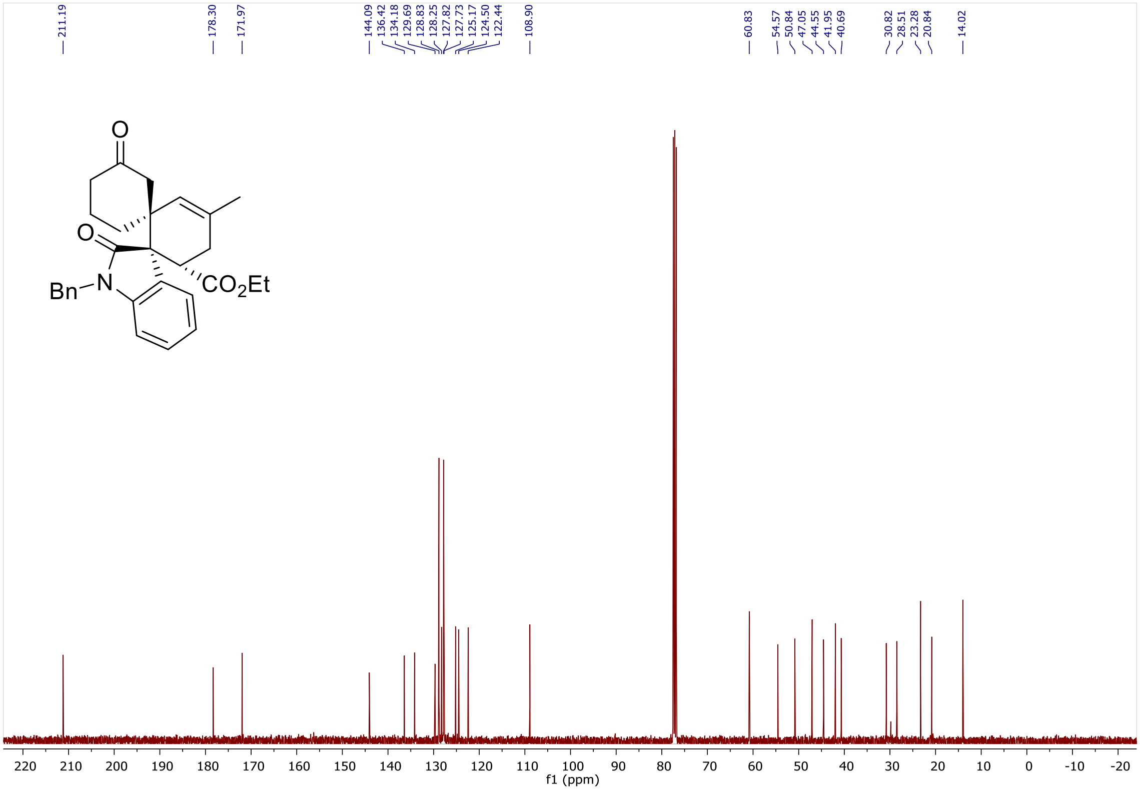

^{1}H NMR (400 MHz, CDCl3) **3al**.

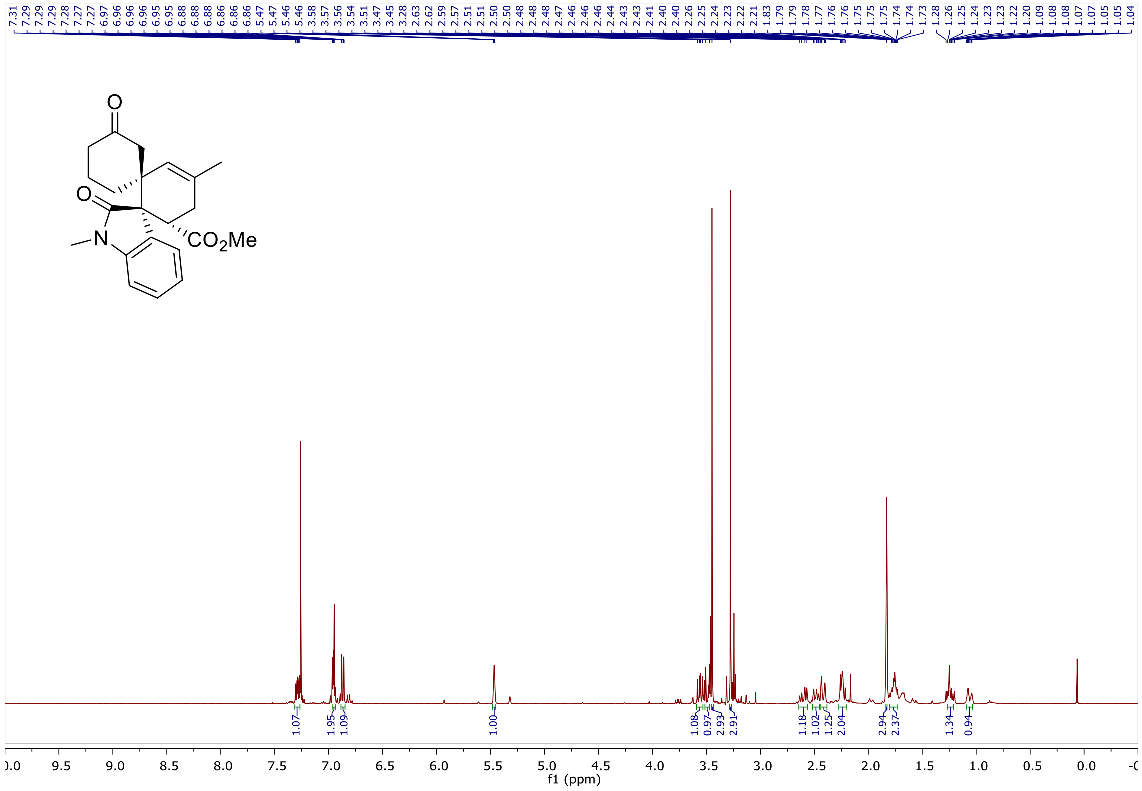

^{13}C {1H} NMR (101 MHz, CDCl3) of **3al**.

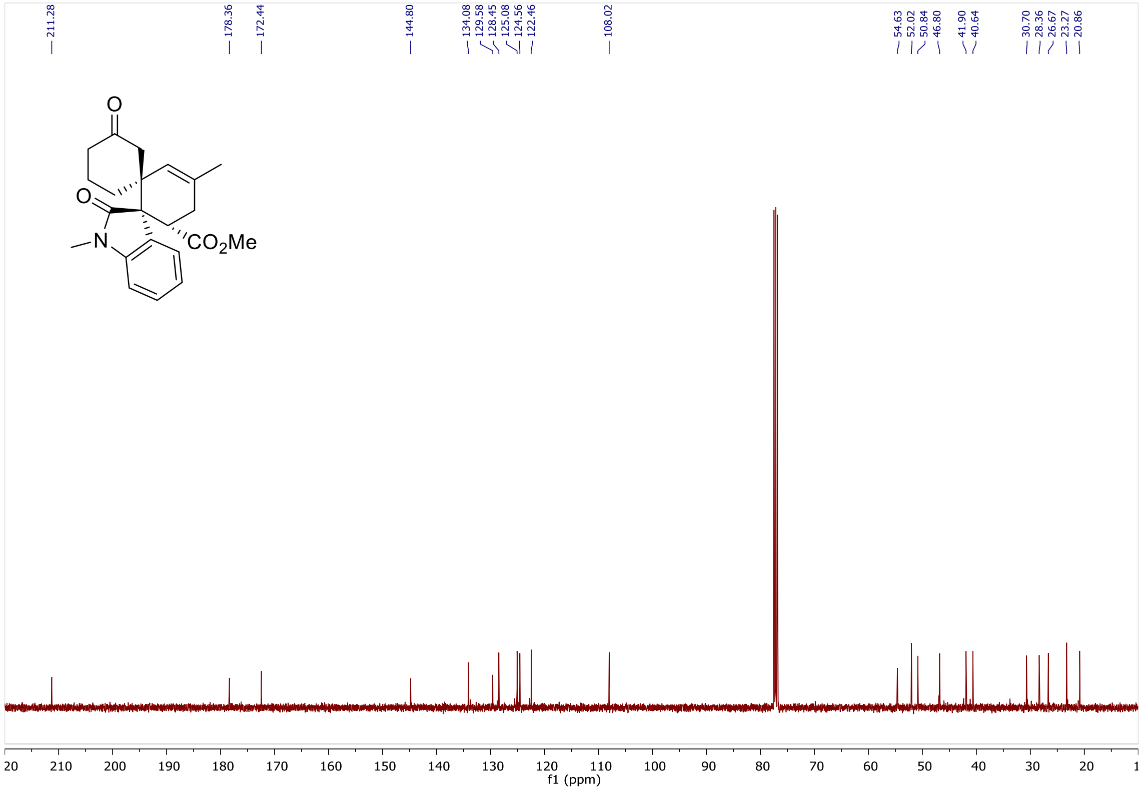

^{1}H NMR (400 MHz, $CDCl_3$) **3am**.

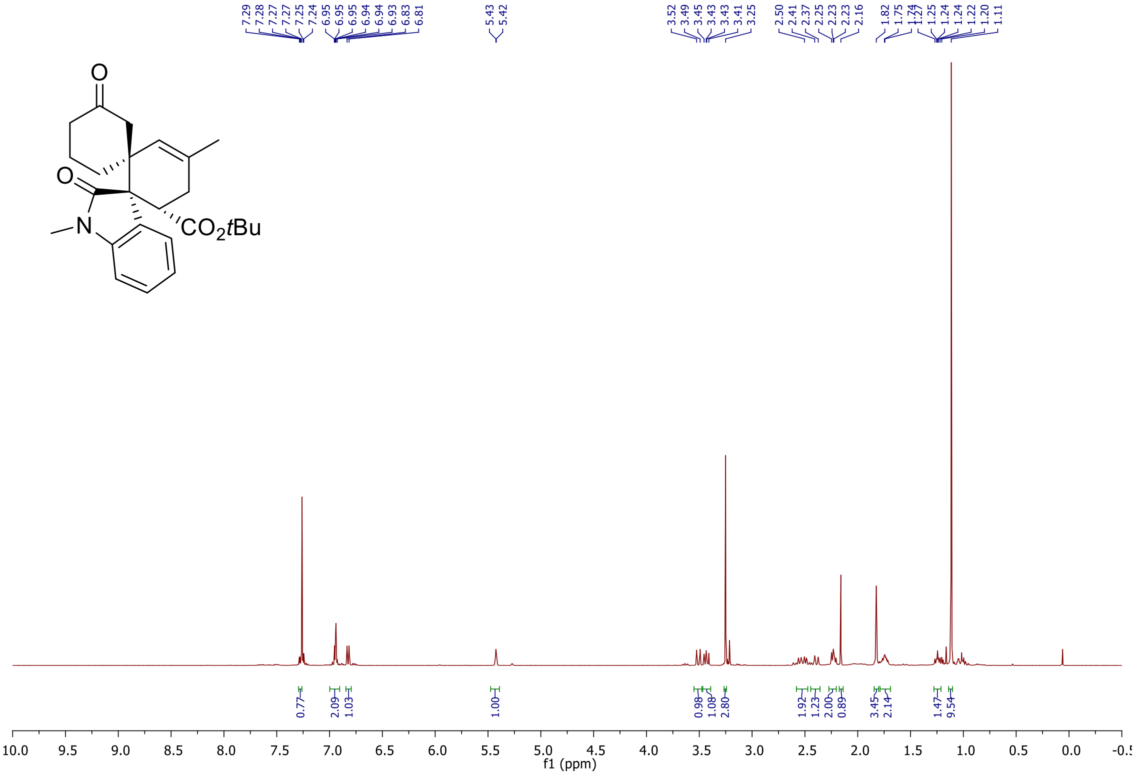

^{13}C {1H} NMR (101 MHz, $CDCl_3$) of **3am**.

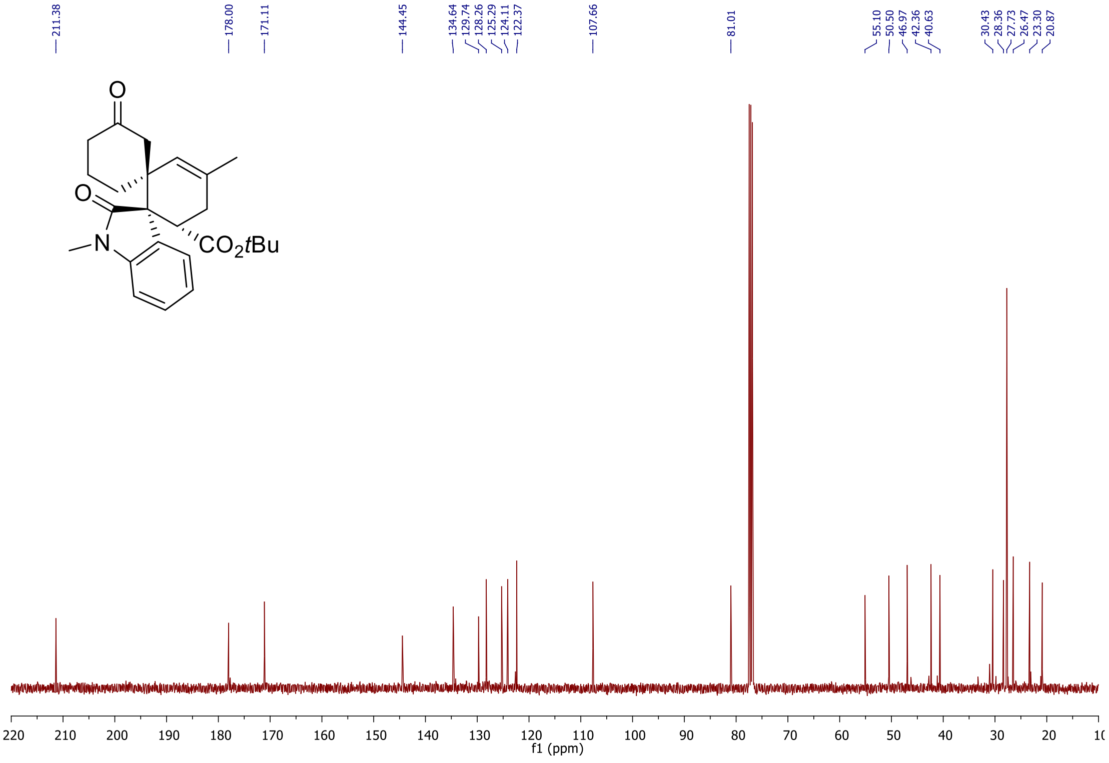

^{1}H NMR (400 MHz, $CDCl_3$) **3ba''**.

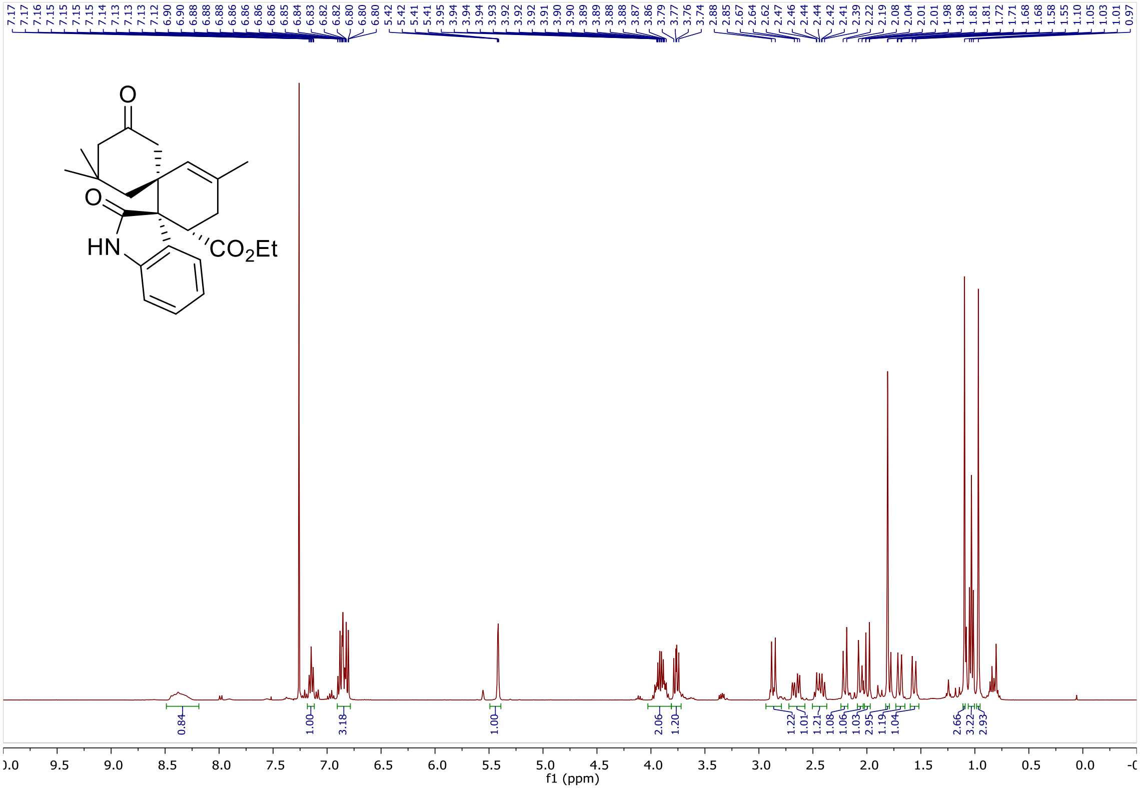

^{13}C {1H} NMR (101 MHz, $CDCl_3$) of **3ba''**.

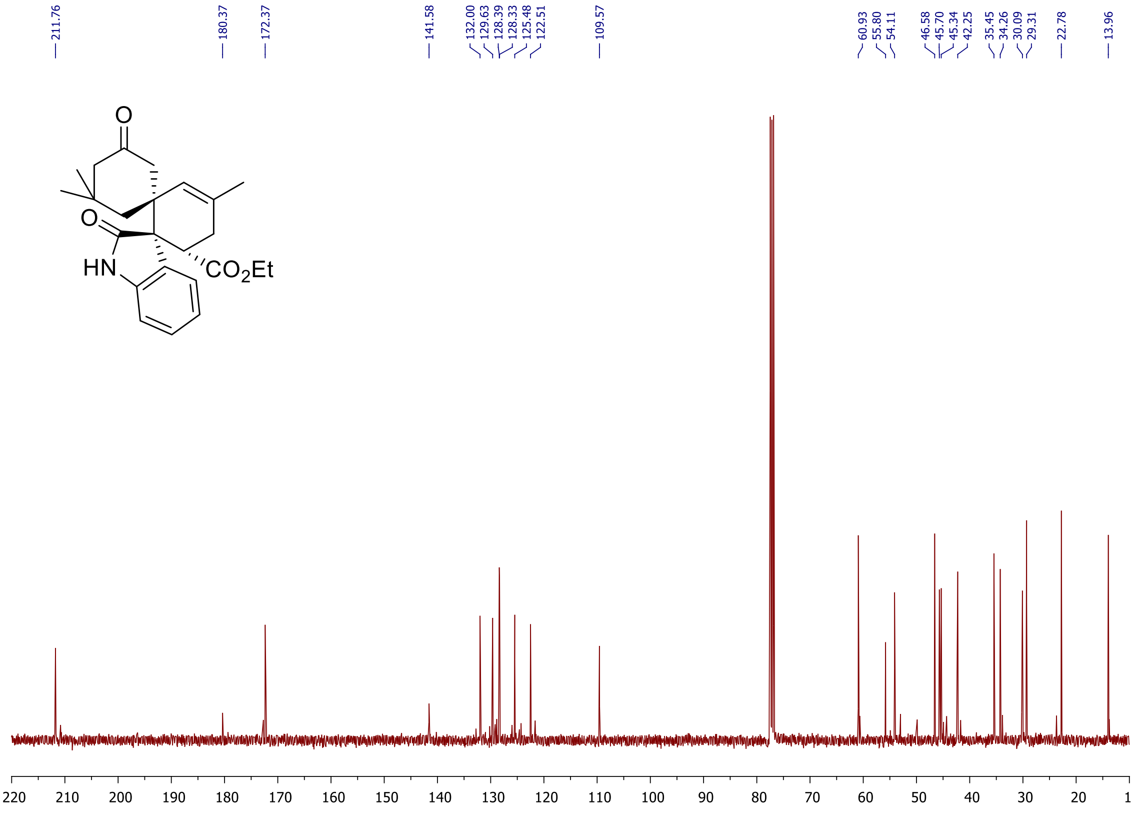

¹H NMR (400 MHz, CDCl₃) **5aa**.

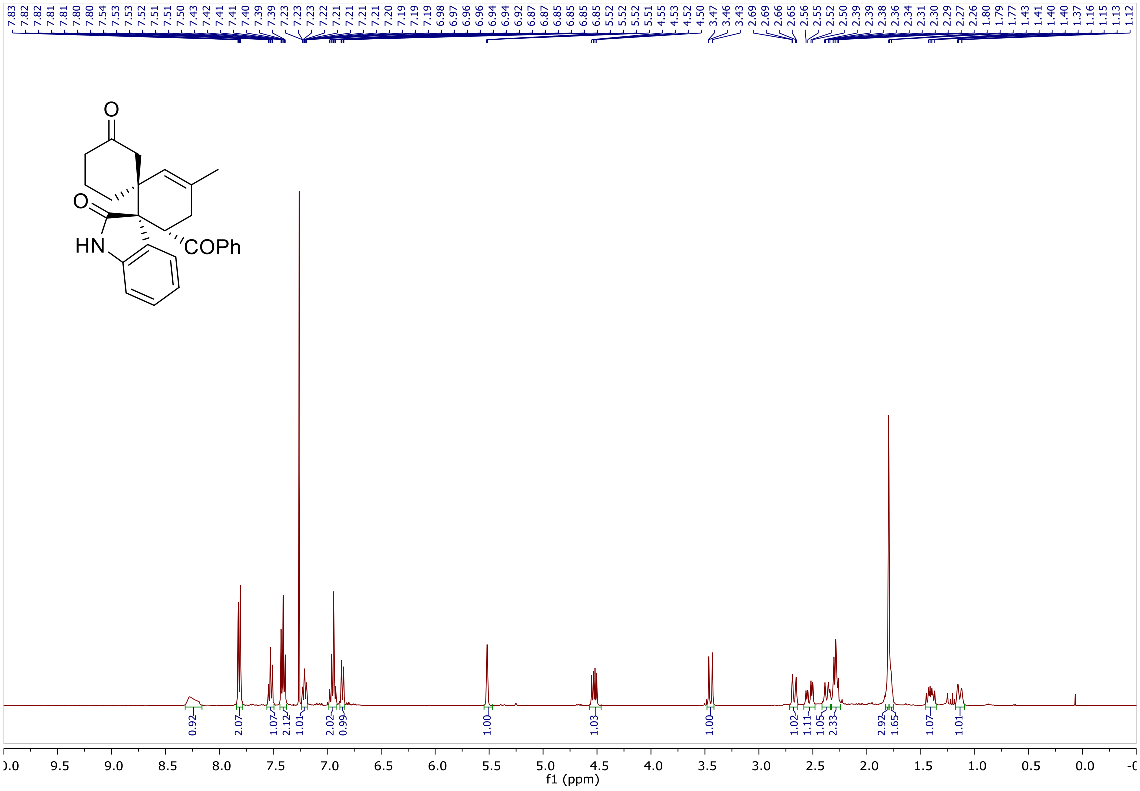

¹³C {1H} NMR (101 MHz, CDCl₃) of **5aa**.

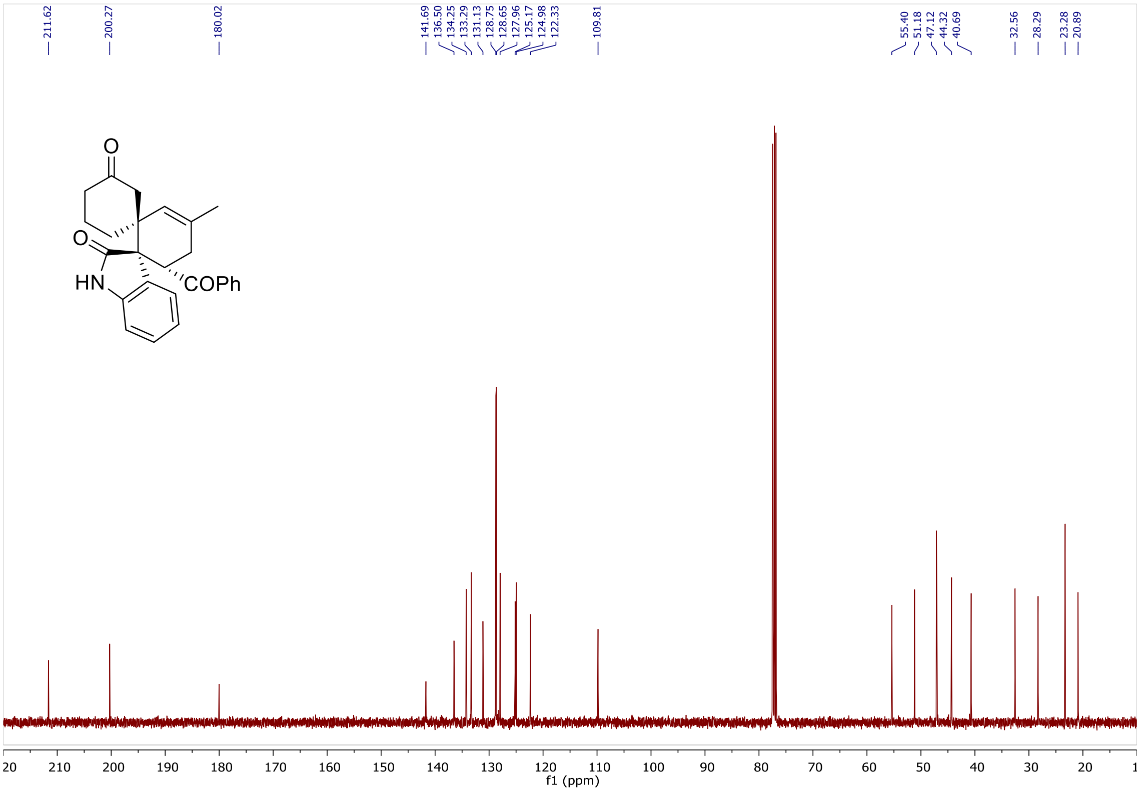

^{1}H NMR (400 MHz, $CDCl_3$) **5ab**.

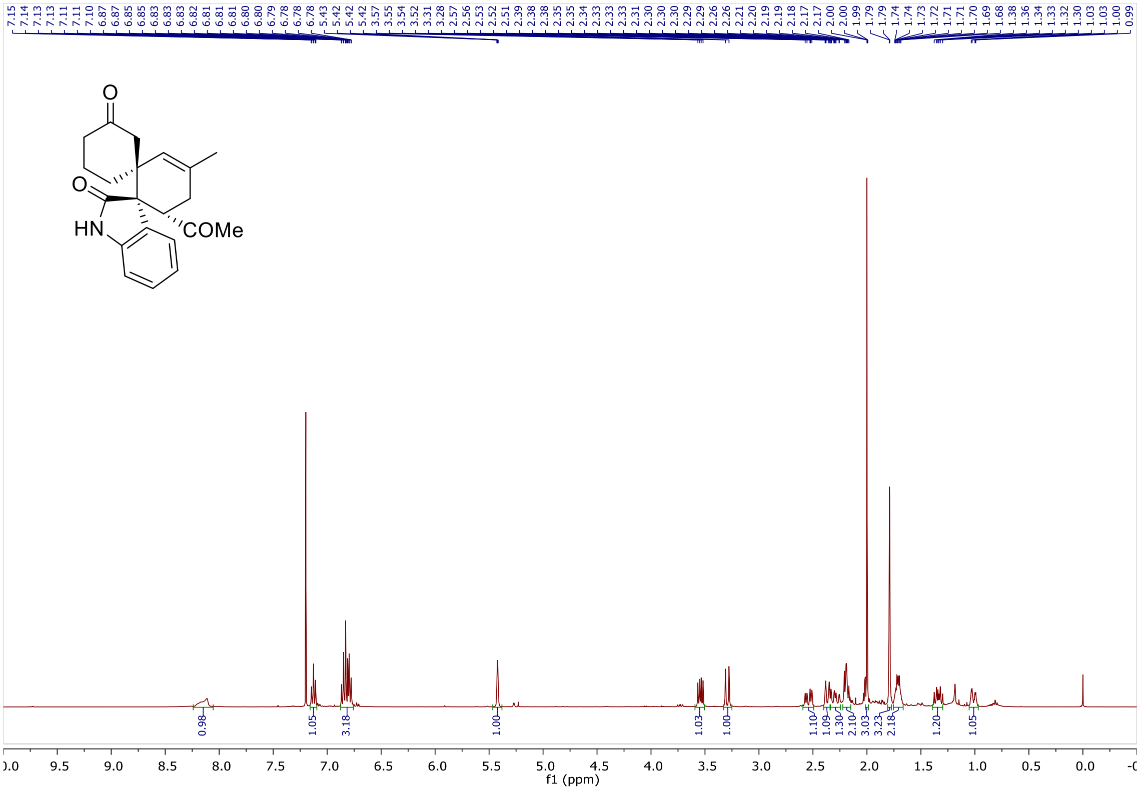

^{13}C {1H} NMR (101 MHz, $CDCl_3$) of **5ab**.

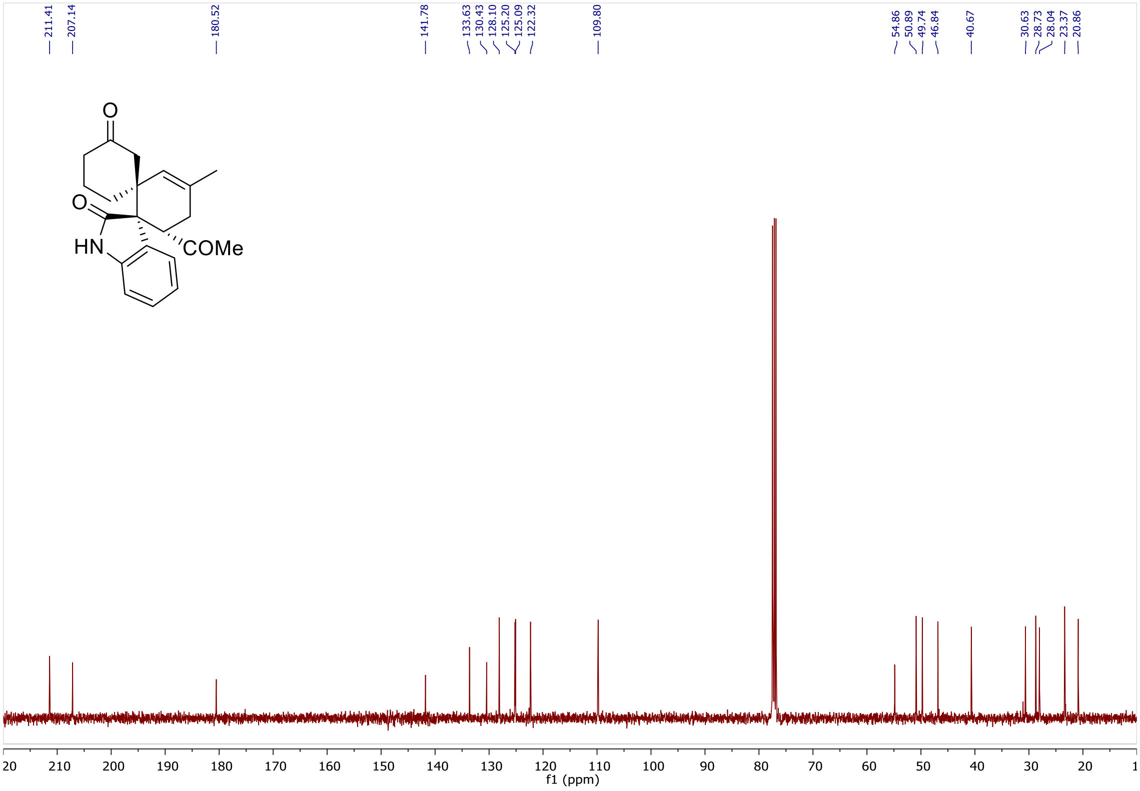

^{1}H NMR (400 MHz, $CDCl_3$) **5ac**.

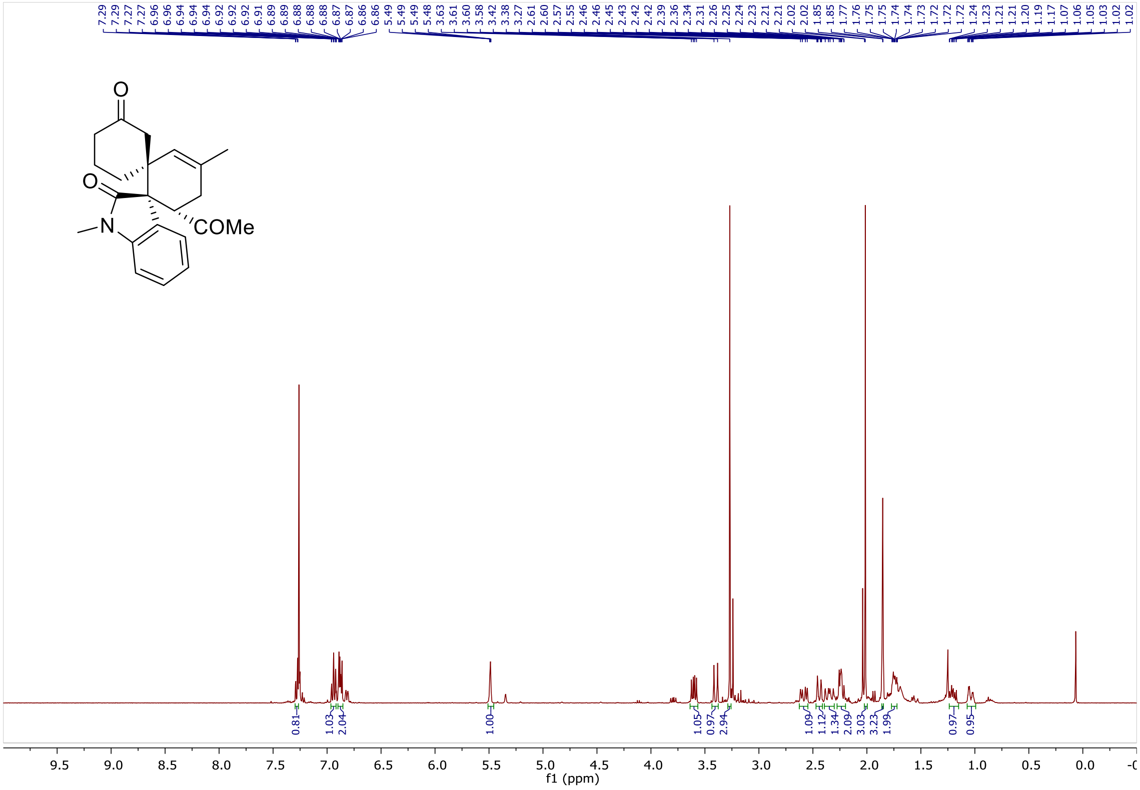

^{13}C {1H} NMR (101 MHz, $CDCl_3$) of **5ac**.

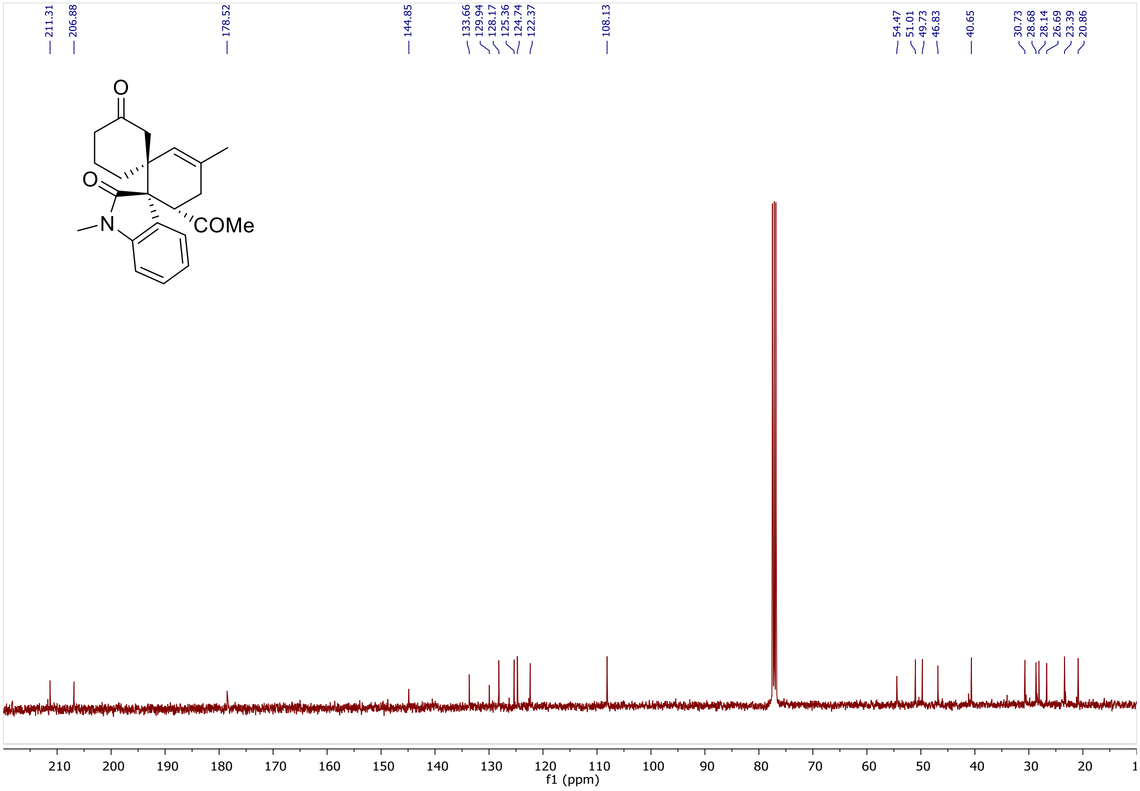

7. **X-Ray Diffraction Analysis of 3aa** (CCDC 2428417).

The crystal **3aa** was crystallized via slow evaporation of the sample dissolved in a minimum volume of Hex:DCM or Hex:$CHCl_3$.

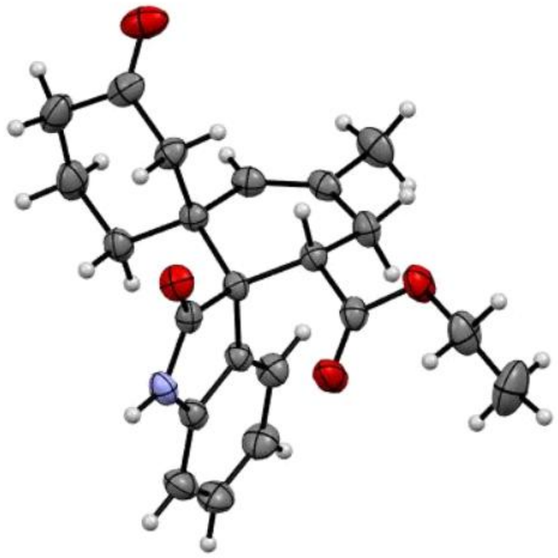

Figure S1. ORTEP diagram with thermal ellipsoids in 50% probability for **3aa**.

checkCIF/PLATON report

Structure factors have been supplied for datablock(s) str2319

THIS REPORT IS FOR GUIDANCE ONLY. IF USED AS PART OF A REVIEW PROCEDURE FOR PUBLICATION, IT SHOULD NOT REPLACE THE EXPERTISE OF AN EXPERIENCED CRYSTALLOGRAPHIC REFEREE.

No syntax errors found. CIF dictionary Interpreting this report

Datablock: str2319

```
Bond precision:    C-C = 0.0021 A          Wavelength=1.54184

Cell:              a=9.19431(10)     b=14.9770(2)        c=13.78456(14)
                   alpha=90          beta=93.8038(10)    gamma=90
Temperature:       220 K

                   Calculated                  Reported
Volume             1894.00(4)                  1894.00(4)
Space group        P 21                        P 21
Hall group         P 2yb                       ?
Moiety formula     C22 H25 N O4                C22 H25 N O4
Sum formula        C22 H25 N O4                C22 H25 N O4
Mr                 367.43                      367.43
Dx,g cm-3          1.289                       1.289
Z                  4                           4
Mu (mm-1)          0.715                       0.715
F000               784.0                       784.0
F000'              786.40
h,k,lmax           11,18,16                    11,18,16
Nref               7429[ 3865]                 7099
Tmin,Tmax          0.774,0.823                 0.289,1.000
Tmin'              0.774

Correction method= # Reported T Limits: Tmin=0.289 Tmax=1.000
AbsCorr = GAUSSIAN

Data completeness= 1.84/0.96        Theta(max)= 71.930

R(reflections)= 0.0343( 7011)                          wR2(reflections)=
                                                       0.0870( 7099)
S = 1.087                  Npar= 491
```

Datablock str2319 - ellipsoid plot

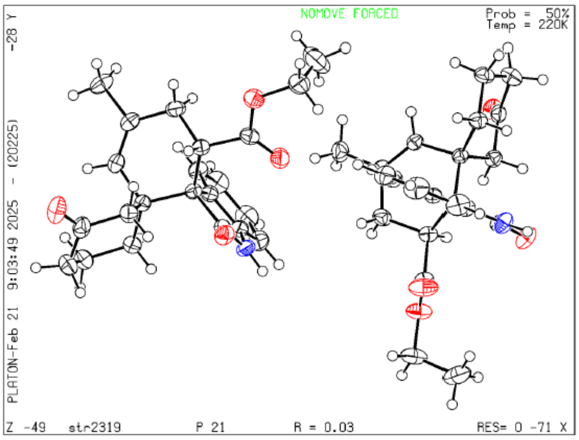

8. Electronic Circular Dichroism of 3ba''.

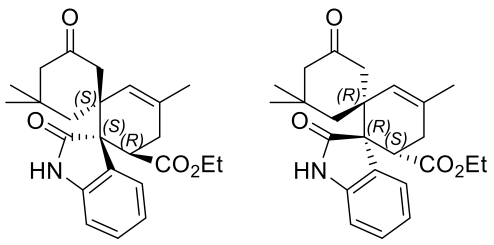

Enantiomer 1 Enantiomer 2

The conformational space of the molecule was studied through the following steps:

1. A search for stable conformers was conducted using XTB/ORCA version 6.0, implementing GOAT.
2. A total of 43 conformers were extracted, representing 100% of the population according to the Boltzmann distribution. The energy threshold to discard a stable conformer was set at 10 kcal/mol.
3. The geometry of these 43 conformers was optimized using the ORCA 5.0.2 DFT code [Comput. Mol. Sci., 2, 73–78] with the B3LYP/def2-TZVP method. Solvent effects (acetonitrile) were considered during geometry optimization using the CPCM method. After optimization, vibrational frequencies and Gibbs free energies were calculated for each conformer.
4. Once the conformer geometries were obtained using the above method, their energy values were refined through single-point energy calculations using the double-hybrid B2PLYP functional and the def2-QZVP basis set. Based on the results, the population of each conformer was updated.

ECD Spectrum Prediction

The ECD spectrum was predicted using TD-DFT theory with the ORCA 5.0.2 software, employing the double-hybrid B2PLYP functional and the Def2-QZVP basis set. The first 20 excitations were calculated. Solvent effects (acetonitrile) were included in the calculations. The Tamm-Dancoff approximation was disabled, as it can lead to erroneous signals in the calculation of dichroism spectra [*J. Phys. Chem. A*. **2015**, *119*, 3653–3662].

ECD spectra were calculated for each conformer, and the overall spectrum was weighted according to the previously calculated relative populations. Consistency and similarity were observed in the position, shape, and intensity of the dichroic bands across all conformers, as expected due to the high structural rigidity of the molecule revealed in the prior conformational study.

Experimental

The experimental ECD spectrum was recorded using a Jasco J-810 spectropolarimeter. The analysis range covered 200 to 400 nm. A baseline correction was performed using pure solvent (acetonitrile). The final spectrum of the sample was obtained using a concentration of 0.0001 M, with three cumulative scans recorded for the same sample.

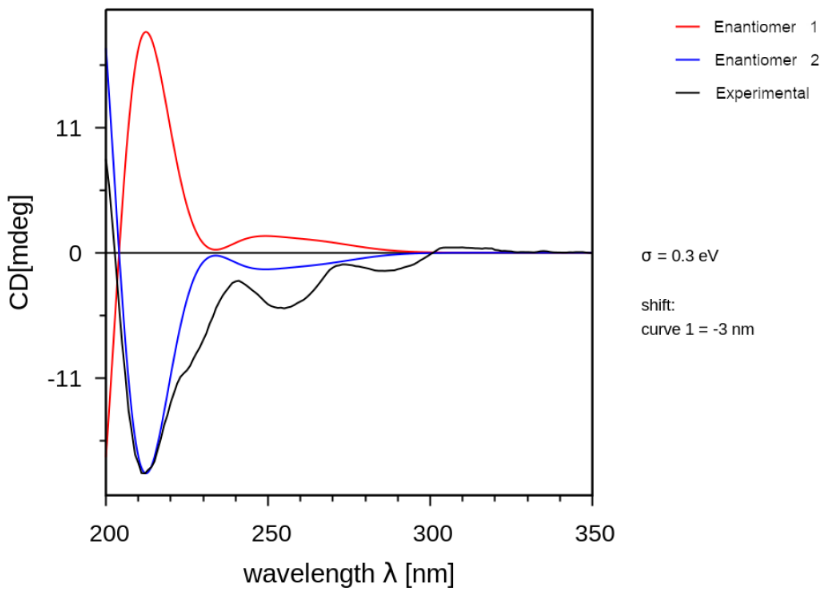

The above graph shows a comparison between the simulated circular dichroism values of the two enantiomers (red and blue lines) and the experimental circular dichroism of sample RH710F1.

Similarity Coefficient

These coefficients indicate the degree of similarity (1 means identical, 0 means very different) resulting from the comparison between the experimental data and the theoretical predictions for each of the isomers.

Similarity factor Enantiomer 1 = 0.002
Similarity factor Enantiomer 2 = 0.937
Delta value: 0.935

The result indicates a stronger correlation between the experimental ECD spectrum and the theoretical spectrum of Enantiomer 2.
